# Leveraging large language models to populate structured clinical case report forms from unstructured medical notes in radiation oncology

**DOI:** 10.1016/j.ctro.2026.101143

**Published:** 2026-03-09

**Authors:** Marcel Nachbar, Nianzi Yi, Marcel Büttner, Cihan Gani, Maximilian Niyazi, Augusto Garcia-Agundez, Carsten Eickhoff, Daniela Thorwarth

**Affiliations:** a Section for Biomedical Physics, Department of Radiation Oncology, University Hospital and Medical Faculty, Eberhard Karls University Tübingen, Hoppe-Seyler-Str. 3 72076, Tübingen, Germany; b Institute for Bioinformatics and Medical Informatics, Eberhard-Karls-Universität Tübingen, Tübingen, Germany; c Department of Radiation Oncology, University Hospital Tübingen, Tübingen, Germany; d German Cancer Consortium (DKTK), partner site Tübingen, and German Cancer Research Center (DKFZ), Heidelberg, Germany; e University of California San Francisco, CA, United States; f Cluster of Excellence ‚Machine Learning in the Sciences, University of Tübingen, Germany

## Abstract

**Background and purpose:**

Large language models (LLMs) have shown growing potential for clinical text processing, but their systematic application in radiation oncology—especially for non-English clinical documentation—remains underexplored. This study investigated whether pretrained LLMs can automatically extract, analyze, and structure radiotherapy-relevant information from routine unstructured medical notes, with the goal of supporting automated population of electronic case report forms (eCRFs).

**Materials and methods:**

This study examined prostate cancer patients treated with the MR-Linac, for whom ground truth data exist in the MOMENTUM database. A total of 100 patients were included, with 90 used for prompt development and 10 for independent testing. Medical notes were extracted, anonymized, and categorized by time points. The Llama-3.1-8b model was used, with prompts designed using chain-of-thought (CoT) logic with five in-context examples. The model output was post-processed, and extracted data was compared against ground truth.

**Results:**

Medical notes were successfully processed, with predicted values generated in an average time of 16 s per note. The LLM achieved matching accuracies of 83.6% and 83.8% on the development and testing datasets. Analysis revealed that the model disagreed with specific values in 8.1% of development dataset cases and 8.6% of testing dataset cases. An independent manual review before model evaluation showed approximately 7.5% of routinely collected test data did not match reviewed values, indicating inaccuracies in the routinely acquired ground truth.

**Conclusion:**

This study demonstrated the effectiveness of LLMs in structuring clinical data from medical non-English notes, with high accuracy in extracting and categorizing information. While multi-institutional validation is needed, the results indicate a significant healthcare impact through efficient data management, processing notes in 16 s, and accurately populating CRFs with minimal staff involvement.

## Highlights

• Large language models (LLMs) can automatically extract and structure data from unstructured medical notes with an average time of 16 s per note.
• The LLM achieved matching accuracies of 83.6% and 83.8% on the development and testing datasets, respectively.
• In-depth analysis revealed model disagreement with specific values in 8.1% (development) and 8.6% (testing) of cases.
• Manual review found ∼7.5% of routine test data mismatched reviewed values, indicating inaccuracies in the routine ground truth.

## 1 Introduction

Artificial Intelligence (AI) is a rapidly evolving technology with transformative applications across various domains. In radiation oncology, image-based AI has emerged as a significant advancement, enabling more resource-efficient and accurate contouring of structures, synthetic computed tomography (CT) generation, and dose predictions [1], [2]. However, the potential of large language models (LLMs) in radiation oncology remains less well established. Although systems such as ChatGPT are increasingly used in everyday practice, their application to structured clinical tasks is still limited by their sensitivity to complex prompts and the risk of generating hallucinations.

Despite these challenges, several avenues are being explored to leverage pretrained LLMs in oncology. One such approach, demonstrated by Dennstädt et al. [3], involves using ChatGPT as a physician-like assistant to answer multiple-choice questions in clinical, physics, and biology. Other groups have investigated LLMs in scenarios where they act as conversational substitutes for physicians [4], [5], or as tools to support or automate medical note writing [6]. Beyond conversational uses, recent studies have evaluated LLM-driven information extraction. Choi et al. extracted a limited set of CRF-relevant parameters (up to 12) from structured pathology and ultrasound reports, which represent relatively standardized diagnostic documents [7]. Park et al. used prompting to structure large-scale unstructured electronic health record text to support mortality prediction, focusing on extracting a small number of variables relevant to that specific outcome radiation-oncology–specific applications, Khanmohammadi et al. focused on iterative prompt refinement to improve the extraction of predefined symptoms, demonstrating methodological advances but remaining confined to a highly targeted task [8], [9].

Current radiation oncology research remains limited to extracting small, predefined variables from specific reports. A key research gap exists in using LLMs to extract broader radiotherapy-relevant parameters from routine medical notes, which contain richer contextual information than pathology reports or structured EHR fields. Bittermann et al. [10] provided first evidence for such extraction using hybrid transformer–rule-based models, but their approach required substantial task-specific engineering, limiting adaptability across institutions and disease sites.

All existing studies investigating LLM-based information extraction in radiation oncology have been limited to English-language datasets. This represents a relevant constraint, as many radiotherapy departments—particularly in Europe—document predominantly in non-English languages. Such documentation includes linguistic and terminological characteristics that are sparsely represented in current LLM training corpora and may therefore reduce extraction reliability.

This issue is particularly pertinent for multi-centre data collection. Large initiatives such as the MOMENTUM study [11] prospectively record detailed clinical and technical MR-Linac information to support hypothesis testing [12], [13], [14], [15]. Nevertheless, completing electronic case report forms (eCRFs) remains resource-intensive, and a considerable proportion of clinically relevant information remains available only within narrative clinical notes.

Therefore, this study aimed to investigate the feasibility of using pretrained LLMs to extract and structure radiotherapy-relevant information from routine non-English medical notes. The objective of this proof-of-concept was to demonstrate that a general-purpose, prompt-based approach—without model retraining—can populating eCRF databases for future hypothesis generation.

## 2 Materials and methods

### Study Population

In this study, 100 patients with prostate cancer treated with radiotherapy at the MR-Linac between 01/2019 and 11/2024 were included. Patients were included consecutively according to their entry dates in the MOMENTUM database [11] (IRB approval number 059/2023B01). The MOMENTUM study is listed on ClinicalTrials.gov with the identifier NCT04075305 (https://clinicaltrials.gov/ct2/show/NCT04075305). Since the MOMENTUM protocol allows for retrospective inclusion, the number of follow-up visits varied among patients at the time of assessment. Typically, any patient receiving treatment with the MR-Linac is eligible to participate in the MOMENTUM study and has provided written informed consent. In this study, only eCRFs added into the MOMENTUM database by study nurses and physicians were considered. Based on the available data, the patient cohort within the evaluation was divided into two groups, based on the study inclusion date, without additional stratification: 1) the patient cohort for prompt engineering and development, comprising the first 90 patients; and 2) an independent testing cohort consisting of patients 91–100, which was not included in the development set.

### Data Collection

Medical notes in German language were extracted from the hospital’s EHR system via the local data lake. The medical notes, written by multiple different physicians, in.docx format were subsequently converted to.txt files, and patient names were anonymized using a self-developed pattern recognition Python (V. 3.12) script. In a manual step, the notes were categorized into the corresponding time points of data collection with respect to radiation treatment (baseline, 3, 6, 12, and 24 months follow-up), and a pseudonymized folder structure was maintained for analysis against the gold standard. For comparison, the ground-truth data were extracted from the MOMENTUM database in one batch containing all previously filled eCRF information. For prompt development, clinically established values from the MOMENTUM database were used without performing a complete record-by-record verification. Instead, selected entries were spot-checked to assess general data validity, and any identified discrepancies were corrected during the prompt design phase.. For the testing cohort, ground-truth labels were initially entered by one rater and subsequently independently verified by an experienced medical physicist for each patient and time point. Any discrepancies were resolved by the medical physicist, whose judgement was considered final, and the corrected values were used as the reference labels. In addition, the frequency of discrepancies between the initial entries and the verified reference labels was documented and evaluated.

### LLM Processing

The pseudonymized text documents and ground truth CSV file from the MOMENTUM database were subsequently transferred to a cloud system. An automatic pipeline was developed within this environment, utilizing the configured model and defined prompts. In this proof-of-concept evaluation, an offline snapshot of the Llama-3.1-8b Interact model was used [16]. The model was initialized, and for each medical note, the corresponding prompt was selected based on the associated time point. Role prompting was subsequently used to generate the respective outputs. The prompt text was designed using a chain-of-thought approach and included five in-context examples. All prompts used in this study, organized by follow-up time point, are provided in Supplementary Text S2. For each analyzable parameter, the prompt instructed the model to assign a predefined default value if the corresponding information could not be identified in the source text. During model inference, a self-consistency approach was applied by generating five independent output sequences, with the final result determined by a majority vote across these outputs (Supplementary Text S1). The main model configuration is summarized in Supplementary Table S1. The main model configuration is summarized in Supplementary Table S1. To assess the influence of model size on performance, the same processing pipeline was additionally evaluated using the LLaMA 3.1-70B model.

The consolidated model output was then scraped and postprocessed. During post-processing, homogenization was performed by correcting data formats, ensuring that all values corresponded to the same time format (DD-MM-YYYY), and missing values were filled with NaNs for the analysis. Subsequently, within an analysis script, the extracted data were compared with the ground truth, and the accuracy was evaluated. This script was executed as batch on NVIDIA H100 Tensor Core GPUs with 50 GB of GPU RAM and timed from job submission to the end of the data analysis.

### Data Extraction and Comparison

For each patient, 61 clinical parameters were available for extraction from the medical notes. These clinical parameters, as shown in the Supplementary Table S2, include the planned start date, clinical tumor stage, PSA values, Gleason score, and Eastern Cooperative Oncology Group (ECOG) score. For certain parameters, such as the Karnofsky score (KPSS), PSA value, or ECOG, specific values were independently evaluated based on their scoring time-points. Consequently, there are five different PSA value pairs for baseline and for follow-up at 3, 6, 12, and 24 months.

In the in-depth analysis, each parameter was compared to determine whether the LLM-leveraged parameter matched the ground truth and was evaluated in relation to the default, resulting in six independent scenarios based on clinical significance:

• Scenario 1: Match on Value (MOV): LLM and ground truth identify and agree on a specific value.
• Scenario 2: Match on Default (MOD): Both sources do not detect a specific value and default to “Unknown.”
• Scenario 3: LLM Adds Value on Default (LAD): The ground truth defaults to “Unknown,” whereas the LLM identifies a specific value, potentially indicating a missed entry in the reference.
• Scenario 4: LLM Adds Value on Not Filled (LANF): The ground truth field is unfilled or empty, whereas the LLM identifies a specific value, potentially indicating a missed entry in the reference.
• Scenario 5: Ground Truth Specific, LLM Default (GSD): Ground truth contains a valid, specific value, but the LLM returns “Unknown” or a default — suggesting a potential missed extraction or high uncertainty.
• Scenario 6: Mismatch on Value (MMV): The ground truth contains a specific value, but the LLM extracts a different value.

In addition, for all non-matches, we assess whether the LLM-extracted value falls within clinically acceptable limits, using ± 14 days for dates and ± 0.2 ng/mL for PSA values.

The same methodology was employed to evaluate the values extracted automatically from the MOMENTUM database, with the validation dataset independently verified by an expert using identical scoring criteria. This process assessed the performance limitations of the LLM compared to routine human observers.

## 3 Results

The development dataset (90 patients) included 287 medical notes: 90 baseline notes and 70, 38, 52, and 37 notes at 3, 6, 12, and 24 months, respectively. The test dataset (10 patients) comprised 36 notes, distributed as 10, 9, 5, 6, and 6 across the same time points. In total, 3,385 of 5,490 (62%) possible parameters were documented in the development dataset and 395 of 610 (65%) in the test dataset. Each patient had 61 parameters available for evaluation, although follow-up variability meant not all parameters were present for every patient. The least documented parameters were the 6-month ECOG date and score and the 24-month Karnofsky date and score (9 values each). An overview of all parameters and their distributions is provided in the Supplementary Table S2.

Using the LLM, we processed all 287 medical notes from the development database and 36 notes from the testing database within 76 min and 51 s and 9 min and 59 s, respectively, corresponding to an average time of 16 s per medical note.

**Fig. 1:**
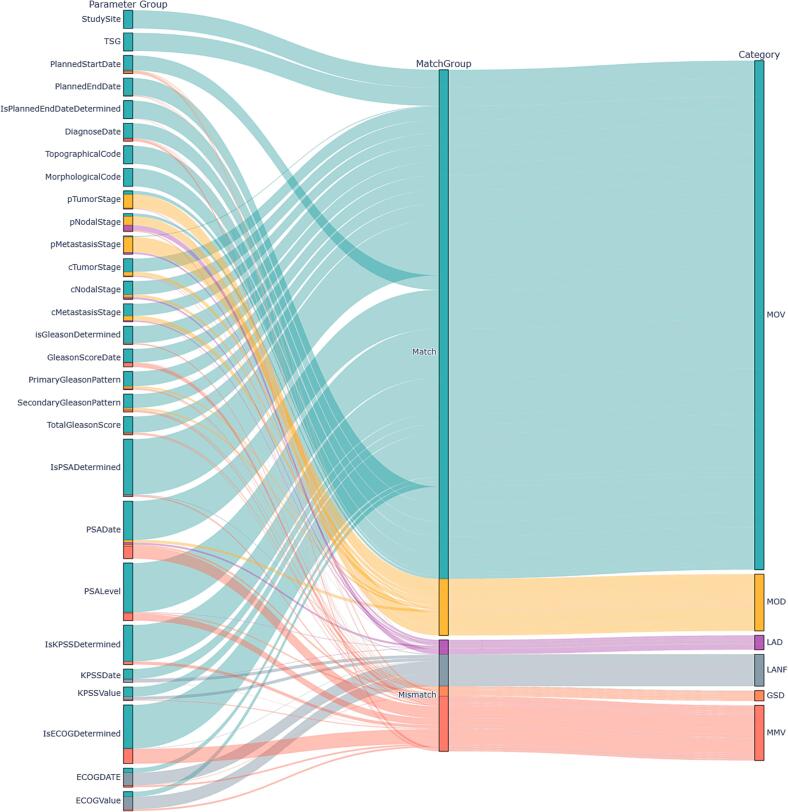
Sankey diagram illustrating the parameters and their respective evaluations against the ground truth data for the development dataset. Each parameter's inclusion within the match/mismatch criteria, as well as the corresponding in-depth scenario, is depicted. For enhanced clarity, related parameters, such as PSA, KPSS, and ECOG, were aggregated across all time points. Categories are categorized into predefined scenarios**: MOV** (Match on Value), **MOD** (Match on Default), **LAD** (LLM Adds on Default), **LANF** (LLM Adds on Not Filled), **GSD** (Ground Truth Specific, LLM Default), and **MMV** (Mismatch on Value).

**Fig. 2:**
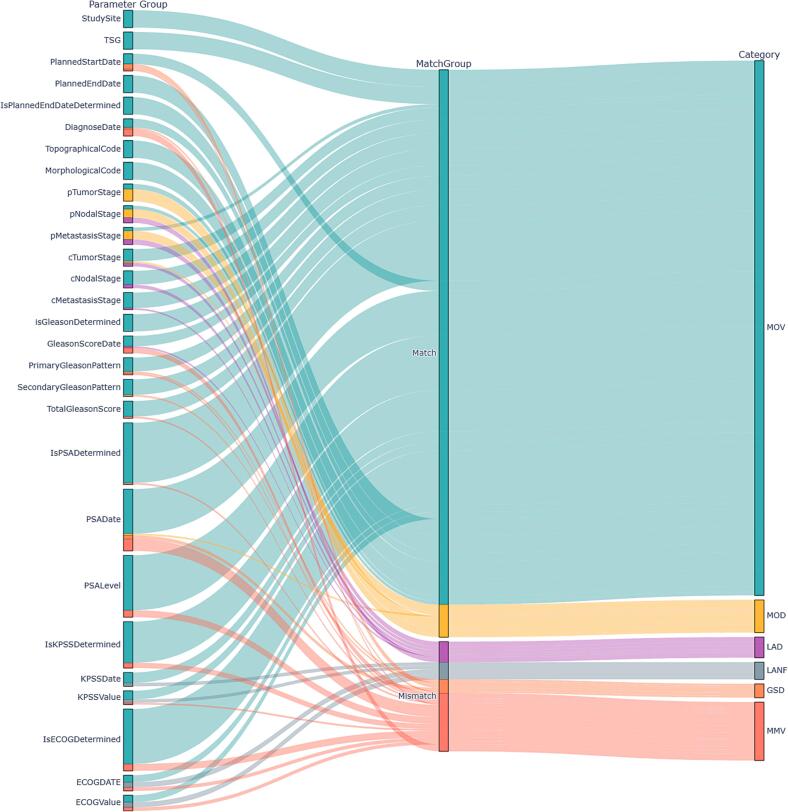
The Sankey diagram illustrates the parameters and their respective evaluations against the gold standard data for the validation dataset. Each parameter's inclusion within the match/mismatch criteria, as well as the corresponding in-depth scenario, is depicted. For enhanced clarity, related parameters, such as PSA, KPSS, and ECOG, were aggregated across all time points. Categories are categorized into predefined scenarios**: MOV** (Match on Value), **MOD** (Match on Default), **LAD** (LLM Adds on Default), **LANF** (LLM Adds on Not Filled), **GSD** (Ground Truth Specific, LLM Default), and **MMV** (Mismatch on Value).

Within the development dataset, an average of 83.6% of all evaluated parameter pairs matched, and 83.8% matched within the validation dataset. Upon conducting a comprehensive evaluation, as illustrated in Fig. 1, Fig. 2, as well as Tables 1 and S3, it was observed that the LLM-predicted parameters exhibited discrepancies in specific values in only 8.1% of the development cases and 8.6% of the testing cases. A detailed analysis revealed that the LLM provided an additional specific non-default value when the database defaulted in 2.1% of the development cases and 3.0% of the testing cases, or filled the database with a value when it was initially unfilled in 4.7% of the development cases and 2.5% of the testing cases.

**Table 1:** This table provides a detailed analysis of the LLM-based eCRF extraction results for the development dataset in comparison to gold-standard answers, structured by predefined evaluation scenarios. It reports the percentage of direct matches (matches/total) for each parameter. The parameters are grouped into the following scenarios: MOV (Match on Value), MOD (Match on Default), LAD (LLM Adds on Default), LANF (LLM Adds on Not Filled), GSD (Ground Truth Specific, LLM Default), and MMV (Mismatch on Value). The column **“Within Limit”** indicates, for each parameter, how many mismatched values fall within the predefined acceptable value range (±0.2 for PSA and ± 14 days for dates). To support the identification of critical parameters, the lowest-performing 25% of parameters, based on the match rate, are highlighted in red.

At the parameter level, the exact matching accuracy ranged from 100% (StudySite, Tumor site group (TSG), MorphologicalCode) to 17% (Baseline ECOG/FU3M_ECOG_Date) in the development database. Multiple time points of the ECOG data were evaluated with a matching accuracy below 40% for the development dataset, prompting validation of the Baseline ECOG date. This validation highlighted that out of the 24 unfilled parameters within the ground truth, 23 instances were noted in the note but not entered by the human observer but found by the LLM. Further details are provided in Table 2.

**Table 2:** This table presents a comprehensive analysis of ECOG data in the training dataset, comparing evaluation scenarios with corresponding database responses (golden data derived from an LLM-based approach). The visualization focuses exclusively on non-matching scenarios where the golden data is 'nan' and the LLM predicts a value. Columns 6–8 provide additional validation of this data within the training dataset to enhance the understanding of the offset.

**Patient**	**Golden**	**LLM**	**Match**	**Ecog exists**	**Validated Values**	**AI-Value Matches the validated data**
Patient002	NaN	09–11-2023	❌	correct	09–11-2023	✅
Patient009	NaN	09–08-2023	❌	correct	23–08-2023	❌
Patient010	NaN	27–10-2023	❌	correct	27–10-2023	✅
Patient014	NaN	03–07-2024	❌	correct	03–07-2024	✅
Patient021	NaN	19–12-2023	❌	correct	19–12-2023	✅
Patient026	NaN	01–01-1901	❌	correct	15–03-2023	❌
Patient027	NaN	09–01-2023	❌	correct	09–01-2023	✅
Patient028	NaN	11–08-2023	❌	correct	11–08-2023	✅
Patient030	NaN	09–03-2022	❌	incorrect		❌
Patient032	NaN	07–03-2024	❌	correct	07–03-2024	✅
Patient037	NaN	23–02-2023	❌	correct	23–02-2023	✅
Patient042	NaN	08–12-2023	❌	correct	19–12-2023	❌
Patient059	NaN	05–11-2024	❌	correct	05–11-2024	✅
Patient060	NaN	21–06-2024	❌	correct	21–06-2024	✅
Patient061	NaN	21–08-2024	❌	correct	21–08-2024	✅
Patient063	NaN	30–11-2023	❌	correct	30–11-2023	✅
Patient066	NaN	13–04-2023	❌	correct	12–05-2023	❌
Patient069	NaN	25–05-2023	❌	correct	25–05-2023	✅
Patient070	NaN	17–02-2023	❌	correct	22–03-2023	❌
Patient075	NaN	23–10-2023	❌	correct	23–10-2023	✅
Patient077	NaN	04–08-2023	❌	correct	11–08-2023	❌
Patient081	NaN	11–09-2020	❌	correct	16–06-2020	❌
Patient084	NaN	04–09-2023	❌	correct	04–10-2023	❌
Patient087	NaN	17–01-2023	❌	correct	16–02-2023	❌

Upon analyzing human error, further validation of the testing dataset indicated that 92.6% of the parameter values were verifiable, whereas 7.5% required modification. These modifications pertained to 31 distinct parameters, with most corrections required for the diagnosis and baseline ECOG dates. These corrections involved either adjusting a specific value or providing a specific value instead of the default or unfilled value. Additional details are provided in Table S4.

While the overall accuracy of the LLM model did not decline from the development to the validation database, minor shifts in the percentage distributions of the in-depth evaluation were observed, with a relative percentage on MOV of 75.2% versus 79%, MMV of 8.1% versus 8.6%, GSD 1.5% versus 2%, MOD at 8.4% versus 4.8%, LAD 2.1% versus 3%, and LANF at 4.7% versus 2.5% for the development and testing data, respectively. Table 3 presents an exemplary low-performing parameter within the testing data (diagnosis date) that existed for all 10 patients, along with the corresponding evaluation. The secondary evaluation using the larger model did not improve overall performance, as it produced a comparable number of MOV parameters while assigning more cases to the LANF scenario. Consequently, the absolute percentage match decreased to 82.2% in the development set and 80% in the testing set. Additional details are provided in Supplementary Table S5.

**Table 3:** The analysis presented offers a comprehensive examination of the diagnosis dates from the validation dataset, correlating them with their respective evaluation scenarios and the corresponding responses derived from the reference database (Golden data).

**Patient**	**Group**	**Golden**	**AI**	**Match**
Patient091	**MMV**	01–12-2019	12–19-2019	❌
Patient092	**MOV**	01–05-2020	01–05-2020	✅
Patient093	**MMV**	04–11-2020	15–01-2020	❌
Patient094	**MOV**	01–12-2020	01–12-2020	✅
Patient095	**MMV**	01–08-2009	01–01-2009	❌
Patient096	**MOV**	01–09-2024	01–09-2024	✅
Patient097	**MOV**	01–12-2022	01–12-2022	✅
Patient098	**GSD**	01–01-2024	01–01-1901	❌
Patient099	**MMV**	01–01-2017	01–05-2017	❌
Patient100	**MOV**	01–07-2024	01–07-2024	✅

## 4 Discussion

In this proof-of-concept study, we developed an automated data processing pipeline that leverages a large language model to extract structured electronic case report forms (eCRF) from unstructured medical notes. We achieved a matching accuracy of 83.6% on a prompting development dataset, which we validated with 83.8% accuracy on a testing dataset. An in-depth analysis of the failure scenarios revealed that the model disagreed with specific values (not default or unfilled) in 8.1% and 8.6% of the cases in the development and testing datasets, respectively.

To analyze this potential offset between the routinely acquired eCRF database values and the correct data, an additional analysis within the testing dataset was conducted, showing that the routinely acquired data were mismatched in approximately 7.5% of cases. This discrepancy likely reflects routine clinical documentation workflows, including data entry during ongoing documentation, subsequent corrections, observer-dependent interpretation, and occasional human error under time pressure when information is repeated, incomplete, or ambiguous (e.g., diagnosis dates documented multiple times or only partially specified).

Although encouraging, as shown in Fig. 1, the language model's effectiveness is influenced by predicted parameters, especially dates. It demonstrated varied performance, with 98% accuracy for planned start date, but only 17% for the 3-month follow-up ECOG date in the development dataset. Analysis of failures and medical notes suggests two issues. First, parameters may be falsely omitted, as shown in Table 2 for ECOG within the development dataset, or data can be extracted from different sections, depending on the observer. Contradictions occur within notes due to their unstructured nature when written under time pressure. A human observer could verify base information using primary data, like laboratory values. Another issue with dates is format variability between notes, with some dates abbreviated as 08/22 instead of exact dates. Parameters showing high performance are often better defined, such as study site and clinical tumor stage, due to clear definition and position within the protocol. For certain parameters, like study site and morphological stage, there was no data variability in the evaluated data due to single-center and single-entity evaluation, making generalizability interpretation challenging.

In this study, we chose the LLaMA 3.1–8B model for its strong performance relative to its compact size, enabling the efficient evaluation of prompting strategies on hardware that aligns with potential offline deployment constraints within clinical networks [17]. Although benchmark frameworks exist to assess general reasoning capabilities of LLMs [18], their predictive value for radiotherapy-specific information extraction is limited. Larger or more advanced models—such as higher-capacity LLaMA variants, Qwen models, or reinforcement-learning–enhanced architectures like DeepSeek—could offer improved robustness or more consistent reasoning behaviour. Approaches that explicitly structure intermediate reasoning steps may also help clarify whether additional reasoning depth provides measurable benefits. However, a comprehensive multi-model comparison was beyond the scope of this feasibility study, which aimed to evaluate the viability of the approach using a single openly available model. Assessing different model sizes and families should be addressed in future work. However, within the secondary evaluation against the LlaMa3.1 – 70B model no increase in performance was visible (Supplementary Table S5).

We developed the prompts stepwise with increasing complexity to maximize correct matches while minimizing hallucinations. To further control hallucination rates, we used a low temperature in combination with multiple return sequences and majority voting providing five examples per task and encouraging stepwise reasoning. To mitigate variability from stochastic decoding, each temperature setting was evaluated through at least two independent repetitions. While this procedure improves robustness, additional repetitions could further refine stability. During development, we tested several prompting variants and ultimately selected a CoT style based on preliminary performance. However, we did not systematically compare CoT with simpler prompts; given the largely straightforward information extraction tasks, CoT may not be strictly necessary and could add unnecessary complexity. Evaluating this trade-off—as well as the self-consistency approach—through larger-scale quantitative studies remains important future work. Another promising direction, beyond manual prompt development, is to employ multiple models in a teacher–student setup that iteratively optimizes prompts [8].

The model exhibited hallucinations by adding values where defaults were present in the validated testing dataset for 3% and filled in missing information for 2.5% of parameters, primarily due to confusion between pathological and clinical stages. Potentially implementing secondary filters, such as prompting for radical prostatectomy, could enhance the accuracy of these cases.

Our study focused on LLMs for extracting structured clinical information for eCRFs in Radiation Oncology. While recent literature shows no directly comparable work, studies highlight LLMs' expanding role in clinical applications. Hou et al. [19] found the LLaMA 3.1–8B model superior in generating physician notes. Dehelean et al. [20] used an LLM to educate meningioma patients, while Holmes et al. [21]evaluated LLM performance on radiation physics questions. In imaging and treatment planning, Oh et al. [22]improved segmentation accuracy using clinical data, Dong et al. [23]integrated an LLM to interpret prescriptions, and Wang et al. [24]automated optimization processes. These studies demonstrate LLMs’ growing integration in radiation oncology, yet none addressed high-scale extraction of heterogeneous structured parameters from non-English medical notes underscoring the novelty of our study.

However, a key limitation of this study is the evaluation on a single disease entity (prostate cancer) at a single center using German-language medical notes from one clinical department, together with a relatively small independent test set of 10 patients. As a result, the generalizability of the findings remains limited. Future analyses should incorporate larger test cohorts as well as multicenter and multilingual data to more comprehensively assess the robustness of the pipeline across diverse clinical settings.

## 5 Conclusion

This study demonstrated the effectiveness of LLMs in structuring clinical data from unstructured non-English medical text. The results indicate high accuracy in extracting key information and categorizing it into standardized formats. While the validity of these findings on multi-institutional and multilingual data still needs to be explored, these initial results suggest a significant impact on current healthcare practices. LLMs show potential for efficient data management, processing notes in 16 s each, accurately populating patient data while reducing manual input. This integration could shift staff from data entry to validation, thereby optimizing resources. However, given the rapidly advancing AI model performance and prompting techniques, further research is necessary to address parameter-specific data inaccuracies and the potential to adapt existing pipelines to other centers and tumor entities.

## Declaration of competing interest

The authors declare that they have no known competing financial interests or personal relationships that could have appeared to influence the work reported in this paper.
