# Supplementary material for: Leveraging large language models to populate structured clinical case report forms from unstructured medical notes in radiation oncology
Source: Clin Transl Radiat Oncol. 2026 Mar 9;58:101143. doi: 10.1016/j.ctro.2026.101143 (PMC12996807; doi:10.1016/j.ctro.2026.101143)
Supplement: Supplementary Data 1 [file mmc1.docx]

## Supplementary Table S1

Table S1: Overview of the primary configuration settings for the utilized LLM

| **Parameter** | **Value** |
| --- | --- |
| model | Llama-3.1-8B |
| max_new_tokens | 2048 |
| temperature | 0.3 |
| do_sample | true |
| top_p | 1.0 |
| top_k | 50 |
| num_beams | 1 |
| num_return_sequences | 5 |
| repetition_penalty | 1.0 |
| no_repeat_ngram_size | 0 |
| eos_token_id (stop) | tokenizer.eos_token_id |
| seed | not fixed |

## Supplementary Table S2

Table S2: Overview of the distribution of the patient eCRF for the 90 patients in the training dataset and 10 in the testing dataset. Coverage indicates the number of patients for whom an item was scored. The values represent the distribution of the corresponding dataset as a fraction of the possible answers or, for a date or free value, as the median and the range.

## Supplementary Table S3

Table S3: This table provides a detailed analysis of the LLM-based eCRF extraction results for the testing dataset in comparison to gold-standard answers, structured by predefined evaluation scenarios. It reports the percentage of direct matches (matches/total) for each parameter. The parameters are grouped into the following scenarios: MOV (Match on Value), MOD (Match on Default), LAD (LLM Adds on Default), LANF (LLM Adds on Not Filled), GSD (Ground Truth Specific, LLM Default), and MMV (Mismatch on Value). The column **“Within Limit”** indicates, for each parameter, how many mismatched values fall within the predefined acceptable value range (±0.2 for PSA and ±14 days for dates). To support the identification of critical parameters, the lowest-performing 25% of parameters, based on the match rate, are highlighted in red.

### Supplementary Table S4

Table S4: Comparison of routinely acquired data with the secondary validated dataset. Only the parameters showing a difference are visualized, while the “Total” and “Matches” represent all the given data. Parameters are categorized into predefined scenarios: **LAD** (LLM Adds on Default), **LANF** (LLM Adds on Not Filled), **GSD** (Ground Truth Specific, LLM Default), and  **MMV** (Mismatch on Value)

| **Parameter** | **LAD** | **LANF** | **GSD** | **MMV** | **Total** | **Matches** | **Mismatches** |
| --- | --- | --- | --- | --- | --- | --- | --- |
| DiagnoseDate | 0 | 0 | 0 | 6 | 10 | 4 | 6 |
| pMetastasisStage | 1 | 0 | 0 | 0 | 10 | 9 | 1 |
| cTumorStage | 1 | 0 | 0 | 0 | 10 | 9 | 1 |
| cMetastasisStage | 0 | 0 | 1 | 0 | 10 | 9 | 1 |
| isGleasonDetermined | 0 | 0 | 0 | 1 | 10 | 9 | 1 |
| PrimaryGleasonPattern | 0 | 0 | 1 | 0 | 10 | 9 | 1 |
| SecondaryGleasonPattern | 0 | 0 | 1 | 0 | 10 | 9 | 1 |
| PSADate | 0 | 0 | 0 | 1 | 10 | 9 | 1 |
| FU3M_PSADate | 0 | 0 | 0 | 2 | 9 | 7 | 2 |
| FU6M_PSADate | 0 | 0 | 0 | 1 | 5 | 4 | 1 |
| FU24M_IsPSADetermined | 0 | 0 | 0 | 1 | 6 | 5 | 1 |
| FU24M_PSADate | 0 | 0 | 0 | 1 | 6 | 5 | 1 |
| FU24M_PSALevel | 0 | 0 | 0 | 1 | 6 | 5 | 1 |
| BASE_ECOG_YN | 0 | 0 | 0 | 2 | 10 | 8 | 2 |
| BASE_ECOG_DATE | 0 | 0 | 0 | 4 | 5 | 1 | 4 |
| BASE_ECOG | 0 | 0 | 0 | 2 | 5 | 3 | 2 |
| FU3M_ECOG_YN | 0 | 1 | 0 | 0 | 10 | 9 | 1 |
| FU6M_ECOG_YN | 0 | 3 | 0 | 0 | 8 | 5 | 3 |
| **…** | **…** | **…** | **…** | **…** | **…** | **…** | **…** |
| Sum | 2 | 4 | 3 | 22 | 416 | 385 | 31 |
| Relative Percentage | 0,48% | 0,96% | 0,72% | 5,29% | 100,00% | 92,55% | 7,45% |

### Supplementary Table S5

*Table S5: Comparison of the performance of the Llama 3.1 8B and 70B models on the development and test datasets, shown as absolute numbers of evaluated parameters (top) and relative proportions (bottom). Parameters are grouped into the following scenarios: MOV (Match on Value), MOD (Match on Default), LAD (LLM Adds on Default), LANF (LLM Adds on Not Filled), GSD (Ground Truth Specific, LLM Default), and MMV (Mismatch on Value). The column “Within Limit” reports, for each parameter, how many mismatched values fall within the predefined acceptable range (±0.2 for PSA, ±14 days for dates).*

| Absolut evaluated parameters | | | | | | | | | | | | | | | | | | | | | | |
| --- | --- | --- | --- | --- | --- | --- | --- | --- | --- | --- | --- | --- | --- | --- | --- | --- | --- | --- | --- | --- | --- | --- |
| Model | | Dataset | | MOV | | MOD | | LAD | | LANF | GSD | | MMV | | **Total** | | **Matches** | | **Mismatches** | | **Within Limit** | |
| Llama 8b | | Training | | 2545 | | 283 | | 72 | | 159 | 51 | | 275 | | 3385 | | 2828 | | 557 | | 34 | |
| Llama 70b | | Training | | 2544 | | 337 | | 16 | | 280 | 51 | | 276 | | 3504 | | 2881 | | 623 | | 39 | |
| Llama 8b | | Validation | | 312 | | 19 | | 12 | | 10 | 8 | | 34 | | 395 | | 331 | | 64 | | 3 | |
| Llama 70b | | Validation | | 299 | | 28 | | 3 | | 24 | 15 | | 40 | | 409 | | 327 | | 82 | | 4 | |
| Relative evaluated parameters | | | | | | | | | | | | | | | | | | | | | | |
| Model | Dataset | | MOV | | MOD | | LAD | | LANF | | | GSD | | MMV | | **Total** | | **Matches** | | **Mismatches** | | **Within Limit** |
| Llama 8b | Training | | 75.2% | | 8.4% | | 2.1% | | 4.7% | | | 1.5% | | 8.1% | | 100.0% | | 83.6% | | 16.5% | | 1.0% |
| Llama z0b | Training | | 72.6% | | 9.6% | | 0.5% | | 8.0% | | | 1.5% | | 7.9% | | 100.0% | | 82.2% | | 17.8% | | 1.1% |
| Llama 8b | Validation | | 79.0% | | 4.8% | | 3.0% | | 2.5% | | | 2.0% | | 8.6% | | 100.0% | | 83.8% | | 16.2% | | 0.8% |
| Llama 70b | Validation | | 73.1% | | 6.9% | | 0.7% | | 5.9% | | | 3.7% | | 9.8% | | 100.0% | | 80.00% | | 20.1% | | 1.0% |

### Supplementary Text S1 : Majority-vote aggregation of JSON outputs from multiple model generations

def majority_vote(json_list):

    """

    select the most common JSON object from 5 generated JSON as the final answer of current data field

    """

    json_strs = [json.dumps(j, sort_keys=True) for j in json_list if j]

    if not json_strs:

        return {}

    majority_vote_json, _ = Counter(json_strs).most_common(1)[0]

    return json.loads(majority_vote_json)
