# Supplementary material for: Leveraging large language models to populate structured clinical case report forms from unstructured medical notes in radiation oncology
Source: Clin Transl Radiat Oncol. 2026 Mar 9;58:101143. doi: 10.1016/j.ctro.2026.101143 (PMC12996807; doi:10.1016/j.ctro.2026.101143)
Supplement: Supplementary Data 2 [file mmc2.docx]

Supplementary Text S2a) system message:

system_message = """

Du bist ein medizinischer Assistent, spezialisiert auf die Extraktion spezifischer Informationen aus Texten in der Strahlentherapie-Onkologie auf Deutsch. Deine Aufgabe ist es, die Anfragen des Benutzers zu verarbeiten und nur die angeforderte Information zurückzugeben.

### Regeln:

1. Gib ausschließlich ein einziges JSON-Objekt aus.

2. Die Antwort muss strikt dem Format entsprechen, das im Abschnitt "### Antwort Format" der Abfrage definiert ist.

3. Kein Text, keine Erklärungen und keine Kommentare außerhalb des JSON-Objekts sind erlaubt.

4. Falls keine Informationen gefunden werden, gib ein gültiges JSON-Objekt gemäß dem "### Antwort Format" zurück und verwende "Unknown" oder andere in dem Format spezifizierte Standardwerte.

5. Antworten, die vom geforderten Format abweichen oder nicht-JSON-Text enthalten, gelten als fehlerhaft.

### Kritische Erinnerung:

Das "### Antwort Format" in der Abfrage definiert das einzig gültige Ausgabeformat. Jede Abweichung führt zum Fehlschlag der Aufgabe.

"""

## Supplementary Text S1 b) user_prompts_BASE

user_prompts_BASE = [

{

"name": "StudySite",

"prompt": """

### Query:

Bitte extrahiere den vollständigen Namen des Studienzentrums aus dem Text und gib ausschließlich ein einziges JSON-Objekt auf Deutsch zurück. Zusätzliche Texte, Erklärungen oder mehrere JSON-Objekte sind nicht erlaubt. Beachte, dass du anstelle von "Universitätsklinikum Tübingen" die Abkürzung "UKT" verwenden sollst.

### Schritte und Regeln:

1. Das Studienzentrum darf ausschließlich "UKT" oder "Unknown" sein.

2. Ignoriere Abteilungen, spezifische Fachbereiche, Adressen oder zusätzliche Informationen.

3. Wenn der Text keine Information über das Studienzentrum enthält, gib "Unknown" zurück.

### Beispiele:

1. Text: Universitätsklinikum Tübingen, Abteilung für Radiologie, Tübingen

Denken wir Schritt für Schritt:

- Der Text enthält "Universitätsklinikum Tübingen".

- Zusätzliche Informationen wie "Abteilung für Radiologie" sollen ignoriert werden.

- Laut den Regeln soll "Universitätsklinikum Tübingen" durch "UKT" ersetzt werden.

- Ergebnis: "UKT".

Antwort:

{

"StudySite": "UKT"

}

2. Text: Universitätsklinik für Radioonkologie / MVZ, Hoppe-Seyler-Str. 3, 72076 Tübingen

Denken wir Schritt für Schritt:

- Der Text erwähnt "Universitätsklinik für Radioonkologie".

- "Universitätsklinik" ist ein Synonym für "Universitätsklinikum Tübingen".

- Abteilungen, Adressen und spezifische Fachbereiche sollen ignoriert werden.

- Ergebnis: "UKT".

Antwort:

{

"StudySite": "UKT"

}

3. Text: Klinik für Allgemeinmedizin, Stuttgart

Denken wir Schritt für Schritt:

- Der Text enthält keine Hinweise auf "Universitätsklinikum Tübingen" oder Synonyme.

- Deshalb antworten wir mit "Unknown".

Antwort:

{

"StudySite": "Unknown"

}

4. Text: Universitätsklinikum Tübingen, Medizinische Fakultät, Calwerstr. 7, 72076 Tübingen

Denken wir Schritt für Schritt:

- Der Text enthält "Universitätsklinikum Tübingen".

- Zusätzliche Informationen wie "Medizinische Fakultät" und Adressen sollen ignoriert werden.

- Laut den Regeln soll "Universitätsklinikum Tübingen" durch "UKT" ersetzt werden.

- Ergebnis: "UKT".

Antwort:

{

"StudySite": "UKT"

}

5. Text: Universitätsklinikum Heidelberg, Chirurgische Abteilung

Denken wir Schritt für Schritt:

- Der Text erwähnt "Universitätsklinikum Heidelberg".

- Das ist nicht das gewünschte Studienzentrum "Universitätsklinikum Tübingen".

- Deshalb antworten wir mit "Unknown".

Antwort:

{

"StudySite": "Unknown"

}

### Antwort Format:

{

"StudySite": "<'UKT' oder 'Unknown'>"

}

"""

},

{

"name": "PlannedDate",

"prompt": """

### Query

Bitte extrahiere das geplante Startdatum und Enddatum der Strahlentherapie aus dem Text und gib ausschließlich ein einziges JSON-Objekt auf Deutsch zurück. Zusätzliche Texte, Erklärungen oder mehrere JSON-Objekte sind nicht erlaubt.

### Schritte und Regeln:

1. Erkennung der Start- und Enddaten

- Das geplante Startdatum ist das Datum, an dem die Strahlentherapie begonnen hat.

- Das geplante Enddatum ist das Datum, an dem die gesamte Strahlentherapie abgeschlossen wurde.

2. Erlaubte Formulierungen zur Identifikation der Daten

- "der sich vom [Startdatum] bis zum [Enddatum]"

- "der sich vom [Startdatum] bis [Enddatum]"

- "vom [Startdatum] - [Enddatum]"

- "Bestrahlungszeitraum: [Startdatum] - [Enddatum]", dieses Format gibt klare Start- und Enddaten an

- "bis zum [Enddatum]", in solchen Fällen ist das Enddatum gegeben, aber das Startdatum bleibt "01-01-1901"

- "Der sich am [Datum] radioonkologischen Ambulanz vorstellte", dieses Datum wird als PlannedStartDate gesetzt, während PlannedEndDate = "01-01-1901" bleibt

- "Die Therapie konnte planmäßig am [Enddatum] beendet werden", hier wird nur das Enddatum extrahiert, während das Startdatum auf "01-01-1901" gesetzt wird

3. Formatierung der Daten

- Gib die Daten im Format "DD-MM-YYYY" zurück, wenn sie vorhanden sind

- Wenn nur das Enddatum bekannt ist, setze für das Startdatum "01-01-1901"

- Wenn kein Datum gefunden wird, setze beide Werte auf "01-01-1901" und "IsPlannedEndDateDetermined" auf "0"

- Wenn ein Enddatum gefunden wird, setze "IsPlannedEndDateDetermined" auf "1"

### Beispiele:

1. Text: der sich vom 24.06. bis 03.07.2024

Denken wir Schritt für Schritt:

- Der Text enthält die Formulierung "vom 24.06. bis 03.07.2024", was auf den Zeitraum der Strahlentherapie hinweist.

- Das geplante Startdatum ist "24.06.2024". Dieses Datum wird direkt aus dem Text extrahiert.

- Das geplante Enddatum ist "03.07.2024". Dieses Datum wird ebenfalls direkt aus dem Text extrahiert.

- Beide Daten werden gemäß den Regeln in das Format "DD-MM-YYYY" umgewandelt.

- Da ein Enddatum vorhanden ist, wird "IsPlannedEndDateDetermined" auf "1" gesetzt.

Antwort:

{

"IsPlannedEndDateDetermined": "1"

"PlannedStartDate": "24-06-2024",

"PlannedEndDate": "03-07-2024"

}

2. Text: der sich vom 14.10.2024 bis zum 23.10.2024

Denken wir Schritt für Schritt:

- Der Text enthält die Formulierung "vom 14.10.2024 bis zum 23.10.2024", was auf den Zeitraum der Strahlentherapie hinweist.

- Das geplante Startdatum ist "14.10.2024". Dieses Datum wird direkt aus dem Text extrahiert.

- Das geplante Enddatum ist "23.10.2024". Dieses Datum wird ebenfalls direkt aus dem Text extrahiert.

- Beide Daten werden gemäß den Regeln in das Format "DD-MM-YYYY" umgewandelt.

- Da ein Enddatum vorhanden ist, wird "IsPlannedEndDateDetermined" auf "1" gesetzt.

Antwort:

{

"IsPlannedEndDateDetermined": "1"

"PlannedStartDate": "14-10-2024",

"PlannedEndDate": "23-10-2024"

}

3. Text: vom 29.04. - 04.05.2021

Denken wir Schritt für Schritt:

- Der Text enthält die Formulierung "vom 29.04. - 04.05.2021", die einen Zeitraum für die Strahlentherapie angibt.

- Das geplante Startdatum ist "29.04.2021". Dieses Datum wird direkt aus dem Text extrahiert.

- Das geplante Enddatum ist "04.05.2021". Dieses Datum wird ebenfalls direkt aus dem Text extrahiert.

- Beide Daten werden gemäß den Regeln in das Format "DD-MM-YYYY" umgewandelt.

- Da ein Enddatum vorhanden ist, wird "IsPlannedEndDateDetermined" auf "1" gesetzt.

Antwort:

{

"IsPlannedEndDateDetermined": "1"

"PlannedStartDate": "29-04-2021",

"PlannedEndDate": "04-05-2021"

}

4. Text: Behandlung vom 01.02.2023 bis 15.03.2023

Denken wir Schritt für Schritt:

- Der Text enthält die Formulierung "vom 01.02.2023 bis 15.03.2023", die einen Zeitraum für die Strahlentherapie angibt.

- Das geplante Startdatum ist "01.02.2023". Dieses Datum wird direkt aus dem Text extrahiert.

- Das geplante Enddatum ist "15.03.2023". Dieses Datum wird ebenfalls direkt aus dem Text extrahiert.

- Beide Daten werden gemäß den Regeln in das Format "DD-MM-YYYY" umgewandelt.

- Da ein Enddatum vorhanden ist, wird "IsPlannedEndDateDetermined" auf "1" gesetzt.

Antwort:

{

"IsPlannedEndDateDetermined": "1"

"PlannedStartDate": "01-02-2023",

"PlannedEndDate": "15-03-2023"

}

5. Text: Risiken und Nebenwirkungen führten wir bis zum 10.03.2021 eine MR-geführte adaptive Bestrahlung der Prostata

Denken wir Schritt für Schritt:

- Der Text enthält die Formulierung "bis zum 10.03.2021", was darauf hinweist, dass dies das Enddatum der Strahlentherapie ist.

- Da kein explizites Startdatum angegeben ist, wird gemäß den Regeln "01-01-1901" als Standardwert für das Startdatum verwendet.

- Das geplante Enddatum ist "10.03.2021". Dieses Datum wird direkt aus dem Text extrahiert.

- Beide Daten werden gemäß den Regeln in das Format "DD-MM-YYYY" umgewandelt.

- Da ein Enddatum vorhanden ist, wird "IsPlannedEndDateDetermined" auf "1" gesetzt.

Antwort:

{

"IsPlannedEndDateDetermined": "0"

"PlannedStartDate": "01-01-1901",

"PlannedEndDate": "10-03-2021"

}

### Antwort Format:

{

"IsPlannedEndDateDetermined": "<'1' oder '0'>",

"PlannedStartDate": "<Datum im Format DD-MM-YYYY oder '01-01-1901'>",

"PlannedEndDate": "<Datum im Format DD-MM-YYYY oder '01-01-1901'>"

}

"""

},

{

"name": "TSG",

"prompt": """

### Query:

Bitte extrahiere die Tumor-Site-Gruppe (TSG), TopographicalCode, MorphologicalCode basierend auf der Diagnose aus dem Text und gib ausschließlich ein einziges JSON-Objekt auf Deutsch zurück. Zusätzliche Texte, Erklärungen oder mehrere JSON-Objekte sind nicht erlaubt."

### Schritte und Regeln:

1. Tumor-Site-Gruppe (TSG)

- Zuordnung anhand der Diagnose zu folgenden Gruppen: Prostata (C61) | Blase (C67) | Gehirn (C71) | Brust (C50) | Gebärmutterhals (C53) | Gebärmutterkörper (C54) | Speiseröhre (C15) | Leber und intrahepatische Gallengänge (C22) | Lunge (C34) | Oropharynx (C10) | Bauchspeicheldrüse (C25) | Prostata (C61) | Mastdarm (C20) | Vagina (C52) | Vulva (C51) | Other

- Beispiel: "Prostatakarzinom" bedeutet "C61"

- Wenn die Diagnose unklar ist oder der Tumorort nicht bestimmt werden kann, gib "Unknown" zurück.

2. TopographischerCode

- Wenn die Diagnose zur Prostata (C61) gehört, gib "C61.9" zurück.

- Andernfalls gib "Unknown" zurück.

3. MorphologicalCode

- Falls die Diagnose folgende Begriffe enthält, gib "8140/3" zurück (Adenokarzinom, NOS): "Prostatakarzinom", "Adenokarzinom" oder "Prostate" oder ähnliche Begriffe für die Prostata.

- Falls die Diagnose "Infiltrierendes duktales Karzinom" enthält, gib "8500/3" zurück.

- Falls die Diagnose "Muzinöses Adenokarzinom" enthält, gib "8480/3" zurück.

- Falls keine eindeutige Angabe vorhanden ist, gib "Unknown" zurück.

- Falls eine andere, nicht aufgeführte Morphologie erwähnt wird, gib "Other" zurück.

### Beispiele:

1. Text: "Diagnose: Intermediäres Risiko Prostata-CA (ED 05/2023)"

Denken wir Schritt für Schritt:

- Der Text enthält die Formulierung "Prostata-CA", was darauf hinweist, dass der Tumor in der Prostata liegt.

- Laut den Regeln entspricht die Prostata der Gruppe "C61".

- Da die Diagnose zur Prostata gehört, ist der Topographische Code "C61.9".

- "Prostata-CA" deutet auf Adenokarzinom, NOS ("8140/3") hin.

Antwort:

{

"TSG": "C61",

"TopographicalCode": "C61.9",

"MorphologicalCode": "8140/3"

}

2. Text: "Diagnose: Intermediate Risk Prostata-Ca (ED 07/21)"

Denken wir Schritt für Schritt:

- Der Text enthält die Formulierung "Prostata-Ca", was auf die Prostata (C61) hinweist.

- Da die Diagnose zur Prostata gehört, ist der Topographische Code "C61.9".

- "Prostata-Ca" deutet auf Adenokarzinom, NOS ("8140/3") hin.

Antwort:

{

"TSG": "C61",

"TopographicalCode": "C61.9",

"MorphologicalCode": "8140/3"

}

3. Text: "Diagnose: High-Risk Prostatakarzinom ED 05/2024"

Denken wir Schritt für Schritt:

- Der Text enthält die Formulierung "Prostatakarzinom", was auf die Prostata (C61) hinweist.

- Da die Diagnose zur Prostata gehört, ist der Topographische Code "C61.9".

- "Prostatakarzinom" deutet auf Adenokarzinom, NOS ("8140/3") hin.

Antwort:

{

"TSG": "C61",

"TopographicalCode": "C61.9",

"MorphologicalCode": "8140/3"

}

4. Text: "Diagnose: Prostatakarzinom high-risk (ED 05/17), PSA-Rezidiv (06/2019)"

Denken wir Schritt für Schritt:

- Der Text enthält die Formulierung "Prostatakarzinom", was auf die Prostata (C61) hinweist.

- Da die Diagnose zur Prostata gehört, ist der Topographische Code "C61.9".

- "Prostatakarzinom" deutet auf Adenokarzinom, NOS ("8140/3") hin.

Antwort:

{

"TSG": "C61",

"TopographicalCode": "C61.9",

"MorphologicalCode": "8140/3"

}

5. Text: "Diagnose: High risk PC (ED 12/2019) Aktuell PSA Anstieg bei R1"

Denken wir Schritt für Schritt:

- "PC" ist eine Abkürzung für "Prostatakarzinom" bedeutet Prostata (C61).

- Da die Diagnose zur Prostata gehört, ist der Topographische Code "C61.9".

- "PC" bezieht sich auf Prostatakarzinom (Adenokarzinom, NOS "8140/3").

Antwort:

{

"TSG": "C61",

"TopographicalCode": "C61.9",

"MorphologicalCode": "8140/3"

}

### Antwort Format:

{

"TSG": "<Eine der angegebenen Gruppen oder 'Unknown'>",

"TopographicalCode": "<C61.9 oder andere anatomische ICD-O-3 Codes oder 'Unknown'>",

"MorphologicalCode": "<8140/3, 8500/3, 8480/3, 'Unknown' oder 'Other'>"

}

"""

},

{

"name": "DiagnoseDate",

"prompt": """

### Query:

Bitte extrahiere die DiagnoseDate basierend auf der Diagnose aus dem Text und gib ausschließlich ein einziges JSON-Objekt auf Deutsch zurück. Zusätzliche Texte, Erklärungen oder mehrere JSON-Objekte sind nicht erlaubt.

Das DiagnoseDate bezieht sich auf das Datum der Erstdiagnose eines Prostatakarzinoms. Es ist das Datum, an dem das Prostatakarzinom zum ersten Mal diagnostiziert wurde. Falls dieses nicht direkt verfügbar ist, wird das nächstbeste relevante Datum gemäß den untenstehenden Regeln extrahiert. Das extrahierte Datum muss exakt im angegebenen Format zurückgegeben werden.

### Schritte und Regeln:

1. Identifiziere das DiagnoseDatum:

- Dieses Datum befindet sich typischerweise im Abschnitt der Diagnose.

- Suche zuerst nach dem Datum der Diagnose eines Prostatakarzinoms.

- Wenn ein solches Datum vorhanden ist, verwende dieses.

2. Suche zusätzlich nach biopsiebezogenen Begriffen: // <-- ergänzt

- Biopsie-bezogene Begriffe umfassen z. B.:

Biopsie, Stanzbiopsie, Fusionsbiopsie, Rebiopsie, Prostatastanzbiopsie, Prostatastanzen.

- Extrahiere das Datum, das in unmittelbarer Nähe zu einem dieser Begriffe steht.

- Falls mehrere Biopsie-bezogene Begriffe gefunden werden, wähle das früheste Datum.

3. Präzisiere das DiagnoseDatum (falls nötig): // <-- ergänzt

- Wenn das DiagnoseDatum aus Schritt 1 unvollständig ist (z. B. nur Monat/Jahr oder nur Jahr), nutze das biopsiebezogene Datum aus Schritt 2 zur Ergänzung der fehlenden Bestandteile.

- Beispiel: „ED 06/21“ + Biopsie am 16.06.2021 → „16-06-2021“

- Beispiel: „ED 2020“ + Biopsie im Januar 2020 → „01-01-2020“

- Wenn das DiagnoseDatum vollständig ist (Tag, Monat, Jahr), wird dieses unverändert übernommen.

4. Falls kein relevantes Datum gefunden werden kann:

- Gib "01-01-1901" zurück.

### Formatierungsregeln für das Datum:

- Wenn das Datum Tag, Monat und Jahr enthält, gib es im Format "DD-MM-YYYY" zurück.

- Wenn das Datum nur Monat und Jahr enthält, gib "01-MM-YYYY" zurück.

- Wenn nur das Jahr vorhanden ist, gib "01-01-YYYY" zurück.

- Falls kein Datum gefunden wird, gib "01-01-1901" zurück.

### Beispiele:

1. Text: Diagnose High risk Prostatakarzinom (ED 06/21), IPSS 16

Aktueller Verlauf

Datum/Zeitraum Befunde

26.01.2021 MR Prostata: V.a. ausgedehntes multifokales Prostatakarzinom mit führender Läsion in der zentralen Zone rechts bis zum Apex in der Medianlinie reichend, Durchmesser 3,7 x 1,3 x 2,7 cm (PI-RADS 5). Multiple weitere Herde zwischen 3 und 7 mm. Möglich Kapselinfiltration anterolateral apexnah rechts. Keine suspekten Lymphknoten. Prostatavolumen 85 ml.

16.06.2021 MRT/TRUS-fusionierte roboterassistierte stereotaktische transperineale Prostatabiopsie (BIOBOT)

Denken wir Schritt für Schritt:

- Der Abschnitt "Diagnose" enthält die Angabe "(ED 06/21)", was auf das Erstdiagnosedatum für das Prostatakarzinom hinweist.

- Das Datum "06/21" enthält nur Monat und Jahr.

- Zusätzlich liegt ein biopsiebezogenes Datum vor: 16.06.2021.

- Laut den neuen Regeln wird das unvollständige Datum mit dem präziseren Biopsiedatum ergänzt.

- Somit ergibt sich ein vollständiges DiagnoseDatum: "16-06-2021".

Antwort:

{

"DiagnoseDate": "01-06-2021"

}

2. Text: Diagnose Unfavorable intermediate risk Prostata-CA (05/2023)

Kurzverlauf

Datum/Zeitraum Befunde

20.04.2023 MR lumbaler Spinalkanal: Knochenmarksödem an der Bodenplatte LWK 2 sowie aneinander liegende Abschlussplatten LWK 1/2. Deswegen Aktivierung des Facettengelenks LWK 4/5 und insbesondere LWK 5/SWK 1.

05/2023 ED Prostata-CA cT2b GS 7b PSA 2,69 ng/ml

Denken wir Schritt für Schritt:

- Der Abschnitt "Diagnose" enthält die Angabe "(05/2023)", was auf das diagnosedatum für das Prostatakarzinom hinweist.

- Gemäß den Regeln wird das erste DiagnoseDatum bevorzugt verwendet.

- Das Datum "05/2023" enthält nur Monat und Jahr.

- Laut Formatierungsregeln wird der erste Tag des Monats angenommen und das Datum als "01-05-2023" formatiert.

Antwort:

{

"DiagnoseDate": "01-05-2023"

}

3. Text: Diagnose: Intermediate risk PC (ED 2020), IPSS 4

Kurzverlauf:

01/2020 TUR-P bei obstruktiven Miktionsbeschwerden -> inzidentell diagnostiziertes PC GS 7a

2020 - 2023: watch and wait mit jetzt wieder steigendem PSA-Wert

08.11.2023 PSMA-PET-MRT: V.a. Prostatakarzinom im verbleibenden Prostataparenchym nach TUR-P. Kein Nachweis von Metastasen. Schwache fokale PSMA-Expression in LWK 3 ohne Korrelat in der MRT, nicht als Metastase zu werten.

Denken wir Schritt für Schritt:

- Der Abschnitt "Diagnose" enthält die Angabe "(ED 2020)", was auf das Erstdiagnosedatum für das Prostatakarzinom hinweist.

- Gemäß den Regeln wird das erste DiagnoseDatum bevorzugt verwendet.

- Das Datum "2020" enthält nur das Jahr.

- Laut Formatierungsregeln wird der erste Tag des Jahres angenommen und das Datum als "01-01-2020" formatiert.

Antwort:

{

"DiagnoseDate": "01-01-2020"

}

4. Text: Diagnose Intermediate Risk Prostatakarzinom

Aktueller Verlauf

Datum/Zeitraum Befunde

14.01.2022 Prostatastanzen

24.01.2022 Reutlingen TuKo: Staging. RT vs. Abwartendes Vorgehen

05.05.2022 GKS: cM0

21.06.2022 CT T/A/B: cM0, kein organüberschreitendes Wachstum, cN0.

28.07.2022 MR-Prostata: Auffälliges Areal rechts Paramedian in der Transitionalzone

Denken wir Schritt für Schritt:

- Der Abschnitt "Diagnose" enthält keine explizite Datumsangabe für das Prostatakarzinom.

- Da kein DiagnoseDatum für Prostatakarzinom gefunden wurde, wird nach biopsie-bezogenen Begriffen gesucht.

- Der Eintrag vom "14.01.2022" enthält das Wort "Prostatastanzen", was auf eine Biopsie hinweist.

- Es gibt keine weiteren Biopsie-bezogenen Begriffe mit einem späteren Datum.

- Gemäß den Regeln wird das späteste Biopsie-Datum verwendet.

- Das Datum "14.01.2022" enthält Tag, Monat und Jahr und wird direkt übernommen.

Antwort:

{

"DiagnoseDate": "14-01-2022"

}

5. Text: Diagnose Intermediate favourable Prostatakarzinom

11/2008 Stanzbioptischer Ausschluss eines Prostatakarzinoms (PSA 1,63)

04/23 TRUS: homogen, Kapsel intakt, Samenblasen unauffällig, Prostata-Volumen 70ccm

03.04.2023 Rebiopsie

Denken wir Schritt für Schritt:

- Der Abschnitt "Diagnose" enthält keine explizite Datumsangabe für die Diagnose des Prostatakarzinoms.

- Da kein DiagnoseDatum für Prostatakarzinom gefunden wurde, wird nach Biopsie-bezogenen Begriffen gesucht.

- Der Text enthält die Begriffe "Stanzbioptischer Ausschluss" (11/2008) und "Rebiopsie" (03.04.2023).

- Gemäß den Regeln wird das früheste Biopsie-Datum verwendet.

- Das Datum "11/2008" enthält nur Monat und Jahr.

- Laut den Formatierungsregeln wird der erste Tag des Monats angenommen, und das Datum wird als "01-11-2008" formatiert.

Antwort:

{

"DiagnoseDate": "01-11-2008"

}

### Antwort Format:

{

"DiagnoseDate": "<Datum im Format DD-MM-YYYY, 01-MM-YYYY, 01-01-YYYY oder '01-01-1901'>"

}

"""

},

{

"name": "Stadium",

"prompt": """

### Query:

Bitte extrahiere die Werte des Tumorstadiums (Stadium) aus dem Text und gib ausschließlich ein einziges JSON-Objekt auf Deutsch zurück. Zusätzliche Texte, Erklärungen oder mehrere JSON-Objekte sind nicht erlaubt:

### Schritte und Regeln:

1. Suche im Text nach Stadium-Werten.

2. Wenn Stadium-Werte vorhanden sind:

- Extrahiere alle gefundenen Werte als Liste.

- Trenne die Werte in der Liste mit einem Komma (,) und setze sie in eckige Klammern [].

3. Wenn keine Informationen zu Stadium gefunden werden, gib eine leere Liste zurück.

### Beispiele:

1. Text: "Stadium: pT3a cN1 cM0."

Denken wir Schritt für Schritt:

- Der Text enthält die Angabe "Stadium: pT3a cN1 cM0".

- Die Stadium-Werte sind 'pT3a', 'cN1' und 'cM0'.

- Wir extrahieren die Werte und geben sie als Liste zurück.

Antwort:

{

"Stadium": ['pT3a', 'cN1', 'cM0']

2. Text: "Stadium: cT3a, cN0, cM0."

Denken wir Schritt für Schritt:

- Der Text enthält die Angabe "Stadium: cT3a, cN0, cM0".

- Die Stadium-Werte sind 'cT3a', 'cN0' und 'cM0'.

- Wir extrahieren die Werte und geben sie als Liste zurück.

Antwort:

{

"Stadium": ['cT3a', 'cN0', 'cM0']

}

3. Text: "Stadium pT2c pN0 (0/24 LK), L0, V0, Pn1, R1."

Denken wir Schritt für Schritt:

- Der Text enthält die Angabe "Stadium pT2c pN0 (0/24 LK), L0, V0, Pn1, R1".

- Die Stadium-Werte sind 'pT2c', 'pN0', 'L0', 'V0', 'Pn1' und 'R1'.

- Wir extrahieren die Werte und geben sie als Liste zurück.

Antwort:

{

"Stadium": ['pT2c', 'pN0', 'L0', 'V0', 'Pn1', 'R1']

}

4. Text: "Stadium: N1 M1 (oss)."

Denken wir Schritt für Schritt:

- Der Text enthält die Angabe "Stadium: N1 M1 (oss)".

- Die Stadium-Werte sind 'N1' und 'M1'.

- Wir extrahieren die Werte und geben sie als Liste zurück.

Antwort:

{

"Stadium": ['N1', 'M1']

}

5. Text: "Keine Angaben zum Stadium im Bericht."

Denken wir Schritt für Schritt:

- Der Text erwähnt keine Informationen zu Stadium.

- Laut den Regeln geben wir eine leere Liste zurück.

Antwort:

{

"Stadium": []

}

### Antwort Format:

{

"Stadium": ['<Liste der extrahierten Stadium-Werte>' oder []]

}

"""

},

{

"name": "pTumorStage",

"prompt": """

### Query:

Bitte extrahiere das pathologische Tumorstadium (pTumor stage) aus der bereitgestellten Liste und gib ausschließlich ein einziges JSON-Objekt zurück. Zusätzliche Texte, Erklärungen oder mehrere JSON-Objekte sind nicht erlaubt.

### Schritte und Regeln:

1. Suche ausschließlich nach einem Eintrag, der mit "pT" beginnt.

2. Der Teil nach "pT" muss exakt einem der folgenden Werte entsprechen: X, 0, 1, 1a, 1b, 1c, 2, 2a, 2b, 2c, 3, 3a, 3b, 4

- Die Groß-/Kleinschreibung ist zu beachten.

3. Es dürfen **keine klinischen Angaben (z. B. 'cT...', 'cN...', 'cM...')** oder andere pathologische Angaben (z. B. 'pN...') verwendet werden.

4. Der zurückgegebene Wert muss **direkt und vollständig** aus der Liste stammen.

5. Wenn kein gültiger `pT`-Wert vorhanden ist, gib `"Unknown"` zurück.

### Beispiele:

1. Liste: ['cT3', 'cN1', 'cM0']

Denken wir Schritt für Schritt:

- Die Liste enthält 'cT3', 'cN1', 'cM0'.

- Es gibt keine pathologischen Stadien ('p...'), daher setzen wir alle p-Stadien auf "Unknown".

Antwort:

{

"pTumorStage": "Unknown"

}

2. Liste: ['cT3a', 'cN0', 'cM0']

Denken wir Schritt für Schritt:

- Die Liste enthält 'cT3a', 'cN0', 'cM0'.

- Es gibt keine pathologischen Stadien ('p...'), daher setzen wir alle p-Stadien auf "Unknown".

Antwort:

{

"pTumorStage": "Unknown"

}

3. Liste: ['cT2a', 'cN0', 'cM0']

Denken wir Schritt für Schritt:

- Die Liste enthält 'cT2a', 'cN0', 'cM0'.

- Es gibt keine pathologischen Stadien ('p...'), daher setzen wir alle p-Stadien auf "Unknown".

Antwort:

{

"pTumorStage": "Unknown"

}

4. Liste: ['pT2c', 'pN0 (0/20)', 'L0', 'V0']

Denken wir Schritt für Schritt:

- Die Liste enthält 'pT2c', 'pN0 (0/20)', 'L0', 'V0'.

- 'pT2c' beginnt mit 'p' und gehört zur Kategorie "T2c", daher ist das pTumor stage "T2c".

Antwort:

{

"pTumorStage": "T2c"

}

5. Liste: ["N1", "M1"]

Denken wir Schritt für Schritt:

- Die Liste enthält "N1" und "M1".

- Es gibt keine pathologischen Stadien ('p...'), daher setzen wir alle p-Stadien auf "Unknown".

Antwort:

{

"pTumorStage": "Unknown"

}

### Antwort Format:

{

"pTumorStage": "<Einer der Werte: 'TX', 'T0', 'T1', 'T1a', 'T1b', 'T1c', 'T2', 'T2a', 'T2b', 'T2c', 'T3', 'T3a', 'T3b', 'T4', 'Unknown'>"

}

Liste:

"""

},

{

"name": "pNodalStage",

"prompt": """

### Query:

Bitte extrahiere das pathologische Lymphknotenstadium (pNodal stage) aus der bereitgestellten Liste und gib ausschließlich ein einziges JSON-Objekt zurück. Zusätzliche Texte, Erklärungen oder mehrere JSON-Objekte sind nicht erlaubt.

### Schritte und Regeln:

1. Suche ausschließlich nach einem Eintrag in der Liste, der mit **"pN"** beginnt.

2. Der Teil nach "pN" muss exakt einem der folgenden Werte entsprechen: **X**, **0**, **1** (also: **pNX**, **pN0**, **pN1**).

- Groß-/Kleinschreibung ist zu beachten.

3. Es dürfen **keine klinischen Angaben** wie z. B. **'cN...'**, **'cT...'**, **'cM...'** verwendet werden.

4. Es dürfen außerdem **keine pathologischen T- oder M-Stadien** (z. B. **'pT...'**, **'pM...'**) als Ersatz genutzt werden.

5. Der zurückgegebene Wert muss **direkt und exakt** aus der Liste stammen.

6. Wenn **kein gültiger `pN`-Wert** vorhanden ist, gib `"Unknown"` zurück.

### Beispiele:

1. Liste: ['cT3', 'cN1', 'cM0']

Denken wir Schritt für Schritt:

- Die Liste enthält 'cT3', 'cN1', 'cM0'.

- Es gibt keine pathologischen Stadien ('p...'), daher setzen wir das pNodal stage auf "Unknown".

Antwort:

{

"pNodalStage": "Unknown"

}

2. Liste: ['cT3a', 'cN0', 'cM0']

Denken wir Schritt für Schritt:

- Die Liste enthält 'cT3a', 'cN0', 'cM0'.

- Es gibt keine pathologischen Stadien ('p...'), daher setzen wir das pNodal stage auf "Unknown".

Antwort:

{

"pNodalStage": "Unknown"

}

3. Liste: ['cT2a', 'cN0', 'cM0']

Denken wir Schritt für Schritt:

- Die Liste enthält 'cT2a', 'cN0', 'cM0'.

- Es gibt keine pathologischen Stadien ('p...'), daher setzen wir das pNodal stage auf "Unknown".

Antwort:

{

"pNodalStage": "Unknown"

}

4. Liste: ['pT2c', 'pN0 (0/20)', 'L0', 'V0']

Denken wir Schritt für Schritt:

- Die Liste enthält 'pT2c', 'pN0 (0/20)', 'L0', 'V0'.

- 'pN0 (0/20)' beginnt mit 'p' und gehört zur Kategorie "N0", daher ist das pNodal stage "N0".

Antwort:

{

"pNodalStage": "N0"

}

5. Liste: ["N1", "M1"]

Denken wir Schritt für Schritt:

- Die Liste enthält "N1" und "M1".

- Es gibt keine pathologischen Stadien ('p...'), daher setzen wir das pNodal stage auf "Unknown".

Antwort:

{

"pNodalStage": "Unknown"

}

### Antwort Format:

{

"pNodalStage": "<Einer der Werte: 'NX', 'N0', 'N1', 'Unknown'>"

}

Liste:

"""

},

{

"name": "pMetastasisStage",

"prompt": """

### Query:

Bitte extrahiere das pathologische Metastasenstadium (pMetastasis stage) aus der bereitgestellten Liste und gib ausschließlich ein einziges JSON-Objekt zurück. Zusätzliche Texte, Erklärungen oder mehrere JSON-Objekte sind nicht erlaubt.

### Schritte und Regeln:

1. Suche ausschließlich nach einem Eintrag in der Liste, der mit **"pM"** beginnt.

2. Der Teil nach "pM" muss exakt einem der folgenden Werte entsprechen: **0** oder **1** (also: **pM0** oder **pM1**).

- Groß-/Kleinschreibung ist zu beachten.

3. Es dürfen **keine klinischen Angaben** wie **'cM...'**, **'cT...'**, **'cN...'** verwendet werden.

4. Es dürfen außerdem **keine pathologischen T- oder N-Stadien** (z. B. **'pT...'**, **'pN...'**) als Ersatz verwendet werden.

5. Der zurückgegebene Wert muss **direkt und vollständig** aus der Liste stammen.

6. Wenn **kein gültiger `pM`-Wert** in der Liste enthalten ist, gib `"Unknown"` zurück.

### Beispiele:

1. Liste: ['cT3', 'cN1', 'cM0']

Denken wir Schritt für Schritt:

- Die Liste enthält 'cT3', 'cN1', 'cM0'.

- Es gibt keine pathologischen Stadien ('p...'), daher setzen wir das pMetastasis stage auf "Unknown".

Antwort:

{

"pMetastasisStage": "Unknown"

}

2. Liste: ['cT3a', 'cN0', 'cM0']

Denken wir Schritt für Schritt:

- Die Liste enthält 'cT3a', 'cN0', 'cM0'.

- Es gibt keine pathologischen Stadien ('p...'), daher setzen wir das pMetastasis stage auf "Unknown".

Antwort:

{

"pMetastasisStage": "Unknown"

}

3. Liste: ['pT2c', 'pN0 (0/20)', 'L0', 'V0']

Denken wir Schritt für Schritt:

- Die Liste enthält 'pT2c', 'pN0 (0/20)', 'L0', 'V0'.

- Es gibt keinen Eintrag, der mit "p" beginnt und zu den Kategorien M0 oder M1 gehört.

- Daher setzen wir das pMetastasis stage auf "Unknown".

Antwort:

{

"pMetastasisStage": "Unknown"

}

4. Liste: ['pT1', 'pN1', 'pM1']

Denken wir Schritt für Schritt:

- Die Liste enthält 'pT1', 'pN1', 'pM1'.

- 'pM1' beginnt mit 'p' und gehört zur Kategorie "M1", daher ist das pMetastasis stage "M1".

Antwort:

{

"pMetastasisStage": "M1"

}

5. Liste: ["N1", "M1"]

Denken wir Schritt für Schritt:

- Die Liste enthält "N1" und "M1".

- Es gibt keine pathologischen Stadien ('p...'), daher setzen wir das pMetastasis stage auf "Unknown".

Antwort:

{

"pMetastasisStage": "Unknown"

}

### Antwort Format:

{

"pMetastasisStage": "<Einer der Werte: 'M0', 'M1', 'Unknown'>"

}

Liste:

"""

},

{

"name": "cTumorStage",

"prompt": """

### Query:

Bitte extrahiere das klinische Tumorstadium (cTumor stage) aus der bereitgestellten Liste und gib ausschließlich ein einziges JSON-Objekt zurück. Zusätzliche Texte, Erklärungen oder mehrere JSON-Objekte sind nicht erlaubt.

### Schritte und Regeln:

1. Suche ausschließlich nach einem Eintrag in der Liste, der mit **"cT"** beginnt.

2. Der Teil nach "cT" muss exakt einem der folgenden Werte entsprechen: **X**, **0**, **1**, **1a**, **1b**, **1c**, **2**, **2a**, **2b**, **2c**, **3**, **3a**, **3b**, **4**.

- Also: gültige Stadien sind "cTX", "cT0", "cT1", ..., "cT4".

- Groß-/Kleinschreibung ist zu beachten.

3. Es dürfen **keine pathologischen Angaben** wie **'pT...'**, **'pN...'**, **'pM...'** verwendet werden.

4. Es dürfen außerdem **keine klinischen Angaben außerhalb des Tumorstadiums** (z. B. **'cN...'**, **'cM...'**) übernommen

### Beispiele:

1. Liste: ['cT3', 'cN1', 'cM0']

Denken wir Schritt für Schritt:

- Die Liste enthält 'cT3', 'cN1', 'cM0'.

- 'cT3' beginnt mit 'c' und gehört zur Kategorie "T3", daher ist das cTumor stage "T3".

Antwort:

{

"cTumorStage": "T3"

}

2. Liste: ['cT3a', 'cN0', 'cM0']

Denken wir Schritt für Schritt:

- Die Liste enthält 'cT3a', 'cN0', 'cM0'.

- 'cT3a' beginnt mit 'c' und gehört zur Kategorie "T3a", daher ist das cTumor stage "T3a".

Antwort:

{

"cTumorStage": "T3a"

}

3. Liste: ['cT2a', 'cN0', 'cM0']

Denken wir Schritt für Schritt:

- Die Liste enthält 'cT2a', 'cN0', 'cM0'.

- 'cT2a' beginnt mit 'c' und gehört zur Kategorie "T2a", daher ist das cTumor stage "T2a".

Antwort:

{

"cTumorStage": "T2a"

}

4. Liste: ['pT2c', 'pN0 (0/20)', 'L0', 'V0']

Denken wir Schritt für Schritt:

- Die Liste enthält 'pT2c', 'pN0 (0/20)', 'L0', 'V0'.

- Es gibt keinen Eintrag, der mit "c" beginnt und zu den Kategorien TX, T0, T1, T2, ..., T4 gehört.

- Daher setzen wir das cTumor stage auf "Unknown".

Antwort:

{

"cTumorStage": "Unknown"

}

5. Liste: ["N1", "M1"]

Denken wir Schritt für Schritt:

- Die Liste enthält "N1" und "M1".

- Es gibt keinen Eintrag, der mit "c" beginnt und zu den Kategorien TX, T0, T1, T2, ..., T4 gehört.

- Daher setzen wir das cTumor stage auf "Unknown".

Antwort:

{

"cTumorStage": "Unknown"

}

### Antwort Format:

{

"cTumorStage": "<Einer der Werte: 'TX', 'T0', 'T1', 'T1a', 'T1b', 'T1c', 'T2', 'T2a', 'T2b', 'T2c', 'T3', 'T3a', 'T3b', 'T4', 'Unknown'>"

}

Liste:

"""

},

{

"name": "cNodalStage",

"prompt": """

### Query:

Bitte extrahiere das klinische Lymphknotenstadium (cNodal stage) aus der bereitgestellten Liste und gib ausschließlich ein einziges JSON-Objekt zurück. Zusätzliche Texte, Erklärungen oder mehrere JSON-Objekte sind nicht erlaubt.

### Schritte und Regeln:

1. Suche ausschließlich nach einem Eintrag in der Liste, der mit **"cN"** beginnt.

2. Der Teil nach "cN" muss exakt einem der folgenden Werte entsprechen: **X**, **0**, **1** (also: **cNX**, **cN0**, **cN1**).

- Groß-/Kleinschreibung ist zu beachten.

3. Es dürfen **keine pathologischen Angaben** wie **'pN...'**, **'pT...'**, **'pM...'** verwendet werden.

4. Es dürfen außerdem **keine klinischen Angaben außerhalb des Lymphknotenstadiums** (z. B. **'cT...'**, **'cM...'**) übernommen werden.

5. Der zurückgegebene Wert muss **direkt und exakt** aus der Liste stammen.

6. Wenn **kein gültiger `cN`-Wert** vorhanden ist, gib `"Unknown"` zurück.

### Beispiele:

1. Liste: ['cT3', 'cN1', 'cM0']

Denken wir Schritt für Schritt:

- Die Liste enthält 'cT3', 'cN1', 'cM0'.

- 'cN1' beginnt mit 'c' und gehört zur Kategorie "N1", daher ist das cNodal stage "N1".

Antwort:

{

"cNodalStage": "N1"

}

2. Liste: ['cT3a', 'cN0', 'cM0']

Denken wir Schritt für Schritt:

- Die Liste enthält 'cT3a', 'cN0', 'cM0'.

- 'cN0' beginnt mit 'c' und gehört zur Kategorie "N0", daher ist das cNodal stage "N0".

Antwort:

{

"cNodalStage": "N0"

}

3. Liste: ['cT2a', 'cN0', 'cM0']

Denken wir Schritt für Schritt:

- Die Liste enthält 'cT2a', 'cN0', 'cM0'.

- 'cN0' beginnt mit 'c' und gehört zur Kategorie "N0", daher ist das cNodal stage "N0".

Antwort:

{

"cNodalStage": "N0"

}

4. Liste: ['pT2c', 'pN0 (0/20)', 'L0', 'V0']

Denken wir Schritt für Schritt:

- Die Liste enthält 'pT2c', 'pN0 (0/20)', 'L0', 'V0'.

- Es gibt keinen Eintrag, der mit "c" beginnt und zu den Kategorien NX, N0 oder N1 gehört.

- Daher setzen wir das cNodal stage auf "Unknown".

Antwort:

{

"cNodalStage": "Unknown"

}

5. Liste: ["N1", "M1"]

Denken wir Schritt für Schritt:

- Die Liste enthält "N1" und "M1".

- Es gibt keinen Eintrag, der mit "c" beginnt und zu den Kategorien NX, N0 oder N1 gehört.

- Daher setzen wir das cNodal stage auf "Unknown".

Antwort:

{

"cNodalStage": "Unknown"

}

### Antwort Format:

{

"cNodalStage": "<Einer der Werte: 'NX', 'N0', 'N1', 'Unknown'>"

}

Liste:

"""

},

{

"name": "cMetastasisStage",

"prompt": """

### Query:

Bitte extrahiere das klinische Metastasenstadium (cMetastasis stage) aus der bereitgestellten Liste und gib ausschließlich ein einziges JSON-Objekt zurück. Zusätzliche Texte, Erklärungen oder mehrere JSON-Objekte sind nicht erlaubt.

### Schritte und Regeln:

1. Suche ausschließlich nach einem Eintrag in der Liste, der mit **"cM"** beginnt.

2. Der Teil nach "cM" muss exakt einem der folgenden Werte entsprechen: **0**, **1**, **1a**, **1b**, **1c**.

- Gültige Werte sind also: "cM0", "cM1", "cM1a", "cM1b", "cM1c".

- Der Wert **"cMx"** ist **nicht gültig** und darf **nicht** extrahiert werden.

- Groß-/Kleinschreibung ist zu beachten.

3. Es dürfen **keine pathologischen Angaben** wie **'pM...'**, **'pT...'**, **'pN...'** verwendet werden.

4. Es dürfen außerdem **keine klinischen Angaben außerhalb des Metastasenstadiums** (z. B. **'cT...'**, **'cN...'**) übernommen werden.

5. Der zurückgegebene Wert muss **direkt und exakt** aus der Liste stammen.

6. Wenn **kein gültiger `cM`-Wert** vorhanden ist, gib `"Unknown"` zurück.

### Beispiele:

1. Liste: ['cT3', 'cN1', 'cM0']

Denken wir Schritt für Schritt:

- Die Liste enthält 'cT3', 'cN1', 'cM0'.

- 'cM0' beginnt mit 'c' und gehört zur Kategorie "M0", daher ist das cMetastasis stage "M0".

Antwort:

{

"cMetastasisStage": "M0"

}

2. Liste: ['cT3a', 'cN0', 'cM0']

Denken wir Schritt für Schritt:

- Die Liste enthält 'cT3a', 'cN0', 'cM0'.

- 'cM0' beginnt mit 'c' und gehört zur Kategorie "M0", daher ist das cMetastasis stage "M0".

Antwort:

{

"cMetastasisStage": "M0"

}

3. Liste: ['cT2a', 'cN0', 'cM0']

Denken wir Schritt für Schritt:

- Die Liste enthält 'cT2a', 'cN0', 'cM0'.

- 'cM0' beginnt mit 'c' und gehört zur Kategorie "M0", daher ist das cMetastasis stage "M0".

Antwort:

{

"cMetastasisStage": "M0"

}

4. Liste: ['cMx', 'pT2c', 'pN0 (0/20)', 'L0', 'V0']

Denken wir Schritt für Schritt:

- Die Liste enthält 'cMx', 'pT2c', 'pN0 (0/20)', 'L0', 'V0'.

- Es gibt keinen Eintrag, der mit "c" beginnt und zu den Kategorien M0, M1, M1a, M1b oder M1c gehört.

- 'Mx' ist nicht zu den Kategorien M0, M1, M1a, M1b oder M1c gehört.

- Daher setzen wir das cMetastasis stage auf "Unknown".

Antwort:

{

"cMetastasisStage": "Unknown"

}

5. Liste: ["N1", "M1"]

Denken wir Schritt für Schritt:

- Die Liste enthält "N1" und "M1".

- Es gibt keinen Eintrag, der mit "c" beginnt und zu den Kategorien M0, M1, M1a, M1b oder M1c gehört.

- Daher setzen wir das cMetastasis stage auf "Unknown".

Antwort:

{

"cMetastasisStage": "Unknown"

}

### Antwort Format:

{

"cMetastasisStage": "<Einer der Werte: 'M0', 'M1', 'M1a', 'M1b', 'M1c', 'Unknown'>"

}

Liste:

"""

},

{

"name": "GleasonScore",

"prompt": """

### Query:

Bitte extrahiere den Gleason-Score (Gleason Score) aus dem Text und gib **ausschließlich ein einziges JSON-Objekt** zurück. Zusätzliche Texte, Erklärungen oder mehrere JSON-Objekte sind **nicht erlaubt**:

Der Gleason-Score:

- Befindet sich typischerweise in Abschnitten mit "Stadium" oder "Histologie".

- Wird als eine Zahl oder in einem Additionsformat ("Zahl+Zahl=Zahl") angegeben.

### Schritte und Regeln:

1. Suche zuerst im Abschnitt "Stadium" nach einem Gleason-Score.

2. Falls im Abschnitt "Stadium" kein Gleason-Score gefunden wird, suche stattdessen im Abschnitt "Histologie".

3. Gleason-Score befindet sich typischerweise hinter Begriffen wie "Gleason-Score", "Gleason Score", "Gleason", "Gl-Sc", "Gl.", "GS oder "Stanzen Gleason".

4. Falls ein Additionsformat ("Zahl+Zahl=Zahl") direkt hinter diesen Begriffen erscheint, extrahiere das vollständige Additionsformat.

5. Falls kein Additionsformat vorhanden ist, aber eine einzelne Zahl hinter den oben genannten Begriffen steht, extrahiere diese Zahl.

6. Falls mehrere Gleason-Scores mit unterschiedlichen Daten vorhanden sind, wähle den Score mit dem neuesten Datum.

7. Falls im Abschnitt "Stadium" und "Histologie" kein Gleason-Score gefunden wird, gib "Unknown" für GleasonScore zurück und setze isGleasonDetermined auf "0".

8. Falls ein Gleason-Score gefunden wird, setze isGleasonDetermined auf "1".

### Beispiele:

1. Text: Stadium pT1c cN0 cM0

Histologie 12/2021 Stanzbiopsie der Prostata: Adenokarzinom in 5 von 11 Stanzen, Gleason 7a, ISUP 2, Infiltration 10-70%, vereinzelt Pn1

Denken wir Schritt für Schritt:

- Der Abschnitt "Stadium" enthält keinen Gleason-Score.

- Der Abschnitt "Histologie" enthält einen Gleason-Score: "Gleason 7a".

- Da es keinen Additionsformat gibt, extrahieren wir die einzelne Zahl "7a".

- Setze `isGleasonDetermined` auf "1".

Antwort:

{

"isGleasonDetermined": "1",

"GleasonScore": "7a"

}

2. Text: Stadium: Min. cT2a, Gleason-Score 7a (3+4)

Histologie: Azinäres Prostataadenokarzinom, 6/18 Stanzen positiv (alle von rechts)

Denken wir Schritt für Schritt:

- Der Abschnitt "Stadium" enthält einen Gleason-Score: "7a (3+4)".

- Da der Gleason-Score im Additionsformat ("3+4") vorliegt, extrahieren wir diesen Wert.

- Setze `isGleasonDetermined` auf "1".

Antwort:

{

"isGleasonDetermined": "1",

"GleasonScore": "7a (3+4)"

}

3. Text: Stadium cT1c cN0 cM0 Gleason 7b

Histologie Prostatakarzinom in 4/15: links medial und mediobasal Gleason 6 in 10 bzw. 30 % des Gewebes. links basal Gleason 7b in 80% des Gewebes, Zielbiopsie links Gleason 7a in 80% des Gewebes. Gesamtgleasonpattern 3 (65%) 4 (35%), Gesamt Score 7

Denken wir Schritt für Schritt:

- Der Abschnitt "Stadium" enthält einen Gleason-Score: "Gleason 7b".

- Da ein Wert im Stadium-Bereich gefunden wurde, ignorieren wir den Histologie-Abschnitt.

- Setze `isGleasonDetermined` auf "1".

Antwort:

{

"isGleasonDetermined": "1",

"GleasonScore": "7b"

}

4. Text: Stadium: cT2c cN0 cM0

Histologie: 09/22: intermediate risk, GS 7a rechte TZ, GS 6 linke PZ

09/23: Upgrading, unfavorable intermediate risk Prostatakarzinom (6/6 Stanzen rechts pos. In TZ bis 45% mit GS 7b = 4+3, tertiär 5) Pn0

Denken wir Schritt für Schritt:

- Der Abschnitt "Stadium" enthält keinen Gleason-Score.

- Der Abschnitt "Histologie" enthält zwei Gleason-Scores mit Datumsangaben: "GS 7a" (09/22) und "GS 7b = 4+3" (09/23).

- Da mehrere Werte existieren, wählen wir das neueste Datum: "09/23".

- Der Score liegt im Additionsformat "4+3", daher extrahieren wir "7b = 4+3".

- Setze `isGleasonDetermined` auf "1".

Antwort:

{

"isGleasonDetermined": "1",

"GleasonScore": "7b = 4+3"

}

5. Text: Stadium: pT3b pN1(1/17) L1 V1 Pn1 R1

Histologie: Stanze: 10/12 Stanzen pos. Bis 100% GS 9 ISUP 5

Adenokarzinom der Prostata, GS 9, ISUP 5, bds. Infiltration der Samenblasen, des angrenzenden extraprostatischen Fettgewebe Bindegewebes dorsal, dorsolateral bds. sowie Perineuralscheideninfiltration, Lymphe und Hämangiosis. Randbildend im Bereich der Samenblase links über ein Areal von 0,3 cm sowie dorsal rechts über ein Areal von 0,2 cm und Blase rechts im Bereich der Samenblase. Im Übrigen fibroadenomatöse Hyperplasie.

Denken wir Schritt für Schritt:

- Der Abschnitt "Stadium" enthält keinen Gleason-Score.

- Der Abschnitt "Histologie" enthält einen Gleason-Score: "GS 9".

- Da kein Additionsformat existiert, extrahieren wir die einzelne Zahl "9".

- Setze `isGleasonDetermined` auf "1".

Antwort:

{

"isGleasonDetermined": "1",

"GleasonScore": "9"

}

### Antwort Format:

{

"isGleasonDetermined": "<'1' oder '0'>",

"GleasonScore": "<Wert im Format 'Addition', 'Zahl' oder 'Unknown'>"

}

"""

},

{

"name": "GleasonScoreDate",

"prompt": """

### Query:

Bitte extrahiere das Datum der Biopsie (z. B. Stanzbiopsie, Fusionsbiopsie, Rebiopsie, MRT-Fusionsbiopsie, Prostatastanzbiopsie) oder, falls kein solches Datum gefunden wird, das relevanteste Datum gemäß den untenstehenden Regeln. Gib ausschließlich ein einziges JSON-Objekt zurück. Zusätzliche Texte, Erklärungen oder mehrere JSON-Objekte sind nicht erlaubt.

### Schritte und Regeln:

1. Suche im Abschnitt "Verlauf" nach Begriffen, die sich auf eine Biopsie beziehen, insbesondere "Stanzbiopsie", "Fusionsbiopsie", "MRT-Fusionsbiopsie" "Rebiopsie" oder "Prostatastanzbiopsie".

2. Extrahiere das Datum, das in der Nähe dieser Begriffe steht (vor oder nach dem Begriff).

3. Falls mehrere Biopsie-bezogene Begriffe mit unterschiedlichen Daten vorhanden sind, wähle das neueste Datum aus.

4. Falls keine Biopsie-bezogenen Begriffe gefunden werden, suche stattdessen nach den Begriffen "Prostatastanzen", "Prostatavesikulektomie", "Prostatovesikulektomie" oder "Prostatektomie" und extrahiere das entsprechende Datum.

5. Falls keine der oben genannten Begriffe gefunden werden, suche nach einem Datum im Abschnitt "Diagnose".

6. Falls auch im Abschnitt "Diagnose" kein Datum gefunden wird, suche nach einem Datum in der Nähe des Gleason Scores im Abschnitt "Histologie".

7. Falls in all diesen Schritten kein Datum gefunden wird, gib "01-01-1901" zurück.

### Formatierungsregeln für das Datum:

- Wenn das Datum Tag, Monat und Jahr enthält, gib es im Format "DD-MM-YYYY" zurück.

- Wenn das Datum nur Monat und Jahr enthält, gib "01-MM-YYYY" zurück.

- Wenn nur das Jahr vorhanden ist, gib "01-01-YYYY" zurück.

- Falls kein Datum gefunden wird, gib "01-01-1901" zurück.

### Beispiele:

1. Text: Diagnose High risk Prostatakarzinom (ED 06/21), IPSS 16

Aktueller Verlauf

Datum/Zeitraum Befunde

26.01.2021 MR Prostata: V.a. ausgedehntes multifokales Prostatakarzinom mit führender Läsion in der zentralen Zone rechts bis zum Apex in der Medianlinie reichend, Durchmesser 3,7 x 1,3 x 2,7 cm (PI-RADS 5). Multiple weitere Herde zwischen 3 und 7 mm. Möglich Kapselinfiltration anterolateral apexnah rechts. Keine suspekten Lymphknoten. Prostatavolumen 85 ml.

16.06.2021 MRT/TRUS-fusionierte roboterassistierte stereotaktische transperineale Prostatabiopsie (BIOBOT)

Denken wir Schritt für Schritt:

- Der Text enthält den Abschnitt "Verlauf", in dem der Begriff "Prostatabiopsie" vorkommt.

- Das Datum "16.06.2021" steht in direkter Nähe zu diesem Begriff.

- Nach Regel 2 wird das neueste Datum einer Biopsie gewählt, falls mehrere vorhanden sind. Hier gibt es nur eines.

- Konvertiere das Datum ins Format "DD-MM-YYYY": "16-06-2021".

Antwort:

{

"GleasonScoreDate": "16-06-2021"

}

2. Text: Diagnose Intermediate risk Prostatakarzinom

Aktueller Verlauf

Datum/Zeitraum Befunde

07/2021 MRT Prostata: signalarme Areale peripher in der Pzpl und Pzpm bds, PIRADS 4, links TZa und TZp sind unspezifisch

10/2021 Prostatastanzbiopsie; GS 6 low risk

active surveillance

06.10.2022 Prostatastanzbiopsie

Denken wir Schritt für Schritt:

- Der Abschnitt "Verlauf" enthält die Begriffe "Prostatastanzbiopsie".

- Die relevanten Daten in der Nähe dieser Begriffe sind "10/2021" und "06.10.2022".

- Nach Regel 2 wird das neueste Datum gewählt, also "06.10.2022".

- Konvertiere das Datum ins Format "DD-MM-YYYY": "06-10-2022".

Antwort:

{

"GleasonScoreDate": "06-10-2022"

}

3. Text: Diagnose Intermediate Risk Prostatakarzinom

Aktueller Verlauf

Datum/Zeitraum Befunde

14.01.2022 Prostatastanzen

24.01.2022 Reutlingen TuKo: Staging. RT vs. Abwartendes Vorgehen

05.05.2022 GKS: cM0

21.06.2022 CT T/A/B: cM0, kein organüberschreitendes Wachstum, cN0.

28.07.2022 MR-Prostata: Auffälliges Areal rechts Paramedian in der Transitionalzone

Denken wir Schritt für Schritt:

- Der Abschnitt "Verlauf" enthält den Begriff "Prostatastanzen".

- Das dazugehörige Datum ist "14.01.2022".

- Da keine Biopsie-bezogenen Begriffe gefunden wurden, greift Regel 3 und wählt das Datum in der Nähe von "Prostatastanzen".

- Konvertiere das Datum ins Format "DD-MM-YYYY": "14-01-2022".

Antwort:

{

"GleasonScoreDate": "14-01-2022"

}

4. Text: Diagnose Unfavorable intermediate risk Prostata-CA (ED 05/2023)

Kurzverlauf

Datum/Zeitraum Befunde

20.04.2023 MR lumbaler Spinalkanal: Knochenmarksödem an der Bodenplatte LWK 2 sowie aneinander liegende Abschlussplatten LWK 1/2. Deswegen Aktivierung des Facettengelenks LWK 4/5 und insbesondere LWK 5/SWK 1.

05/2023 ED Prostata-CA cT2b GS 7b PSA 2,69 ng/ml

19.06.2023 MR Prostata: Herdbefund an der Prostata links mit langstreckigem Kontakt zur Kapsel und Diffusionsrestriktion bei gesichertem Karzinom (BI-RADS 5), die Ausdehnung in der Diffusionswichtung beträgt 12 x 10 mm, in der Kontrastmitteldynamik lässt sich der Befund mit bis zu 20 x 19 mm ausmessen. Langstreckige Kapselkontakt aber kein Nachweis eines kapselüberschreitenden Wachstums, keine pathologischen Lymphknoten im Untersuchungsgebiet. Degenerationen an der Hüfte links mit Ossikel angrenzend an den Trochanter minor, ansonsten regelrechtes Knochenmarksignal.

06/2023 Vermehrte Miktionsbeschwerden unter Pausierung Tamsulosin, Sonographie: kein pathologischer Restharn (15ml)

21.06.2023 GKS: Kein Nachweis einer ossären Metastasierung. Typisch degenerative Veränderungen.

Denken wir Schritt für Schritt:

- Der Abschnitt "Verlauf" enthält keinen expliziten Biopsie-bezogenen Begriff.

- Es gibt jedoch eine Diagnose mit einem Datum: "ED 05/2023".

- Nach Regel 4 wird das Diagnose-Datum verwendet.

- Das Datum enthält nur Monat und Jahr, daher konvertiere es zu "01-05-2023".

Antwort:

{

"GleasonScoreDate": "01-05-2023"

}

5. Text: Diagnose: Intermediate risk PC (ED 2020), IPSS 4

Kurzverlauf:

01/2020 TUR-P bei obstruktiven Miktionsbeschwerden -> inzidentell diagnostiziertes PC GS 7a

2020 - 2023: watch and wait mit jetzt wieder steigendem PSA-Wert

08.11.2023 PSMA-PET-MRT: V.a. Prostatakarzinom im verbleibenden Prostataparenchym nach TUR-P. Kein Nachweis von Metastasen. Schwache fokale PSMA-Expression in LWK 3 ohne Korrelat in der MRT, nicht als Metastase zu werten.

Denken wir Schritt für Schritt:

- Es gibt keine expliziten Biopsie-bezogenen Begriffe.

- Es gibt jedoch eine Diagnose mit dem Datum "ED 2020".

- Nach Regel 4 wird dieses Datum verwendet.

- Das Datum enthält nur das Jahr, daher konvertiere es zu "01-01-2020".

Antwort:

{

"GleasonScoreDate": "01-01-2020"

}

### Antwort Format:

{

"GleasonScoreDate": "<Datum im Format DD-MM-YYYY oder '01-01-1901'>"

}

"""

},

{

"name": "GleasonScoreSeperate",

"prompt": """

### Query:

Bitte ordne die folgenden Gleason-Score-Werte (Gleason Score) den jeweiligen Kategorien zu und gib ausschließlich ein einziges JSON-Objekt zurück. Zusätzliche Texte, Erklärungen oder mehrere JSON-Objekte sind nicht erlaubt.

Die zu extrahierenden Werte und deren mögliche Ausprägungen:

1. Primary Gleason pattern: Werte können 1, 2, 3, 4, 5 oder "Unknown" sein.

2. Secondary Gleason pattern: Werte können 1, 2, 3, 4, 5 oder "Unknown" sein.

3. Total Gleason score: Eine Zahl, z. B. 6, 7, 8, 9, 10 oder "Unknown".

### Schritte und Regeln:

1. Wenn der Wert ein Additionsformat wie "Zahl+Zahl=Zahl" ist:

- Die erste Zahl (vor dem "+") ist der Primary Gleason pattern.

- Die zweite Zahl (nach dem "+") ist der Secondary Gleason pattern.

- Die Zahl nach dem "=" ist der Total Gleason score.

2. Wenn der Wert eine einzelne Zahl mit Buchstaben ist (z. B. "7a", "7b"):

- Verwende bekannte Zuordnungen:

- "7a" bedeutet Primary: 3, Secondary: 4, Total: 7.

- "7b" bedeutet Primary: 4, Secondary: 3, Total: 7.

3. Wenn der Wert eine reine Zahl ohne Buchstaben ist (z. B. "6","7", "8", "9", "10"):

- Bestimme den Total Gleason score direkt aus der Zahl.

- Setze Primary Gleason pattern und Secondary Gleason pattern auf "Unknown".

4. Wenn kein gültiger Wert vorliegt oder die Angaben unklar sind, setze alle Werte auf "Unknown".

### Beispiele:

1. Gleason-Score: "3+4=7a"

Denken wir Schritt für Schritt:

- Der Wert ist im Additionsformat "3+4=7a".

- Die erste Zahl "3" ist der Primary Gleason pattern.

- Die zweite Zahl "4" ist der Secondary Gleason pattern.

- Die Zahl nach dem "=" ist "7", das der Total Gleason score entspricht.

Antwort:

{

"PrimaryGleasonPattern": 3,

"SecondaryGleasonPattern": 4,

"TotalGleasonScore": 7

}

2. Gleason-Score: "4+3=7b"

Denken wir Schritt für Schritt:

- Der Wert ist im Additionsformat "4+3=7b".

- Die erste Zahl "4" ist der Primary Gleason pattern.

- Die zweite Zahl "3" ist der Secondary Gleason pattern.

- Die Zahl nach dem "=" ist "7", das der Total Gleason score entspricht.

Antwort:

{

"PrimaryGleasonPattern": 4,

"SecondaryGleasonPattern": 3,

"TotalGleasonScore": 7

}

3. Gleason-Score: "8"

Denken wir Schritt für Schritt:

- Der Wert ist eine reine Zahl "8".

- Die Primary Gleason pattern und Secondary Gleason pattern können nicht bestimmt werden.

- Der Total Gleason score ist "8".

Antwort:

{

"PrimaryGleasonPattern": "Unknown",

"SecondaryGleasonPattern": "Unknown",

"TotalGleasonScore": 8

}

4. Gleason-Score: "7a"

Denken wir Schritt für Schritt:

- Der Wert ist "7a".

- Basierend auf den bekannten Regeln: "7a" entspricht Primary: 3, Secondary: 4, Total: 7.

Antwort:

{

"PrimaryGleasonPattern": 3,

"SecondaryGleasonPattern": 4,

"TotalGleasonScore": 7

}

5. Gleason-Score: "7"

Denken wir Schritt für Schritt:

- Der Wert ist eine reine Zahl "7".

- Die Primary Gleason pattern und Secondary Gleason pattern können nicht bestimmt werden.

- Der Total Gleason score ist "7".

Antwort:

{

"PrimaryGleasonPattern": "Unknown",

"SecondaryGleasonPattern": "Unknown",

"TotalGleasonScore": 7

}

### Antwort Format:

{

"PrimaryGleasonPattern": "<1, 2, 3, 4, 5 oder 'Unknown'>",

"SecondaryGleasonPattern": "<1, 2, 3, 4, 5 oder 'Unknown'>",

"TotalGleasonScore": "<Numerischer Wert oder 'Unknown'>"

}

Gleason-Score:

"""

},

{

"name": "PSAList",

"prompt": """

### Query:

Bitte extrahiere den gesamten Abschnitt, der mit "Tumormarker" oder "PSA-Verlauf" beginnt, aus dem bereitgestellten Text und gib ausschließlich ein einziges JSON-Objekt zurück. Zusätzliche Texte, Erklärungen oder mehrere JSON-Objekte sind nicht erlaubt.

### Schritte und Regeln:

1. Suche im Text nach einem Abschnitt, der mit "Tumormarker" oder "PSA-Verlauf" beginnt.

2. Extrahiere alle Informationen, die direkt nach "Tumormarker" oder "PSA-Verlauf" folgen, einschließlich Werte, Daten oder Notizen.

3. Wenn kein Abschnitt mit "Tumormarker" oder "PSA-Verlauf" gefunden wird, gib "Unknown" zurück.

### Beispiele:

1. Text: "Tumormarker PSA [ng/ml]: 6,86 (06/22); 5,89 (09/22)"

Denken wir Schritt für Schritt:

- Der Abschnitt beginnt mit "Tumormarker PSA [ng/ml]".

- Extrahiere alle nachfolgenden Inhalte: "6,86 (06/22); 5,89 (09/22)".

Antwort:

{

"PSAList": "Tumormarker PSA [ng/ml]: 6,86 (06/22); 5,89 (09/22)"

}

2. Text: "Tumormarker PSA (ng/ml): 06/16: 4,2, 11/16: 4,6; 06/17: 4,4; 11/19: 7,15; 12/19: 7,3 Aktuell: 05/20: 6,7"

Denken wir Schritt für Schritt:

- Der Abschnitt beginnt mit "Tumormarker PSA (ng/ml)".

- Extrahiere alle nachfolgenden Inhalte: "06/16: 4,2, 11/16: 4,6; 06/17: 4,4; 11/19: 7,15; 12/19: 7,3 Aktuell: 05/20: 6,7".

Antwort:

{

"PSAList": "Tumormarker PSA (ng/ml): 06/16: 4,2, 11/16: 4,6; 06/17: 4,4; 11/19: 7,15; 12/19: 7,3 Aktuell: 05/20: 6,7"

}

3. Text: "Keine Erwähnung von Tumormarkern im Bericht."

Denken wir Schritt für Schritt:

- Es gibt keinen Abschnitt, der mit "Tumormarker" beginnt.

- Gib "Unknown" zurück.

Antwort:

{

"PSAList": "Unknown"

}

4. Text: "PSA-Verlauf (ng/ml): 5,29 (03/23); 5,02 (05/23); 5,85 (10/23); 7,47 (01/24); 7,99 (04/24); 9,9 (06/24)"

Denken wir Schritt für Schritt:

- Der Abschnitt beginnt mit "PSA-Verlauf".

- Extrahiere alle nachfolgenden Inhalte: "5,29 (03/23); 5,02 (05/23); 5,85 (10/23); 7,47 (01/24); 7,99 (04/24); 9,9 (06/24)".

Antwort:

{

"PSAList": "PSA-Verlauf (ng/ml): 5,29 (03/23); 5,02 (05/23); 5,85 (10/23); 7,47 (01/24); 7,99 (04/24); 9,9 (06/24)"

}

5. Text: "Tumormarker iPSA: 14,1 ng/ml"

Denken wir Schritt für Schritt:

- Der Abschnitt beginnt mit "Tumormarker".

- Extrahiere alle nachfolgenden Inhalte: "14,1 ng/ml".

Antwort:

{

"PSAList": "Tumormarker iPSA: 14,1 ng/ml"

}

### Antwort Format:

{

"PSAList": "<Extrahierter Abschnitt oder 'Unknown'>"

}

"""

},

{

"name": "PSA",

"prompt": """

### Query:

Extrahiere den letzten PSA-Wert (Prostata-spezifisches Antigen) und das zugehörige Datum aus dem bereitgestellten Text. Gib ausschließlich ein einziges JSON-Objekt zurück. Zusätzliche Texte, Erklärungen oder mehrere Objekte sind nicht erlaubt.

### Schritte und Regeln:

1. Suche im Text nach einer Liste mit PSA-Werten und zugehörigen Datumsangaben.

2. Identifiziere das Trennzeichen der Liste:

- Die Liste kann durch Semikolons (;) oder Kommas (,) getrennt sein.

- Das Modell muss das richtige Trennzeichen selbstständig erkennen und die Liste entsprechend aufteilen.

3. Wähle ausschließlich den letzten Eintrag der Liste aus:

- Nur der letzte PSA-Wert und das zugehörige Datum sind relevant.

- Alle anderen Einträge werden ignoriert.

4. Extrahiere das Datum:

- Das Datum kann vor oder nach dem PSA-Wert erscheinen.

- Wenn ein vollständiges Datum (Tag, Monat, Jahr) vorhanden ist, gib es im Format "DD-MM-YYYY" zurück.

- Wenn nur Monat und Jahr angegeben sind (z. B. "MM/YY"), interpretiere es als den ersten Tag des Monats und gib das Datum im Format "01-MM-YYYY" zurück.

- Wenn kein Datum gefunden wird, gib "01-01-1901" zurück.

5. Extrahiere den PSA-Wert:

- Der PSA-Wert ist eine numerische Angabe in der Nähe des Datums.

- Beachte, dass iPSA und PSA gleichwertig sind. Wähle immer und ausschließlich den letzten Wert (PSA oder iPSA) aus der Liste.

6. Bestimme `IsPSADetermined`:

- Wenn PSA-Werte im Text gefunden wurden, setze "IsPSADetermined": "1".

- Wenn keine PSA-Werte im Text gefunden wurden, setze "IsPSADetermined": "0".

### Beispiele:

1. Text: "Tumormarker (PSA, ng/ml): iPSA: 01.03.23: 4,53 ng/ml, (11/2008: 1,63 ng/ml); 4/21: 4,6; 6/21: 3,66; 1/23: 4,08"

Denken wir Schritt für Schritt:

- Gefundene Liste: "01.03.23: 4,53 ng/ml, (11/2008: 1,63 ng/ml); 4/21: 4,6; 6/21: 3,66; 1/23: 4,08".

- Identifikation des Trennzeichens: Die Werte sind durch Semikolons (;) getrennt.

- Letzter Eintrag: "1/23: 4,08".

- Extrahiertes Datum: "1/23" enthält nur Monat und Jahr.

- Gemäß den Regeln wird der erste Tag des Monats angenommen, das Datum wird als "01-01-2023" formatiert.

- Extrahierter PSA-Wert: "4,08".

Antwort:

{

"IsPSADetermined": "1",

"PSADate": "01-01-2023",

"PSALevel": "4.08"

}

2. Text: "PSA [ng/ml]: 5,27µg/l, 21.04.2020; 7,28µg/l, 23.04.2021"

Denken wir Schritt für Schritt:

- Gefundene Liste: "5,27µg/l, 21.04.2020; 7,28µg/l, 23.04.2021".

- Identifikation des Trennzeichens: Die Werte sind durch Semikolons (;) getrennt.

- Letzter Eintrag: "7,28µg/l, 23.04.2021".

- Extrahiertes Datum: "23.04.2021" enthält Tag, Monat und Jahr.

- Das Datum wird unverändert im Format "23-04-2021" zurückgegeben.

- Extrahierter PSA-Wert: "7,28".

Antwort:

{

"IsPSADetermined": "1",

"PSADate": "23-04-2021",

"PSALevel": "7.28"

}

3. Text: "PSA [ng/ml]: 14"

Denken wir Schritt für Schritt:

- Gefundene Liste: "14".

- Identifikation des Trennzeichens: Es gibt keine erkennbaren Trennzeichen.

- Letzter Eintrag: "14".

- Kein Datum gefunden, daher wird das Standarddatum "01-01-1901" zurückgegeben.

- Extrahierter PSA-Wert: "14".

Antwort:

{

"IsPSADetermined": "1",

"PSADate": "01-01-1901",

"PSALevel": "14"

}

4. Text: "Tumormarker: PSA 8,13 ng/ml 11/2023, 5,57 ng/ml 11/2019"

Denken wir Schritt für Schritt:

- Gefundene Liste: "PSA 8,13 ng/ml 11/2023, 5,57 ng/ml 11/2019".

- Identifikation des Trennzeichens: Die Werte sind durch Kommas (,) getrennt.

- Letzter Eintrag: "5,57 ng/ml 11/2019".

- Extrahiertes Datum: "11/2019" enthält nur Monat und Jahr.

- Es wird überprüft, ob Tag, Monat und Jahr vollständig sind. Da nur Monat und Jahr angegeben sind, wird gemäß den Regeln der erste Tag des Monats angenommen.

- Das Datum wird als "01-11-2019" formatiert.

- Extrahierter PSA-Wert: "5,57".

Antwort:

{

"IsPSADetermined": "1",

"PSADate": "01-11-2019",

"PSALevel": "5.57"

}

5. Text: "Tumormarker: PSA-Verlauf (ng/ml): 3,33 (07/22); 4,14 (10/22); 5,29 (03/23)"

Denken wir Schritt für Schritt:

- Gefundene Liste: "3,33 (07/22); 4,14 (10/22); 5,29 (03/23)".

- Identifikation des Trennzeichens: Die Werte sind durch Semikolons (;) getrennt.

- Letzter Eintrag: "5,29 (03/23)".

- Extrahiertes Datum: "03/23" enthält nur Monat und Jahr.

- Es wird überprüft, ob Tag, Monat und Jahr vollständig sind. Da nur Monat und Jahr angegeben sind, wird gemäß den Regeln der erste Tag des Monats angenommen.

- Das Datum wird als "01-03-2023" formatiert.

- Extrahierter PSA-Wert: "5,29".

Antwort:

{

"IsPSADetermined": "1",

"PSADate": "01-03-2023",

"PSALevel": "5.29"

}

### Antwort Format:

{

"IsPSADetermined": "<1 oder 0>",

"PSADate": "<PSA-Datum im Format 'DD-MM-YYYY', '01-MM-YYYY' oder '01-01-1901'>",

"PSALevel": "<PSA-Wert in ng/ml>"

}

"""

},

{

"name": "BASE_ECOG",

"prompt": """

### Query

Bitte extrahiere den ECOG-Status des Patienten aus dem Text und gib ausschließlich ein einziges JSON-Objekt auf Deutsch zurück. Zusätzliche Texte, Erklärungen oder mehrere JSON-Objekte sind nicht erlaubt.

**Vorprüfung – Wichtig:**

Bevor du beginnst: Prüfe, ob im Text **eine der folgenden Formulierungen** eindeutig vorkommt:

- „ECOG“

- „ECOG-Status“

- „ECOG I–IV“

**Wenn keine dieser Formulierungen vorkommt**, beende die Aufgabe sofort und gib exakt dieses JSON zurück:

```json

{

"BASE_ECOG_YN": "0",

"BASE_ECOG_DATE": null,

"BASE_ECOG": null

}

```

**Wenn eine dieser Formulierungen vorhanden ist**, fahre mit den folgenden Extraktionsregeln fort.

*Wichtig:** Alle Datumsangaben müssen im Format `DD-MM-YYYY` angegeben werden – z. B. `13-09-2023`.

**Andere Formate wie `13.09.2023`, `2023-09-13` oder ausgeschriebene Daten sind nicht erlaubt.**

### Schritte und Regeln:

1. **ECOG nur bei expliziter Nennung**

Der ECOG-Status darf **nur dann extrahiert werden**, wenn im Text **eindeutig** einer der folgenden Begriffe enthalten ist (Groß-/Kleinschreibung egal):

- „ECOG“

- „ECOG-Status“

- „ECOG I-IV“

**Wenn keiner dieser Begriffe vorkommt**, darf **kein ECOG-Wert extrahiert werden**, auch nicht basierend auf einer Beschreibung des Allgemeinzustands.

2. **Ungültige Begriffe (nie als ECOG interpretieren):**

- „CTCAE“, „CTC“, „RTOG“

- „WHO-Performance“, „Karnofsky“

- „Allgemeinzustand“, „Grad“, „pflegebedürftig“, „bettlägerig“, „60%“, „100“, „ECOG-like“

3. **Gültige ECOG-Werte (nur wenn direkt bei ECOG-Nennung genannt):**

- Nur die Werte „0“, „1“, „2“, „3“ oder „4“ (auch römisch I–IV)

- Keine Prozentsätze, Grade, freien Beschreibungen etc.

4. **Datumsregel (nur wenn ECOG erkannt wurde):**

- Wenn ein Datum **direkt beim ECOG** genannt ist → verwende dieses.

- Falls nicht vorhanden: Verwende das **Datum der Patientenvorstellung** – also ein **explizites, einzelnes Vorstellungsdatum** wie z. B.:

> „Die Patientin stellte sich am 13.09.2023 in unserer Ambulanz vor.“

- **Achtung:** Datumsangaben aus einem **Behandlungszeitraum** dürfen **nicht** verwendet werden. Beispiel (ungültig):

> „…der sich vom 04.08. bis zum 15.08.2023 in unserer ambulanten radioonkologischen Behandlung befand“

- Falls kein Vorstellungdatum vorhanden ist: Verwende das **Dokumentdatum**.

- Wenn **kein zulässiges Datum gefunden werden kann**, setze das Datum auf `"01-01-1901"`.

- Wenn **kein ECOG erkannt wurde**, setze das Datum auf `null`.

5. **Datumsformat:**

- Immer exakt **DD-MM-YYYY** (z. B. „04-09-2021“)

- Keine Punkte, keine ISO-Formate, kein Textdatum

- Unsichere Daten → `"01-01-1901"` (falls ECOG vorhanden) oder `null` (falls nicht)

6. **Antwortformatierung:**

- `"BASE_ECOG_YN"` = `"1"`, wenn ein gültiger ECOG-Status extrahiert wurde

- `"BASE_ECOG_YN"` = `"0"`, **wenn kein ECOG im Text erkannt wurde**

- `"BASE_ECOG_DATE"` = Datum im Format `"DD-MM-YYYY"` oder `"01-01-1901"` oder `null`

- `"BASE_ECOG"` = ECOG-Wert zwischen `"0"` und `"4"` als String oder `null`

---

### Denke Schritt für Schritt:

1. **Kommt im Text eine der zulässigen ECOG-Formulierungen vor?**

→ Wenn **Nein**, antworte sofort mit:

```json

{

"BASE_ECOG_YN": "0",

"BASE_ECOG_DATE": null,

"BASE_ECOG": null

}

```

→ Wenn **Ja**, fahre fort mit der Extraktion von Wert und Datum.

2. **Ist der ECOG-Wert gültig?** (Nur 0–4, ggf. römisch)

→ Wenn **ungültig**, ebenfalls wie oben antworten.

→ Wenn **gültig**, extrahiere Datum gemäß Regel 4.

3. **Welches Datum soll verwendet werden?**

- Zuerst: Gibt es ein **Datum direkt bei der ECOG-Angabe**? → Verwende dieses.

- Falls nicht: Suche nach einem **klaren Vorstellungstermin**, z. B.:

> „Die Patientin stellte sich am 14.03.2023 vor.“

- **Achtung:** Wenn im Text ein **Behandlungszeitraum** genannt wird, z. B.:

> „…der sich vom 04.08. bis zum 15.08.2023 in Behandlung befand“

→ **Dieser Zeitraum darf nicht als Datum verwendet werden.**

- Falls kein Vorstellungstermin vorhanden ist: Verwende das **Dokumentdatum**.

- Wenn kein gültiges Datum erkennbar ist → `"01-01-1901"` verwenden.

4. **Gib am Ende ausschließlich das JSON-Objekt im korrekten Format zurück.**

---

### Beispiele:

1. Text: "Der ECOG-Status beträgt 0"

Denken wir Schritt für Schritt:

- Der Text enthält die Formulierung „ECOG-Status beträgt 0“, was gültig ist.

- Kein Datum vorhanden → Standardwert verwenden

Antwort:

{

"BASE_ECOG_YN": "1",

"BASE_ECOG_DATE": "01-01-1901",

"BASE_ECOG": "0"

}

---

2. Text: "ECOG III laut Verlaufsnotiz vom 12.01.2022."

Denken wir Schritt für Schritt:

- „ECOG III“ ist eine gültige Formulierung → Wert = 3

- Datum ist vorhanden → 12-01-2022

Antwort:

{

"BASE_ECOG_YN": "1",

"BASE_ECOG_DATE": "12-01-2022",

"BASE_ECOG": "3"

}

---

3. Text: "Die Patientin ist bettlägerig, benötigt durchgehend Hilfe."

Denken wir Schritt für Schritt:

- Es wird kein „ECOG“, „ECOG-Status“ etc. erwähnt. Nur eine Beschreibung.

- Kein ECOG-Wert darf extrahiert werden.

Antwort:

{

"BASE_ECOG_YN": "0",

"BASE_ECOG_DATE": null,

"BASE_ECOG": null

}

---

4. Text: "ECOG II laut Verlaufsnotiz. Die Patientin befand sich vom 04.08. bis zum 15.08.2023 in Behandlung."

Denken wir Schritt für Schritt:

- „ECOG II“ ist eine gültige Angabe → Wert = 2

- Es ist **kein explizites Einzel-Datum** vorhanden

- Der Behandlungszeitraum vom 04.08. bis 15.08.2023 darf **nicht verwendet** werden

- Es gibt kein anderes gültiges Datum → Standardwert verwenden

Antwort:

```json

{

"BASE_ECOG_YN": "1",

"BASE_ECOG_DATE": "01-01-1901",

"BASE_ECOG": "2"

}

---

5. Text: "Karnofsky 60%, Allgemeinzustand eingeschränkt."

Denken wir Schritt für Schritt:

- Keine zulässige Formulierung wie „ECOG“ enthalten.

- Kein ECOG-Wert extrahierbar.

Antwort:

{

"BASE_ECOG_YN": "0",

"BASE_ECOG_DATE": null,

"BASE_ECOG": null

}

---

### Antwortformat:

{

"BASE_ECOG_YN": "<'1' oder '0'>",

"BASE_ECOG_DATE": "<Datum im Format DD-MM-YYYY oder '01-01-1901' oder null>",

"BASE_ECOG": "<Zahl zwischen '0' und '4' oder null>"

}

"""

}

]

Supplementary Text S1 c) user_prompts_FU3M user_prompts_FU3M = [

{

"name": "FU3M_KPSS",

"prompt": """

### Query:

Bitte extrahiere die KPSS-Informationen aus dem Text und gib ausschließlich ein einziges JSON-Objekt zurück. Zusätzliche Texte, Erklärungen oder mehrere JSON-Objekte sind nicht erlaubt.

Die KPSS-Informationen bestehen aus dem Karnofsky-Index (FU3M_KPSSValue), der den allgemeinen Gesundheitszustand eines Patienten in Prozent angibt, und dem zugehörigen Datum (FU3M_KPSSDate), das angibt, wann dieser Wert dokumentiert wurde. Falls keine KPSS-Informationen im Text vorhanden sind, wird dies entsprechend als "Unknown" gekennzeichnet.

### Schritte und Regeln:

1. **Suche nach dem Begriff "Karnofsky-Index" oder einer Variante wie "Karnofsky".**

- Falls **kein solcher Begriff im Text vorkommt**:

- Setze `"FU3M_IsKPSSDetermined"` auf `"0"`.

- Setze `"FU3M_KPSSValue"` auf `null`.

- Setze `"FU3M_KPSSDate"` auf `null`.

- **Beende die Extraktion an dieser Stelle.**

- **Es dürfen keine Werte geraten, geschätzt oder auf andere Weise abgeleitet werden.**

2. **Wenn "Karnofsky" gefunden wurde:**

- **Extrahiere den FU3M_KPSSValue:**

- Der Wert steht direkt hinter dem gefundenen Begriff.

- Falls ein Bereich wie "80 – 90 %" angegeben ist, wähle den **niedrigeren Wert** (hier: `"80"`).

- Falls **kein klarer Wert angegeben ist**, obwohl "Karnofsky" vorkommt:

- Setze `"FU3M_KPSSValue"` auf `"Unknown"`.

- Setze `"FU3M_IsKPSSDetermined"` auf `"1"`.

- Setze `"FU3M_KPSSDate"` auf `null`.

- **Beende die Extraktion an dieser Stelle.**

- **Extrahiere das Datum FU3M_KPSSDate:**

- Suche im Abschnitt, der mit „Sehr geehrter“ beginnt, nach dem Ausdruck **„der sich am <Datum>“**.

- Falls ein solches Datum vorhanden ist, gib es im Format `"DD-MM-YYYY"` zurück.

- Falls kein Datum gefunden wird, verwende `"01-01-1901"`.

3. **Zusammenfassend gilt:**

- Nur wenn `"FU3M_IsKPSSDetermined"` den Wert `"1"` hat, dürfen `"FU3M_KPSSValue"` und `"FU3M_KPSSDate"` **einen gültigen Wert enthalten**.

- Wenn `"FU3M_IsKPSSDetermined"` den Wert `"0"` hat, müssen **beide anderen Felder zwingend `null` sein** – selbst wenn im Text ein Datum genannt wird.

---

### Formatierungsregeln für das Datum:

- Wenn das Datum Tag, Monat und Jahr enthält, gib es im Format `"DD-MM-YYYY"` zurück.

- Falls kein Datum gefunden wird, gib `"01-01-1901"` zurück.

- Falls kein KPSS-Wert extrahiert wurde, muss das Datum `null` sein.

---

### Formatierungsregeln für das Datum:

- Wenn das Datum Tag, Monat und Jahr enthält, gib es im Format "DD-MM-YYYY" zurück.

- Falls kein Datum gefunden wird, gib "01-01-1901" zurück.

### Beispiele:

1. Text:

Sehr geehrter Herr Kollege,

wir berichten über Herrn [ANONYMIZED], der sich am 17.10.2024 in unserer Ambulanz 3 nach Abschluss der o. g. Strahlentherapie zur strahlentherapeutischen Nachschau wieder vorstellte.

Herr [ANONYMIZED] berichtete mäßiges Wohlbefinden und eingeschränkte Belastbarkeit, dies sei aber auf Grund seiner kardialen Erkrankungen schon länger bekannt und konstant. Stuhlgang und Wasserlassen bereiten keine Probleme. Bisher sei keine ambulante Vorstellung beim Urologen erfolgt.

Aktueller PSA: 0,91 µg/l, IPSS 3

Untersuchungsbefund:

Karnofsky-Index 80 -90 %, Körpergewicht 90 kg.

Denken wir Schritt für Schritt:

- Der Begriff "Karnofsky-Index" ist vorhanden.

- Die Werte sind "80 - 90 %". Der niedrigere Wert ist "80".

- Das Datum steht hinter "der sich am" und lautet "17.10.2024".

- Das Datum wird ins Format "DD-MM-YYYY" konvertiert: "17-10-2024".

- Setze "FU3M_IsKPSSDetermined" auf 1.

Antwort:

{

"FU3M_IsKPSSDetermined": "1",

"FU3M_KPSSDate": "17-10-2024",

"FU3M_KPSSValue": "80"

}

2. Text:

Sehr geehrter Herr Kollege,

wir berichten über Herrn [ANONYMIZED], der sich am 16.06.2023 in unserer Ambulanz 3 nach Abschluss der o. g. Strahlentherapie zur strahlentherapeutischen Nachschau wieder vorstellte.

Herr [ANONYMIZED] berichtete Wohlbefinden und gute Belastbarkeit. Stuhlgang und Wasserlassen bereiten keine Probleme. Die letzte Nachsorge erfolgte am 04.2023 Urologe und war unauffällig.

Aktueller PSA: 7,75 ng/ml

Die antiandrogene Therapie mit einem GnRH-Analogon wird nicht durchgeführt.

Untersuchungsbefund:

Karnofsky-Index 100 %, Körpergewicht 74 kg.

Denken wir Schritt für Schritt:

- Der Begriff "Karnofsky-Index" wird im Abschnitt "Untersuchungsbefund" gefunden.

- Direkt hinter dem Begriff steht der Wert "100 %". Da es sich um einen eindeutigen Wert handelt und kein Zahlenbereich vorliegt, wird "100" als FU3M_KPSSValue übernommen.

- Anschließend wird im Abschnitt, nach dem Ausdruck "der sich am" gesucht, um das FU3M_KPSSDate zu bestimmen. Es wird der Text "der sich am 16.06.2023" identifiziert.

- Das Datum "16.06.2023" wird gemäß den Formatierungsregeln ins Format "DD-MM-YYYY" umgewandelt und ergibt "16-06-2023".

- Setze "FU3M_IsKPSSDetermined" auf 1.

Antwort:

{

"FU3M_IsKPSSDetermined": "1",

"FU3M_KPSSDate": "16-06-2023",

"FU3M_KPSSValue": "100"

}

3. Text:

Sehr geehrter Herr Kollege,

wir berichten über Herrn [ANONYMIZED] 3 Monate nach Abschluss der o.g. Strahlentherapie zur strahlentherapeutischen Nachschau wieder vorstellte.

Herr [ANONYMIZED] welche nach der OP bereits deutlich gebessert war und nach Bestrahlung nun wieder verschlechtert vorliegt. Vorlagen werden bei Terminen außerhalb genutzt. Der Beckenboden wird seit der Reha regelmäßig beübt. Die letzte uroonkologische Nachsorge war unauffällig. Ein nächster Termin ist für den 26.04.21 eingeplant.

Aktueller PSA: <0,0 (1/2021) unter ADT (seit 10/2020)

Untersuchungsbefund:

Karnofsky-Index 90 %, Körpergewicht 85,5 kg, konstant.

Denken wir Schritt für Schritt:

- Der Begriff "Karnofsky-Index" wird im Abschnitt "Untersuchungsbefund" gefunden.

- Direkt dahinter steht der Wert "90 %". Da es sich um einen eindeutigen Wert handelt, wird "90" als FU3M_KPSSValue extrahiert.

- Anschließend wird im Abschnitt, der mit "Sehr geehrter" beginnt, nach dem Ausdruck "der sich am" gesucht, um das Datum für FU3M_KPSSDate zu bestimmen.

- Da kein "der sich am"-Datum vorhanden ist und die Angabe "3 Monate nach Abschluss der o.g. Strahlentherapie" kein konkretes Datum liefert, wird gemäß den Regeln das Standarddatum "01-01-1901" verwendet.

- Da der Wert erfolgreich extrahiert wurde, wird FU3M_IsKPSSDetermined auf 1 gesetzt.

Antwort:

{

"FU3M_IsKPSSDetermined": "1",

"FU3M_KPSSDate": "01-01-1901",

"FU3M_KPSSValue": "90"

}

4. Text:

Wir berichten über Herrn [ANONYMIZED], der sich am 08.09.2023 in unserer Ambulanz 4 nach Abschluss der o. g. Strahlentherapie zur strahlentherapeutischen Nachschau wieder vorstellte.

Herr [ANONYMIZED] berichtete Wohlbefinden und gute Belastbarkeit. Stuhlgang und Wasserlassen bereiten keine Probleme. Die letzte Nachsorge erfolgte Ihnen und war unauffällig.

Aktueller PSA: <0,0006

Untersuchungsbefund:

Karnofsky-Index 100 %.

Denken wir Schritt für Schritt:

- Der Begriff "Karnofsky-Index" wird im Abschnitt "Untersuchungsbefund" gefunden.

- Der Wert direkt dahinter lautet "100 %". Da es sich um eine einzelne Zahl handelt, wird dieser Wert direkt als FU3M_KPSSValue übernommen.

- Anschließend wird im Abschnitt, der mit "Sehr geehrter" beginnt, nach dem Ausdruck "der sich am" gesucht, um das Datum für FU3M_KPSSDate zu bestimmen. In der Einleitung findet sich die Datumsangabe "08.09.2023".

- Es enthält Tag, Monat und Jahr und wird im Format "DD-MM-YYYY" übernommen: "08-09-2023".

- Setze "FU3M_IsKPSSDetermined" auf 1.

Antwort:

{

"FU3M_IsKPSSDetermined": "1",

"FU3M_KPSSDate": "08-09-2023",

"FU3M_KPSSValue": "100"

}

5. Text:

Sehr geehrter Herr Kollege,

wir berichten über Herrn [ANONYMIZED], der sich am 04.03.2022 in unserer Ambulanz 3 nach Abschluss der o. g. Strahlentherapie zur strahlentherapeutischen Nachschau wieder vorstellte.

Wir danken für die Mitgabe des aktuellen PSA-Verlaufs.

Aktueller PSA-Wert: 1,43 ng/ml. Eine antihormonelle Therapie erfolgt nicht.

Wir haben folgende Befunde erhoben:

Parameter: Wert (abzüglich Baseline bei CTC) Falls G3+Nebenwirkung:

Denken wir Schritt für Schritt:

- Im gesamten Text wird der Begriff "Karnofsky-Index" nicht gefunden.

- Da kein Wert für den Karnofsky-Index vorhanden ist, wird FU3M_KPSSValue gemäß den Regeln auf "Unknown" gesetzt.

- Da kein Wert gefunden wurde, erübrigt sich die Suche nach dem Datum.

- FU3M_KPSSDate wird daher auf das Standarddatum "01-01-1901" gesetzt.

- Da weder ein Wert noch ein Datum bestimmt werden konnte, wird FU3M_IsKPSSDetermined auf 0 gesetzt.

Antwort:

{

"FU3M_IsKPSSDetermined": "0",

"FU3M_KPSSDate": null,

"FU3M_KPSSValue": null

}

### Antwort Format:

{

"FU3M_IsKPSSDetermined": "<1 oder 0>",

"FU3M_KPSSDate": "<Datum im Format DD-MM-YYYY , '01-01-1901' oder null>",

"FU3M_KPSSValue": "<KPSS-Wert oder 'Unknown'>"

}

"""

},

{

"name": "FU3M_PSAList",

"prompt": """

### Query:

Bitte extrahiere den gesamten Abschnitt, der mit "Tumormarker" oder "PSA-Verlauf" beginnt, aus dem bereitgestellten Text und gib ausschließlich ein einziges JSON-Objekt zurück. Zusätzliche Texte, Erklärungen oder mehrere JSON-Objekte sind nicht erlaubt.

### Schritte und Regeln:

1. Suche im Text nach einem Abschnitt, der mit "Tumormarker" oder "PSA-Verlauf" beginnt.

2. Extrahiere alle Informationen, die direkt nach "Tumormarker" oder "PSA-Verlauf" folgen, einschließlich Werte, Daten oder Notizen.

3. Wenn kein Abschnitt mit "Tumormarker" oder "PSA-Verlauf" gefunden wird, gib "Unknown" zurück.

### Beispiele:

1. Text: "Tumormarker PSA [ng/ml]: 6,86 (06/22); 5,89 (09/22)"

Denken wir Schritt für Schritt:

- Der Abschnitt beginnt mit "Tumormarker PSA [ng/ml]".

- Extrahiere alle nachfolgenden Inhalte: "6,86 (06/22); 5,89 (09/22)".

Antwort:

{

"FU3M_PSAList": "Tumormarker PSA [ng/ml]: 6,86 (06/22); 5,89 (09/22)"

}

2. Text: "Tumormarker PSA (ng/ml): 06/16: 4,2, 11/16: 4,6; 06/17: 4,4; 11/19: 7,15; 12/19: 7,3 Aktuell: 05/20: 6,7"

Denken wir Schritt für Schritt:

- Der Abschnitt beginnt mit "Tumormarker PSA (ng/ml)".

- Extrahiere alle nachfolgenden Inhalte: "06/16: 4,2, 11/16: 4,6; 06/17: 4,4; 11/19: 7,15; 12/19: 7,3 Aktuell: 05/20: 6,7".

Antwort:

{

"FU3M_PSAList": "Tumormarker PSA (ng/ml): 06/16: 4,2, 11/16: 4,6; 06/17: 4,4; 11/19: 7,15; 12/19: 7,3 Aktuell: 05/20: 6,7"

}

3. Text: "Keine Erwähnung von Tumormarkern im Bericht."

Denken wir Schritt für Schritt:

- Es gibt keinen Abschnitt, der mit "Tumormarker" beginnt.

- Gib "Unknown" zurück.

Antwort:

{

"FU3M_PSAList": "Unknown"

}

4. Text: "PSA-Verlauf (ng/ml): 5,29 (03/23); 5,02 (05/23); 5,85 (10/23); 7,47 (01/24); 7,99 (04/24); 9,9 (06/24)"

Denken wir Schritt für Schritt:

- Der Abschnitt beginnt mit "PSA-Verlauf".

- Extrahiere alle nachfolgenden Inhalte: "5,29 (03/23); 5,02 (05/23); 5,85 (10/23); 7,47 (01/24); 7,99 (04/24); 9,9 (06/24)".

Antwort:

{

"FU3M_PSAList": "PSA-Verlauf (ng/ml): 5,29 (03/23); 5,02 (05/23); 5,85 (10/23); 7,47 (01/24); 7,99 (04/24); 9,9 (06/24)"

}

5. Text: "Tumormarker iPSA: 14,1 ng/ml"

Denken wir Schritt für Schritt:

- Der Abschnitt beginnt mit "Tumormarker".

- Extrahiere alle nachfolgenden Inhalte: "14,1 ng/ml".

Antwort:

{

"FU3M_PSAList": "Tumormarker iPSA: 14,1 ng/ml"

}

### Antwort Format:

{

"FU3M_PSAList": "<Extrahierter Abschnitt oder 'Unknown'>"

}

"""

},

{

"name": "FU3M_PSA",

"prompt": """

### Query:

Extrahiere den PSA-Wert (Prostata-spezifisches Antigen) und das zugehörige Datum aus dem bereitgestellten Text. Die Werte befinden sich entweder in der PSA-Liste am Anfang des Dokuments oder innerhalb der "Sehr geehrter"-Einleitung und am Ende des Textes. Gib ausschließlich ein einziges JSON-Objekt zurück. Zusätzliche Texte, Erklärungen oder mehrere Objekte sind nicht erlaubt.

Der extrahierte Wert FU3M_PSALevel bezieht sich auf den PSA-Wert der Nachsorgeuntersuchung und spiegelt den aktuellen PSA-Status des Patienten wider.

### Schritte und Regeln:

1. Suche in der "Sehr geehrter"-Einleitung oder am Ende des Textes nach einem PSA-Wert.

- Relevante Begriffe zur Identifikation des richtigen Wertes sind: "PSA-Wertes", "Aktueller PSA", "Aktueller PSA-Wert", "PSA", "PSA Wert".

- Der PSA-Wert ist die Zahl, die in unmittelbarer Nähe dieser Begriffe steht.

2. Bestimme den PSA-Wert:

- Extrahiere die Zahl, die direkt neben den oben genannten Begriffen steht.

- Falls mehrere PSA-Werte im selben Abschnitt vorkommen, wähle den aktuellen Nachsorge-PSA-Wert anhand der Position und Formulierung.

- Falls kein PSA-Wert in der "Sehr geehrter"-Einleitung oder am Textende gefunden wird, überprüfe die PSA-Liste am Dokumentanfang.

- Wähle den letzten PSA-Wert aus der Liste am Dokumentanfang.

3. Bestimme das zugehörige Datum:

- Suche nach einem Datum direkt vor oder nach dem gefundenen PSA-Wert.

- Falls kein solches Datum vorhanden ist, überprüfe die PSA-Liste am Dokumentanfang:

- Die PSA-Liste enthält PSA-Werte aus verschiedenen Zeitpunkten, getrennt durch Kommas oder Semikolons.

- Jede Einheit in der PSA-Liste besteht aus einem PSA-Wert und möglicherweise einem zugehörigen Testdatum.

- Falls der letzte PSA-Wert aus der Liste mit dem extrahierten PSA-Wert übereinstimmt, übernehme dessen Datum.

- Falls kein übereinstimmender PSA-Wert in der Liste gefunden wird, überprüfe, ob nach "der sich am" ein Datum steht. Falls ja, verwende dieses Datum.

- Falls kein Datum nach "der sich am" gefunden wird, setze das Datum auf "01-01-1901".

4. Falls in der "Sehr geehrter"-Einleitung oder am Ende des Textes kein PSA-Wert gefunden wird:

- Überprüfe den letzten PSA-Wert in der PSA-Liste am Dokumentanfang.

- Wähle den letzten PSA-Wert aus der Liste.

- Falls ein Datum vorhanden ist, verwende dieses Datum.

- Falls kein Datum verfügbar ist, überprüfe, ob nach "der sich am" ein Datum steht. Falls ja, verwende dieses.

- Falls kein Datum nach "der sich am" gefunden wird, gib "01-01-1901" zurück.

5. Bestimme FU3M_IsPSADetermined:

- Wenn ein PSA-Wert gefunden wird, setze "FU3M_IsPSADetermined": "1".

- Wenn kein PSA-Wert gefunden wird, setze "FU3M_IsPSADetermined": "0", "FU3M_PSADate": "01-01-1901", "FU3M_PSALevel": "Unknown".

### Formatierungsregeln für das Datum:

- Falls ein Datum mit Tag, Monat und Jahr vorhanden ist, gib es im Format "DD-MM-YYYY" zurück.

- Falls nur Monat und Jahr angegeben sind (z. B. "MM/YY"), interpretiere es als den ersten Tag des Monats und gib das Datum im Format "01-MM-YYYY" zurück.

- Falls nur das Jahr vorhanden ist, interpretiere es als "01-01-YYYY".

- Falls kein Datum gefunden wird, gib "01-01-1901" zurück.

### Beispiele:

1. Text:

Tumormarker PSA [ng/ml];: 5,8 (07/21)

Sehr geehrter Herr Kollege,

ich berichte über unseren Patienten, der sich 3 Monate nach Abschluss der ultrahypofraktionierten Strahlentherapie in unserer Sprechstunde vorstellte.

Der zuletzt im November 2021 bestimmte PSA-Wert betrug 4,18. Patient ist in altersentsprechend gutem Allgemeinzustand. Es besteht seit der Bestrahlung weiterhin eine Blasenentleerungsstörung mit einem IPSS-Score von 12. Patient nimmt Tamsulosin. Sonst keine Hinweise für radiogene Toxizität.

Digital-rektale Untersuchung: perianal reizlos. Prostatavergrößert, nicht konsistenzvermehrt. Kein Tastbefund. Kein Tumor tastbar. Kein Blut.

Denken wir Schritt für Schritt:

- Der PSA-Wert "4,18" befindet sich im Satz "Der zuletzt im November 2021 bestimmte PSA-Wert betrug 4,18".

- In unmittelbarer Nähe des PSA-Werts wurde mit dem Hinweis "November 2021" ein Datum gefunden, das gemäß den Regeln verwendet wird, ohne die PSA-Liste zu überprüfen.

- Es wird überprüft, ob ein vollständiges Datum (Tag, Monat, Jahr) vorhanden ist. Da nur Monat und Jahr angegeben sind, wird gemäß den Regeln der erste Tag des Monats angenommen.

- Das Datum "November 2021" wird als "01-11-2021" formatiert.

- Da das Datum bereits eindeutig identifiziert wurde, wird die Liste nicht weiter überprüft.

Antwort:

{

"FU3M_IsPSADetermined": "1",

"FU3M_PSADate": "01-11-2021",

"FU3M_PSALevel": "4.18"

}

2. Text:

Tumormarker PSA [ng/ml]: 4,738 (11/20); 4,498 (02/21); 6,921 (08/21); 1,43 (01/2022)

Sehr geehrter Herr Kollege,

wir berichten über Herrn [ANONYMIZED], der sich am 04.03.2022 in unserer Ambulanz 3 nach Abschluss der o. g. Strahlentherapie zur strahlentherapeutischen Nachschau wieder vorstellte.

Wir danken für die Mitgabe des aktuellen PSA-Verlaufs.

Aktueller PSA-Wert: 1,43 ng/ml. Eine antihormonelle Therapie erfolgt nicht.

Denken wir Schritt für Schritt:

- Der PSA-Wert "1,43 ng/ml" befindet sich in der "Sehr geehrter"-Einleitung.

- Es gibt in unmittelbarer Nähe des PSA-Wertes kein explizites Datum.

- Überprüfung der PSA-Liste: Der letzte Wert in der Liste ist "1,43 (01/2022)".

- Der Wert "1,43" aus der Liste stimmt mit "1,43 ng/ml" überein.

- Es wird überprüft, ob ein vollständiges Datum (Tag, Monat, Jahr) vorhanden ist. Da nur Monat und Jahr angegeben sind, wird gemäß den Regeln der erste Tag des Monats angenommen.

- Das Datum "01/2022" wird als "01-01-2022" formatiert.

Antwort:

{

"FU3M_IsPSADetermined": "1",

"FU3M_PSADate": "01-01-2022",

"FU3M_PSALevel": "1.43"

}

3. Text:

Tumormarker PSA [ng/ml]: 10,5 (02/20); 15,23 (02/23, UKT); 7,75 (04/23)

Sehr geehrter Herr Kollege,

wir berichten über Herrn [ANONYMIZED], der sich am 16.06.2023 in unserer Ambulanz 3 nach Abschluss der o. g. Strahlentherapie zur strahlentherapeutischen Nachschau wieder vorstellte.

Herr [ANONYMIZED] berichtete Wohlbefinden und gute Belastbarkeit. Stuhlgang und Wasserlassen bereiten keine Probleme. Die letzte Nachsorge erfolgte am 04.2023 Urologe und war unauffällig.

Aktueller PSA: 7,75 ng/ml

Denken wir Schritt für Schritt:

- Der PSA-Wert "7,75 ng/ml" befindet sich in der "Sehr geehrter"-Einleitung.

- Es gibt in unmittelbarer Nähe des PSA-Wertes kein explizites Datum.

- Überprüfung der PSA-Liste: Der letzte Wert in der Liste ist "7,75 (04/23)".

- Der Wert "7,75" aus der Liste stimmt mit "7,75 ng/ml" überein.

- Es wird überprüft, ob ein vollständiges Datum (Tag, Monat, Jahr) vorhanden ist. Da nur Monat und Jahr angegeben sind, wird gemäß den Regeln der erste Tag des Monats angenommen.

- Das Datum "04/2023" wird als "01-04-2023" formatiert.

Antwort:

{

"FU3M_IsPSADetermined": "1",

"FU3M_PSADate": "01-04-2023",

"FU3M_PSALevel": "7.75"

}

4. Text:

Tumormarker iPSA: 5,27µg/l, 21.04.2020 7,28µg/l PSA: 07/2020 0,13; 10/2020 0,17; 10.02.2021 0,22µg/l 23.04.2021: 0,27

Sehr geehrter Herr Kollege,

ich berichte über unseren gemeinsamen Patienten, Herr [ANONYMIZED]3 Monate nach Abschluss der Strahlentherapie in unserer Sprechstunde vorstellte.

Patient in sehr gutem Allgemeinzustand. Schmerzhafte Brustwarzen ohne Gynäkomastie unter Bicalutamid. Aktuelle PSA-Wert beträgt 0,05. Keine Nebenwirkungen hinsichtlich gastrointestinaler oder uro-genitaler Toxizität nach Strahlentherapie. Erektionsfähigkeit bereits vor Strahlentherapie erloschen.

Digital-rektale Untersuchung: Prostataloge leer, Schleimhaut glatt. Kein Blut. Perianale Haut reizlos.

Denken wir Schritt für Schritt:

- Der PSA-Wert "0,05" befindet sich in der "Sehr geehrter"-Einleitung.

- In unmittelbarer Nähe des PSA-Werts gibt es keine explizite Datumsangabe.

- Überprüfung der PSA-Liste: Der letzte Eintrag in der PSA-Liste ist "23.04.2021: 0,27", aber der Wert stimmt nicht mit dem aktuellen PSA-Wert "0,05" überein.

- Da kein passendes Datum in der Nähe oder in der Liste gefunden wird, wird das Standarddatum gemäß den Regeln zurückgegeben.

- Das Standarddatum "01-01-1901" wird gemäß den Formatierungsregeln verwendet.

Antwort:

{

"FU3M_IsPSADetermined": "1",

"FU3M_PSADate": "01-01-1901",

"FU3M_PSALevel": "0.05"

}

5. Text:

Tumormarker PSA [ng/ml]: 12,13 (06/17); 16,09 (07/17); 0,01 (02/18); 0,01 (05/18); 0,02 (09/18); 0,03 (05/19); 0,04 (08/19); 0,07 (02/20); 0,11 (08/20); 0,22 (02/21); 0,30 (06/21); 0,36 (09/21); 0,11 (03/22); 0,18 (06/22); 0,29 (09/22); 0,8 (01/23), <0,02 (07/23)

Sehr geehrter Herr Kollege,

wir berichten über Herrn [ANONYMIZED], der sich am 19.01.2024 in unserer Ambulanz nach Abschluss der o. g. Strahlentherapie zur strahlentherapeutischen Nachschau wieder vorstellte.

Herr [ANONYMIZED] berichtete Wohlbefinden und gute Belastbarkeit. Stuhlgang und Wasserlassen bereiten keine Probleme. Die letzte Nachsorge erfolgte am 12/2023 Ihnen und war unauffällig. Aktuell keine Gynäkomastie Zeichen. Bei bildmorphologischem Nachweis ossärer und lymphonodaler Metastasen eines vorbekannten Prostata-CA im Februar 2023 wurde im März 2023 eine Langzeit-ADT mit Trenantone und Apalutamid eingeleitet.

Aktueller PSA: <0,02 ng/ml

Denken wir Schritt für Schritt:

- Der PSA-Wert "<0,02 ng/ml" befindet sich in der "Sehr geehrter"-Einleitung.

- Es gibt in unmittelbarer Nähe des PSA-Wertes kein explizites Datum.

- Überprüfung der PSA-Liste nach "Tumormarker": Der letzte Wert in der Liste ist "<0,02 (07/23)".

- Der Wert "<0,02" aus der Liste stimmt mit "<0,02 ng/ml" überein.

- Es wird überprüft, ob ein vollständiges Datum (Tag, Monat, Jahr) vorhanden ist. Da nur Monat und Jahr angegeben sind, wird gemäß den Regeln der erste Tag des Monats angenommen.

- Das Datum "07/23" wird als "01-07-2023" formatiert.

- Der extrahierte PSA-Wert enthält das Zeichen "<", das entfernt wird, da der Wert numerisch gespeichert werden muss.

- Da ein passendes Datum aus der PSA-Liste gefunden wurde, ist keine weitere Überprüfung erforderlich.

Antwort:

{

"FU3M_IsPSADetermined": "1",

"FU3M_PSADate": "01-07-2023",

"FU3M_PSALevel": "0.02"

}

### Antwort Format:

{

"FU3M_IsPSADetermined": "<1 oder 0>",

"FU3M_PSADate": "<Datum im Format 'DD-MM-YYYY', '01-MM-YYYY' oder '01-01-1901'>",

"FU3M_PSALevel": "<PSA-Wert oder 'Unknown'>"

}

"""

},

{

"name": "FU3M_ECOG",

"prompt": """

### Query

Bitte extrahiere den ECOG-Status des Patienten aus dem Text und gib ausschließlich ein einziges JSON-Objekt auf Deutsch zurück. Zusätzliche Texte, Erklärungen oder mehrere JSON-Objekte sind nicht erlaubt.

**Vorprüfung – Wichtig:**

Bevor du beginnst: Prüfe, ob im Text **eine der folgenden Formulierungen** eindeutig vorkommt:

- „ECOG“

- „ECOG-Status“

- „ECOG I–IV“

- „ECOG Performance“

**Wenn keine dieser Formulierungen vorkommt**, beende die Aufgabe sofort und gib exakt dieses JSON zurück:

```json

{

"FU3M_ECOG_YN": "0",

"FU3M_ECOG_DATE": null,

"FU3M_ECOG": null

}

```

**Wenn eine dieser Formulierungen vorhanden ist**, fahre mit den folgenden Extraktionsregeln fort.

*Wichtig:** Alle Datumsangaben müssen im Format `DD-MM-YYYY` angegeben werden – z. B. `13-09-2023`.

**Andere Formate wie `13.09.2023`, `2023-09-13` oder ausgeschriebene Daten sind nicht erlaubt.**

### Schritte und Regeln:

1. **ECOG nur bei expliziter Nennung**

Der ECOG-Status darf **nur dann extrahiert werden**, wenn im Text **eindeutig** einer der folgenden Begriffe enthalten ist (Groß-/Kleinschreibung egal):

- „ECOG“

- „ECOG-Status“

- „ECOG I-IV“

**Wenn keiner dieser Begriffe vorkommt**, darf **kein ECOG-Wert extrahiert werden**, auch nicht basierend auf einer Beschreibung des Allgemeinzustands.

2. **Ungültige Begriffe (nie als ECOG interpretieren):**

- „CTCAE“, „CTC“, „RTOG“

- „WHO-Performance“, „Karnofsky“

- „Allgemeinzustand“, „Grad“, „pflegebedürftig“, „bettlägerig“, „60%“, „100“, „ECOG-like“

3. **Gültige ECOG-Werte (nur wenn direkt bei ECOG-Nennung genannt):**

- Nur die Werte „0“, „1“, „2“, „3“ oder „4“ (auch römisch I–IV)

- Keine Prozentsätze, Grade, freien Beschreibungen etc.

4. **Datumsregel (nur wenn ECOG erkannt wurde):**

- Wenn ein Datum **direkt beim ECOG** genannt ist → verwende dieses.

- Falls nicht vorhanden: Verwende das **Datum der Patientenvorstellung** – also ein **explizites, einzelnes Vorstellungsdatum** wie z. B.:

> „Die Patientin stellte sich am 13.09.2023 in unserer Ambulanz vor.“

- **Achtung:** Datumsangaben aus einem **Behandlungszeitraum** dürfen **nicht** verwendet werden. Beispiel (ungültig):

> „…der sich vom 04.08. bis zum 15.08.2023 in unserer ambulanten radioonkologischen Behandlung befand“

- Falls kein Vorstellungdatum vorhanden ist: Verwende das **Dokumentdatum**.

- Wenn **kein zulässiges Datum gefunden werden kann**, setze das Datum auf `"01-01-1901"`.

- Wenn **kein ECOG erkannt wurde**, setze das Datum auf `null`.

5. **Datumsformat:**

- Immer exakt **DD-MM-YYYY** (z. B. „04-09-2021“)

- Keine Punkte, keine ISO-Formate, kein Textdatum

- Unsichere Daten → `"01-01-1901"` (falls ECOG vorhanden) oder `null` (falls nicht)

6. **Antwortformatierung:**

- `"FU3M_ECOG_YN"` = `"1"`, wenn ein gültiger ECOG-Status extrahiert wurde

- `"FU3M_ECOG_YN"` = `"0"`, **wenn kein ECOG im Text erkannt wurde**

- `"FU3M_ECOG_DATE"` = Datum im Format `"DD-MM-YYYY"` oder `"01-01-1901"` oder `null`

- `"FU3M_ECOG"` = ECOG-Wert zwischen `"0"` und `"4"` als String oder `null`

---

### Denke Schritt für Schritt:

1. **Kommt im Text eine der zulässigen ECOG-Formulierungen vor?**

→ Wenn **Nein**, antworte sofort mit:

```json

{

"BASE_ECOG_YN": "0",

"BASE_ECOG_DATE": null,

"BASE_ECOG": null

}

```

→ Wenn **Ja**, fahre fort mit der Extraktion von Wert und Datum.

2. **Ist der ECOG-Wert gültig?** (Nur 0–4, ggf. römisch)

→ Wenn **ungültig**, ebenfalls wie oben antworten.

→ Wenn **gültig**, extrahiere Datum gemäß Regel 4.

3. **Welches Datum soll verwendet werden?**

- Zuerst: Gibt es ein **Datum direkt bei der ECOG-Angabe**? → Verwende dieses.

- Falls nicht: Suche nach einem **klaren Vorstellungstermin**, z. B.:

> „Die Patientin stellte sich am 14.03.2023 vor.“

- **Achtung:** Wenn im Text ein **Behandlungszeitraum** genannt wird, z. B.:

> „…der sich vom 04.08. bis zum 15.08.2023 in Behandlung befand“

→ **Dieser Zeitraum darf nicht als Datum verwendet werden.**

- Falls kein Vorstellungstermin vorhanden ist: Verwende das **Dokumentdatum**.

- Wenn kein gültiges Datum erkennbar ist → `"01-01-1901"` verwenden.

4. **Gib am Ende ausschließlich das JSON-Objekt im korrekten Format zurück.**

---

### Beispiele:

1. Text: "Der ECOG-Status beträgt 0"

Denken wir Schritt für Schritt:

- Der Text enthält die Formulierung „ECOG-Status beträgt 0“, was gültig ist.

- Kein Datum vorhanden → Standardwert verwenden

Antwort:

{

"FU3M_ECOG_YN": "1",

"FU3M_ECOG_DATE": "01-01-1901",

"FU3M_ECOG": "0"

}

---

2. Text: "ECOG III laut Verlaufsnotiz vom 12.01.2022."

Denken wir Schritt für Schritt:

- „ECOG III“ ist eine gültige Formulierung → Wert = 3

- Datum ist vorhanden → 12-01-2022

Antwort:

{

"FU3M_ECOG_YN": "1",

"FU3M_ECOG_DATE": "12-01-2022",

"FU3M_ECOG": "3"

}

---

3. Text: "Die Patientin ist bettlägerig, benötigt durchgehend Hilfe."

Denken wir Schritt für Schritt:

- Es wird kein „ECOG“, „ECOG-Status“ etc. erwähnt. Nur eine Beschreibung.

- Kein ECOG-Wert darf extrahiert werden.

Antwort:

{

"FU3M_ECOG_YN": "0",

"FU3M_ECOG_DATE": null,

"FU3M_ECOG": null

}

---

4. Text: "Der Patient war vollständig abhängig von fremder Hilfe (ECOG IV)."

Denken wir Schritt für Schritt:

- Die Formulierung „ECOG IV“ wird erkannt → Wert = 4

- Kein explizites Datum → Standardwert verwenden

Antwort:

{

"FU3M_ECOG_YN": "1",

"FU3M_ECOG_DATE": "01-01-1901",

"FU3M_ECOG": "4"

}

---

5. Text: "Karnofsky 60%, Allgemeinzustand eingeschränkt."

Denken wir Schritt für Schritt:

- Keine zulässige Formulierung wie „ECOG“ enthalten.

- Kein ECOG-Wert extrahierbar.

Antwort:

{

"FU3M_ECOG_YN": "0",

"FU3M_ECOG_DATE": null,

"FU3M_ECOG": null

}

---

### Antwortformat:

{

"FU3M_ECOG_YN": "<'1' oder '0'>",

"FU3M_ECOG_DATE": "<Datum im Format DD-MM-YYYY oder '01-01-1901' oder null>",

"FU3M_ECOG": "<Zahl zwischen '0' und '4' oder null>"

}

"""

}

]

## Supplementary Text S1 d) user_prompts_FU6M

user_prompts_FU6M = [

{

"name": "FU6M_KPSS",

"prompt": """

### Query:

Bitte extrahiere die KPSS-Informationen aus dem Text und gib ausschließlich ein einziges JSON-Objekt zurück. Zusätzliche Texte, Erklärungen oder mehrere JSON-Objekte sind nicht erlaubt.

Die KPSS-Informationen bestehen aus dem Karnofsky-Index (FU6M_KPSSValue), der den allgemeinen Gesundheitszustand eines Patienten in Prozent angibt, und dem zugehörigen Datum (FU6M_KPSSDate), das angibt, wann dieser Wert dokumentiert wurde. Falls keine KPSS-Informationen im Text vorhanden sind, wird dies entsprechend als "Unknown" gekennzeichnet.

### Schritte und Regeln:

1. **Suche nach dem Begriff "Karnofsky-Index" oder einer Variante wie "Karnofsky".**

- Falls **kein solcher Begriff im Text vorkommt**:

- Setze `"FU6M_IsKPSSDetermined"` auf `"0"`.

- Setze `"FU6M_KPSSValue"` auf `null`.

- Setze `"FU6M_KPSSDate"` auf `null`.

- **Beende die Extraktion an dieser Stelle.**

- **Es dürfen keine Werte geraten, geschätzt oder auf andere Weise abgeleitet werden.**

2. **Wenn "Karnofsky" gefunden wurde:**

- **Extrahiere den FU6M_KPSSValue:**

- Der Wert steht direkt hinter dem gefundenen Begriff.

- Falls ein Bereich wie "80 – 90 %" angegeben ist, wähle den **niedrigeren Wert** (hier: `"80"`).

- Falls **kein klarer Wert angegeben ist**, obwohl "Karnofsky" vorkommt:

- Setze `"F6M_KPSSValue"` auf `"Unknown"`.

- Setze `"FU6M_IsKPSSDetermined"` auf `"1"`.

- Setze `"FU6M_KPSSDate"` auf `null`.

- **Beende die Extraktion an dieser Stelle.**

- **Extrahiere das Datum FU6M_KPSSDate:**

- Suche im Abschnitt, der mit „Sehr geehrter“ beginnt, nach dem Ausdruck **„der sich am <Datum>“**.

- Falls ein solches Datum vorhanden ist, gib es im Format `"DD-MM-YYYY"` zurück.

- Falls kein Datum gefunden wird, verwende `"01-01-1901"`.

3. **Zusammenfassend gilt:**

- Nur wenn `"FU6M_IsKPSSDetermined"` den Wert `"1"` hat, dürfen `"FU6M_KPSSValue"` und `"FU6M_KPSSDate"` **einen gültigen Wert enthalten**.

- Wenn `"FU6M_IsKPSSDetermined"` den Wert `"0"` hat, müssen **beide anderen Felder zwingend `null` sein** – selbst wenn im Text ein Datum genannt wird.

---

### Formatierungsregeln für das Datum:

- Wenn das Datum Tag, Monat und Jahr enthält, gib es im Format `"DD-MM-YYYY"` zurück.

- Falls kein Datum gefunden wird, gib `"01-01-1901"` zurück.

- Falls kein KPSS-Wert extrahiert wurde, muss das Datum `null` sein.

---

### Beispiele:

1. Text:

Sehr geehrter Herr Kollege,

wir berichten über Herrn [ANONYMIZED], der sich am 17.10.2024 in unserer Ambulanz 3 nach Abschluss der o. g. Strahlentherapie zur strahlentherapeutischen Nachschau wieder vorstellte.

Herr [ANONYMIZED] berichtete mäßiges Wohlbefinden und eingeschränkte Belastbarkeit, dies sei aber auf Grund seiner kardialen Erkrankungen schon länger bekannt und konstant. Stuhlgang und Wasserlassen bereiten keine Probleme. Bisher sei keine ambulante Vorstellung beim Urologen erfolgt.

Aktueller PSA: 0,91 µg/l, IPSS 3

Untersuchungsbefund:

Karnofsky-Index 80 -90 %, Körpergewicht 90 kg.

Denken wir Schritt für Schritt:

- Der Begriff "Karnofsky-Index" ist vorhanden.

- Die Werte sind "80 - 90 %". Der niedrigere Wert ist "80".

- Das Datum steht hinter "der sich am" und lautet "17.10.2024".

- Das Datum wird ins Format "DD-MM-YYYY" konvertiert: "17-10-2024".

- Setze "FU6M_IsKPSSDetermined" auf 1.

Antwort:

{

"FU6M_IsKPSSDetermined": "1",

"FU6M_KPSSDate": "17-10-2024",

"FU6M_KPSSValue": "90"

}

2. Text:

Sehr geehrter Herr Kollege,

wir berichten über Herrn [ANONYMIZED], der sich am 16.06.2023 in unserer Ambulanz 3 nach Abschluss der o. g. Strahlentherapie zur strahlentherapeutischen Nachschau wieder vorstellte.

Herr [ANONYMIZED] berichtete Wohlbefinden und gute Belastbarkeit. Stuhlgang und Wasserlassen bereiten keine Probleme. Die letzte Nachsorge erfolgte am 04.2023 Urologe und war unauffällig.

Aktueller PSA: 7,75 ng/ml

Die antiandrogene Therapie mit einem GnRH-Analogon wird nicht durchgeführt.

Untersuchungsbefund:

Karnofsky-Index 100 %, Körpergewicht 74 kg.

Denken wir Schritt für Schritt:

- Der Begriff "Karnofsky-Index" wird im Abschnitt "Untersuchungsbefund" gefunden.

- Direkt hinter dem Begriff steht der Wert "100 %". Da es sich um einen eindeutigen Wert handelt und kein Zahlenbereich vorliegt, wird "100" als FU6M_KPSSValue übernommen.

- Anschließend wird im Abschnitt, nach dem Ausdruck "der sich am" gesucht, um das FU6M_KPSSDate zu bestimmen. Es wird der Text "der sich am 16.06.2023" identifiziert.

- Das Datum "16.06.2023" wird gemäß den Formatierungsregeln ins Format "DD-MM-YYYY" umgewandelt und ergibt "16-06-2023".

- Setze "FU6M_IsKPSSDetermined" auf 1.

Antwort:

{

"FU6M_IsKPSSDetermined": "1",

"FU6M_KPSSDate": "16-06-2023",

"FU6M_KPSSValue": "100"

}

3. Text:

Sehr geehrter Herr Kollege,

wir berichten über Herrn [ANONYMIZED] 3 Monate nach Abschluss der o.g. Strahlentherapie zur strahlentherapeutischen Nachschau wieder vorstellte.

Herr [ANONYMIZED] welche nach der OP bereits deutlich gebessert war und nach Bestrahlung nun wieder verschlechtert vorliegt. Vorlagen werden bei Terminen außerhalb genutzt. Der Beckenboden wird seit der Reha regelmäßig beübt. Die letzte uroonkologische Nachsorge war unauffällig. Ein nächster Termin ist für den 26.04.21 eingeplant.

Aktueller PSA: <0,0 (1/2021) unter ADT (seit 10/2020)

Untersuchungsbefund:

Karnofsky-Index 90 %, Körpergewicht 85,5 kg, konstant.

Denken wir Schritt für Schritt:

- Der Begriff "Karnofsky-Index" wird im Abschnitt "Untersuchungsbefund" gefunden.

- Direkt dahinter steht der Wert "90 %". Da es sich um einen eindeutigen Wert handelt, wird "90" als FU6M_KPSSValue extrahiert.

- Anschließend wird im Abschnitt, der mit "Sehr geehrter" beginnt, nach dem Ausdruck "der sich am" gesucht, um das Datum für FU6M_KPSSDate zu bestimmen.

- Da kein "der sich am"-Datum vorhanden ist und die Angabe "3 Monate nach Abschluss der o.g. Strahlentherapie" kein konkretes Datum liefert, wird gemäß den Regeln das Standarddatum "01-01-1901" verwendet.

- Da der Wert erfolgreich extrahiert wurde, wird FU6M_IsKPSSDetermined auf 1 gesetzt.

Antwort:

{

"FU6M_IsKPSSDetermined": "1",

"FU6M_KPSSDate": "01-01-1901",

"FU6M_KPSSValue": "90"

}

4. Text:

Wir berichten über Herrn [ANONYMIZED], der sich am 08.09.2023 in unserer Ambulanz 4 nach Abschluss der o. g. Strahlentherapie zur strahlentherapeutischen Nachschau wieder vorstellte.

Herr [ANONYMIZED] berichtete Wohlbefinden und gute Belastbarkeit. Stuhlgang und Wasserlassen bereiten keine Probleme. Die letzte Nachsorge erfolgte Ihnen und war unauffällig.

Aktueller PSA: <0,0006

Untersuchungsbefund:

Karnofsky-Index 100 %.

Denken wir Schritt für Schritt:

- Der Begriff "Karnofsky-Index" wird im Abschnitt "Untersuchungsbefund" gefunden.

- Der Wert direkt dahinter lautet "100 %". Da es sich um eine einzelne Zahl handelt, wird dieser Wert direkt als FU6M_KPSSValue übernommen.

- Anschließend wird im Abschnitt, der mit "Sehr geehrter" beginnt, nach dem Ausdruck "der sich am" gesucht, um das Datum für FU6M_KPSSDate zu bestimmen. In der Einleitung findet sich die Datumsangabe "08.09.2023".

- Es enthält Tag, Monat und Jahr und wird im Format "DD-MM-YYYY" übernommen: "08-09-2023".

- Setze "FU6M_IsKPSSDetermined" auf 1.

Antwort:

{

"FU6M_IsKPSSDetermined": "1",

"FU6M_KPSSDate": "08-09-2023",

"FU6M_KPSSValue": "100"

}

5. Text:

Sehr geehrter Herr Kollege,

wir berichten über Herrn [ANONYMIZED], der sich am 04.03.2022 in unserer Ambulanz 3 nach Abschluss der o. g. Strahlentherapie zur strahlentherapeutischen Nachschau wieder vorstellte.

Wir danken für die Mitgabe des aktuellen PSA-Verlaufs.

Aktueller PSA-Wert: 1,43 ng/ml. Eine antihormonelle Therapie erfolgt nicht.

Wir haben folgende Befunde erhoben:

Parameter: Wert (abzüglich Baseline bei CTC) Falls G3+Nebenwirkung:

Denken wir Schritt für Schritt:

- Im gesamten Text wird der Begriff "Karnofsky-Index" nicht gefunden.

- Da kein Wert für den Karnofsky-Index vorhanden ist, wird FU6M_KPSSValue gemäß den Regeln auf "Unknown" gesetzt.

- Da kein Wert gefunden wurde, erübrigt sich die Suche nach dem Datum.

- FU6M_KPSSDate wird daher auf das Standarddatum "01-01-1901" gesetzt.

- Da weder ein Wert noch ein Datum bestimmt werden konnte, wird FU6M_IsKPSSDetermined auf 0 gesetzt.

Antwort:

{

"FU6M_IsKPSSDetermined": "0",

"FU6M_KPSSDate": null,

"FU6M_KPSSValue": null

}

### Antwort Format:

{

"FU6M_IsKPSSDetermined": "<1 oder 0>",

"FU6M_KPSSDate": "<Datum im Format DD-MM-YYYY , '01-01-1901' oder null>",

"FU6M_KPSSValue": "<KPSS-Wert oder 'Unknown'>"

}

"""

},

{

"name": "FU6M_PSAList",

"prompt": """

### Query:

Bitte extrahiere den gesamten Abschnitt, der mit "Tumormarker" oder "PSA-Verlauf" beginnt, aus dem bereitgestellten Text und gib ausschließlich ein einziges JSON-Objekt zurück. Zusätzliche Texte, Erklärungen oder mehrere JSON-Objekte sind nicht erlaubt.

### Schritte und Regeln:

1. Suche im Text nach einem Abschnitt, der mit "Tumormarker" oder "PSA-Verlauf" beginnt.

2. Extrahiere alle Informationen, die direkt nach "Tumormarker" oder "PSA-Verlauf" folgen, einschließlich Werte, Daten oder Notizen.

3. Wenn kein Abschnitt mit "Tumormarker" oder "PSA-Verlauf" gefunden wird, gib "Unknown" zurück.

### Beispiele:

1. Text: "Tumormarker PSA [ng/ml]: 6,86 (06/22); 5,89 (09/22)"

Denken wir Schritt für Schritt:

- Der Abschnitt beginnt mit "Tumormarker PSA [ng/ml]".

- Extrahiere alle nachfolgenden Inhalte: "6,86 (06/22); 5,89 (09/22)".

Antwort:

{

"FU6M_PSAList": "Tumormarker PSA [ng/ml]: 6,86 (06/22); 5,89 (09/22)"

}

2. Text: "Tumormarker PSA (ng/ml): 06/16: 4,2, 11/16: 4,6; 06/17: 4,4; 11/19: 7,15; 12/19: 7,3 Aktuell: 05/20: 6,7"

Denken wir Schritt für Schritt:

- Der Abschnitt beginnt mit "Tumormarker PSA (ng/ml)".

- Extrahiere alle nachfolgenden Inhalte: "06/16: 4,2, 11/16: 4,6; 06/17: 4,4; 11/19: 7,15; 12/19: 7,3 Aktuell: 05/20: 6,7".

Antwort:

{

"FU6M_PSAList": "Tumormarker PSA (ng/ml): 06/16: 4,2, 11/16: 4,6; 06/17: 4,4; 11/19: 7,15; 12/19: 7,3 Aktuell: 05/20: 6,7"

}

3. Text: "Keine Erwähnung von Tumormarkern im Bericht."

Denken wir Schritt für Schritt:

- Es gibt keinen Abschnitt, der mit "Tumormarker" beginnt.

- Gib "Unknown" zurück.

Antwort:

{

"FU6M_PSAList": "Unknown"

}

4. Text: "PSA-Verlauf (ng/ml): 5,29 (03/23); 5,02 (05/23); 5,85 (10/23); 7,47 (01/24); 7,99 (04/24); 9,9 (06/24)"

Denken wir Schritt für Schritt:

- Der Abschnitt beginnt mit "PSA-Verlauf".

- Extrahiere alle nachfolgenden Inhalte: "5,29 (03/23); 5,02 (05/23); 5,85 (10/23); 7,47 (01/24); 7,99 (04/24); 9,9 (06/24)".

Antwort:

{

"FU6M_PSAList": "PSA-Verlauf (ng/ml): 5,29 (03/23); 5,02 (05/23); 5,85 (10/23); 7,47 (01/24); 7,99 (04/24); 9,9 (06/24)"

}

5. Text: "Tumormarker iPSA: 14,1 ng/ml"

Denken wir Schritt für Schritt:

- Der Abschnitt beginnt mit "Tumormarker".

- Extrahiere alle nachfolgenden Inhalte: "14,1 ng/ml".

Antwort:

{

"FU6M_PSAList": "Tumormarker iPSA: 14,1 ng/ml"

}

### Antwort Format:

{

"FU6M_PSAList": "<Extrahierter Abschnitt oder 'Unknown'>"

}

"""

},

{

"name": "FU6M_PSA",

"prompt": """

### Query:

Extrahiere den PSA-Wert (Prostata-spezifisches Antigen) und das zugehörige Datum aus dem bereitgestellten Text. Die Werte befinden sich entweder in der PSA-Liste am Anfang des Dokuments oder innerhalb der "Sehr geehrter"-Einleitung und am Ende des Textes. Gib ausschließlich ein einziges JSON-Objekt zurück. Zusätzliche Texte, Erklärungen oder mehrere Objekte sind nicht erlaubt.

Der extrahierte Wert FU6M_PSALevel bezieht sich auf den PSA-Wert der Nachsorgeuntersuchung und spiegelt den aktuellen PSA-Status des Patienten wider.

### Schritte und Regeln:

1. Suche in der "Sehr geehrter"-Einleitung oder am Ende des Textes nach einem PSA-Wert.

- Relevante Begriffe zur Identifikation des richtigen Wertes sind: "PSA-Wertes", "Aktueller PSA", "Aktueller PSA-Wert", "PSA", "PSA Wert".

- Der PSA-Wert ist die Zahl, die in unmittelbarer Nähe dieser Begriffe steht.

2. Bestimme den PSA-Wert:

- Extrahiere die Zahl, die direkt neben den oben genannten Begriffen steht.

- Falls mehrere PSA-Werte im selben Abschnitt vorkommen, wähle den aktuellen Nachsorge-PSA-Wert anhand der Position und Formulierung.

- Falls kein PSA-Wert in der "Sehr geehrter"-Einleitung oder am Textende gefunden wird, überprüfe die PSA-Liste am Dokumentanfang.

- Wähle den letzten PSA-Wert aus der Liste am Dokumentanfang.

3. Bestimme das zugehörige Datum:

- Suche nach einem Datum direkt vor oder nach dem gefundenen PSA-Wert.

- Falls kein solches Datum vorhanden ist, überprüfe die PSA-Liste am Dokumentanfang:

- Die PSA-Liste enthält PSA-Werte aus verschiedenen Zeitpunkten, getrennt durch Kommas oder Semikolons.

- Jede Einheit in der PSA-Liste besteht aus einem PSA-Wert und möglicherweise einem zugehörigen Testdatum.

- Falls der letzte PSA-Wert aus der Liste mit dem extrahierten PSA-Wert übereinstimmt, übernehme dessen Datum.

- Falls kein übereinstimmender PSA-Wert in der Liste gefunden wird, überprüfe, ob nach "der sich am" ein Datum steht. Falls ja, verwende dieses Datum.

- Falls kein Datum nach "der sich am" gefunden wird, setze das Datum auf "01-01-1901".

4. Falls in der "Sehr geehrter"-Einleitung oder am Ende des Textes kein PSA-Wert gefunden wird:

- Überprüfe den letzten PSA-Wert in der PSA-Liste am Dokumentanfang.

- Wähle den letzten PSA-Wert aus der Liste.

- Falls ein Datum vorhanden ist, verwende dieses Datum.

- Falls kein Datum verfügbar ist, überprüfe, ob nach "der sich am" ein Datum steht. Falls ja, verwende dieses.

- Falls kein Datum nach "der sich am" gefunden wird, gib "01-01-1901" zurück.

5. Bestimme FU6M_IsPSADetermined:

- Wenn ein PSA-Wert gefunden wird, setze "FU6M_IsPSADetermined": "1".

- Wenn kein PSA-Wert gefunden wird, setze "FU6M_IsPSADetermined": "0", "FU6M_PSADate": "01-01-1901", "FU6M_PSALevel": "Unknown".

### Formatierungsregeln für das Datum:

- Falls ein Datum mit Tag, Monat und Jahr vorhanden ist, gib es im Format "DD-MM-YYYY" zurück.

- Falls nur Monat und Jahr angegeben sind (z. B. "MM/YY"), interpretiere es als den ersten Tag des Monats und gib das Datum im Format "01-MM-YYYY" zurück.

- Falls nur das Jahr vorhanden ist, interpretiere es als "01-01-YYYY".

- Falls kein Datum gefunden wird, gib "01-01-1901" zurück.

### Beispiele:

1. Text:

Tumormarker PSA [ng/ml];: 5,8 (07/21)

Sehr geehrter Herr Kollege,

ich berichte über unseren Patienten, der sich 3 Monate nach Abschluss der ultrahypofraktionierten Strahlentherapie in unserer Sprechstunde vorstellte.

Der zuletzt im November 2021 bestimmte PSA-Wert betrug 4,18. Patient ist in altersentsprechend gutem Allgemeinzustand. Es besteht seit der Bestrahlung weiterhin eine Blasenentleerungsstörung mit einem IPSS-Score von 12. Patient nimmt Tamsulosin. Sonst keine Hinweise für radiogene Toxizität.

Digital-rektale Untersuchung: perianal reizlos. Prostatavergrößert, nicht konsistenzvermehrt. Kein Tastbefund. Kein Tumor tastbar. Kein Blut.

Denken wir Schritt für Schritt:

- Der PSA-Wert "4,18" befindet sich im Satz "Der zuletzt im November 2021 bestimmte PSA-Wert betrug 4,18".

- In unmittelbarer Nähe des PSA-Werts wurde mit dem Hinweis "November 2021" ein Datum gefunden, das gemäß den Regeln verwendet wird, ohne die PSA-Liste zu überprüfen.

- Es wird überprüft, ob ein vollständiges Datum (Tag, Monat, Jahr) vorhanden ist. Da nur Monat und Jahr angegeben sind, wird gemäß den Regeln der erste Tag des Monats angenommen.

- Das Datum "November 2021" wird als "01-11-2021" formatiert.

- Da das Datum bereits eindeutig identifiziert wurde, wird die Liste nicht weiter überprüft.

Antwort:

{

"FU6M_IsPSADetermined": "1",

"FU6M_PSADate": "01-11-2021",

"FU6M_PSALevel": "4.18"

}

2. Text:

Tumormarker PSA [ng/ml]: 4,738 (11/20); 4,498 (02/21); 6,921 (08/21); 1,43 (01/2022)

Sehr geehrter Herr Kollege,

wir berichten über Herrn [ANONYMIZED], der sich am 04.03.2022 in unserer Ambulanz 3 nach Abschluss der o. g. Strahlentherapie zur strahlentherapeutischen Nachschau wieder vorstellte.

Wir danken für die Mitgabe des aktuellen PSA-Verlaufs.

Aktueller PSA-Wert: 1,43 ng/ml. Eine antihormonelle Therapie erfolgt nicht.

Denken wir Schritt für Schritt:

- Der PSA-Wert "1,43 ng/ml" befindet sich in der "Sehr geehrter"-Einleitung.

- Es gibt in unmittelbarer Nähe des PSA-Wertes kein explizites Datum.

- Überprüfung der PSA-Liste: Der letzte Wert in der Liste ist "1,43 (01/2022)".

- Der Wert "1,43" aus der Liste stimmt mit "1,43 ng/ml" überein.

- Es wird überprüft, ob ein vollständiges Datum (Tag, Monat, Jahr) vorhanden ist. Da nur Monat und Jahr angegeben sind, wird gemäß den Regeln der erste Tag des Monats angenommen.

- Das Datum "01/2022" wird als "01-01-2022" formatiert.

Antwort:

{

"FU6M_IsPSADetermined": "1",

"FU6M_PSADate": "01-01-2022",

"FU6M_PSALevel": "1.43"

}

3. Text:

Tumormarker PSA [ng/ml]: 10,5 (02/20); 15,23 (02/23, UKT); 7,75 (04/23)

Sehr geehrter Herr Kollege,

wir berichten über Herrn [ANONYMIZED], der sich am 16.06.2023 in unserer Ambulanz 3 nach Abschluss der o. g. Strahlentherapie zur strahlentherapeutischen Nachschau wieder vorstellte.

Herr [ANONYMIZED] berichtete Wohlbefinden und gute Belastbarkeit. Stuhlgang und Wasserlassen bereiten keine Probleme. Die letzte Nachsorge erfolgte am 04.2023 Urologe und war unauffällig.

Aktueller PSA: 7,75 ng/ml

Denken wir Schritt für Schritt:

- Der PSA-Wert "7,75 ng/ml" befindet sich in der "Sehr geehrter"-Einleitung.

- Es gibt in unmittelbarer Nähe des PSA-Wertes kein explizites Datum.

- Überprüfung der PSA-Liste: Der letzte Wert in der Liste ist "7,75 (04/23)".

- Der Wert "7,75" aus der Liste stimmt mit "7,75 ng/ml" überein.

- Es wird überprüft, ob ein vollständiges Datum (Tag, Monat, Jahr) vorhanden ist. Da nur Monat und Jahr angegeben sind, wird gemäß den Regeln der erste Tag des Monats angenommen.

- Das Datum "04/2023" wird als "01-04-2023" formatiert.

Antwort:

{

"FU6M_IsPSADetermined": "1",

"FU6M_PSADate": "01-04-2023",

"FU6M_PSALevel": "7.75"

}

4. Text:

Tumormarker iPSA: 5,27µg/l, 21.04.2020 7,28µg/l PSA: 07/2020 0,13; 10/2020 0,17; 10.02.2021 0,22µg/l 23.04.2021: 0,27

Sehr geehrter Herr Kollege,

ich berichte über unseren gemeinsamen Patienten, Herr [ANONYMIZED]3 Monate nach Abschluss der Strahlentherapie in unserer Sprechstunde vorstellte.

Patient in sehr gutem Allgemeinzustand. Schmerzhafte Brustwarzen ohne Gynäkomastie unter Bicalutamid. Aktuelle PSA-Wert beträgt 0,05. Keine Nebenwirkungen hinsichtlich gastrointestinaler oder uro-genitaler Toxizität nach Strahlentherapie. Erektionsfähigkeit bereits vor Strahlentherapie erloschen.

Digital-rektale Untersuchung: Prostataloge leer, Schleimhaut glatt. Kein Blut. Perianale Haut reizlos.

Denken wir Schritt für Schritt:

- Der PSA-Wert "0,05" befindet sich in der "Sehr geehrter"-Einleitung.

- In unmittelbarer Nähe des PSA-Werts gibt es keine explizite Datumsangabe.

- Überprüfung der PSA-Liste: Der letzte Eintrag in der PSA-Liste ist "23.04.2021: 0,27", aber der Wert stimmt nicht mit dem aktuellen PSA-Wert "0,05" überein.

- Da kein passendes Datum in der Nähe oder in der Liste gefunden wird, wird das Standarddatum gemäß den Regeln zurückgegeben.

- Das Standarddatum "01-01-1901" wird gemäß den Formatierungsregeln verwendet.

Antwort:

{

"FU6M_IsPSADetermined": "1",

"FU6M_PSADate": "01-01-1901",

"FU6M_PSALevel": "0.05"

}

5. Text:

Tumormarker PSA [ng/ml]: 12,13 (06/17); 16,09 (07/17); 0,01 (02/18); 0,01 (05/18); 0,02 (09/18); 0,03 (05/19); 0,04 (08/19); 0,07 (02/20); 0,11 (08/20); 0,22 (02/21); 0,30 (06/21); 0,36 (09/21); 0,11 (03/22); 0,18 (06/22); 0,29 (09/22); 0,8 (01/23), <0,02 (07/23)

Sehr geehrter Herr Kollege ,

wir berichten über Herrn [ANONYMIZED], der sich am 19.01.2024 in unserer Ambulanz nach Abschluss der o. g. Strahlentherapie zur strahlentherapeutischen Nachschau wieder vorstellte.

Herr [ANONYMIZED] berichtete Wohlbefinden und gute Belastbarkeit. Stuhlgang und Wasserlassen bereiten keine Probleme. Die letzte Nachsorge erfolgte am 12/2023 Ihnen und war unauffällig. Aktuell keine Gynäkomastie Zeichen. Bei bildmorphologischem Nachweis ossärer und lymphonodaler Metastasen eines vorbekannten Prostata-CA im Februar 2023 wurde im März 2023 eine Langzeit-ADT mit Trenantone und Apalutamid eingeleitet.

Aktueller PSA: <0,02 ng/ml

Denken wir Schritt für Schritt:

- Der PSA-Wert "<0,02 ng/ml" befindet sich in der "Sehr geehrter"-Einleitung.

- Es gibt in unmittelbarer Nähe des PSA-Wertes kein explizites Datum.

- Überprüfung der PSA-Liste nach "Tumormarker": Der letzte Wert in der Liste ist "<0,02 (07/23)".

- Der Wert "<0,02" aus der Liste stimmt mit "<0,02 ng/ml" überein.

- Es wird überprüft, ob ein vollständiges Datum (Tag, Monat, Jahr) vorhanden ist. Da nur Monat und Jahr angegeben sind, wird gemäß den Regeln der erste Tag des Monats angenommen.

- Das Datum "07/23" wird als "01-07-2023" formatiert.

- Der extrahierte PSA-Wert enthält das Zeichen "<", das entfernt wird, da der Wert numerisch gespeichert werden muss.

- Da ein passendes Datum aus der PSA-Liste gefunden wurde, ist keine weitere Überprüfung erforderlich.

Antwort:

{

"FU6M_IsPSADetermined": "1",

"FU6M_PSADate": "01-07-2023",

"FU6M_PSALevel": "0.02"

}

### Antwort Format:

{

"FU6M_IsPSADetermined": "<1 oder 0>",

"FU6M_PSADate": "<Datum im Format 'DD-MM-YYYY', '01-MM-YYYY' oder '01-01-1901'>",

"FU6M_PSALevel": "<PSA-Wert oder 'Unknown'>"

}

"""

},

{

"name": "FU6M_ECOG",

"prompt": """

### Query

Bitte extrahiere den ECOG-Status des Patienten aus dem Text und gib ausschließlich ein einziges JSON-Objekt auf Deutsch zurück. Zusätzliche Texte, Erklärungen oder mehrere JSON-Objekte sind nicht erlaubt.

**Vorprüfung - Wichtig:**

Bevor du beginnst: Prüfe, ob im Text **eine der folgenden Formulierungen** eindeutig vorkommt:

- „ECOG“

- „ECOG-Status“

- „ECOG I-IV“

**Wenn keine dieser Formulierungen vorkommt**, beende die Aufgabe sofort und gib exakt dieses JSON zurück:

```json

{

"FU6M_ECOG_YN": "0",

"FU6M_ECOG_DATE": null,

"FU6M_ECOG": null

}

```

**Wenn eine dieser Formulierungen vorhanden ist**, fahre mit den folgenden Extraktionsregeln fort.

*Wichtig:** Alle Datumsangaben müssen im Format `DD-MM-YYYY` angegeben werden – z. B. `13-09-2023`.

**Andere Formate wie `13.09.2023`, `2023-09-13` oder ausgeschriebene Daten sind nicht erlaubt.**

### Schritte und Regeln:

1. **ECOG nur bei expliziter Nennung**

Der ECOG-Status darf **nur dann extrahiert werden**, wenn im Text **eindeutig** einer der folgenden Begriffe enthalten ist (Groß-/Kleinschreibung egal):

- „ECOG“

- „ECOG-Status“

- „ECOG I-IV“

**Wenn keiner dieser Begriffe vorkommt**, darf **kein ECOG-Wert extrahiert werden**, auch nicht basierend auf einer Beschreibung des Allgemeinzustands.

2. **Ungültige Begriffe (nie als ECOG interpretieren):**

- „CTCAE“, „CTC“, „RTOG“

- „WHO-Performance“, „Karnofsky“

- „Allgemeinzustand“, „Grad“, „pflegebedürftig“, „bettlägerig“, „60%“, „100“, „ECOG-like“

3. **Gültige ECOG-Werte (nur wenn direkt bei ECOG-Nennung genannt):**

- Nur die Werte „0“, „1“, „2“, „3“ oder „4“ (auch römisch I–IV)

- Keine Prozentsätze, Grade, freien Beschreibungen etc.

4. **Datumsregel (nur wenn ECOG erkannt wurde):**

- Wenn ein Datum **direkt beim ECOG** genannt ist → verwende dieses.

- Falls nicht vorhanden: Verwende das **Datum der Patientenvorstellung** – also ein **explizites, einzelnes Vorstellungsdatum** wie z. B.:

> „Die Patientin stellte sich am 13.09.2023 in unserer Ambulanz vor.“

- **Achtung:** Datumsangaben aus einem **Behandlungszeitraum** dürfen **nicht** verwendet werden. Beispiel (ungültig):

> „…der sich vom 04.08. bis zum 15.08.2023 in unserer ambulanten radioonkologischen Behandlung befand“

- Falls kein Vorstellungdatum vorhanden ist: Verwende das **Dokumentdatum**.

- Wenn **kein zulässiges Datum gefunden werden kann**, setze das Datum auf `"01-01-1901"`.

- Wenn **kein ECOG erkannt wurde**, setze das Datum auf `null`.

5. **Datumsformat:**

- Immer exakt **DD-MM-YYYY** (z. B. „04-09-2021“)

- Keine Punkte, keine ISO-Formate, kein Textdatum

- Unsichere Daten → `"01-01-1901"` (falls ECOG vorhanden) oder `null` (falls nicht)

6. **Antwortformatierung:**

- `"FU6M_ECOG_YN"` = `"1"`, wenn ein gültiger ECOG-Status extrahiert wurde

- `"FU6M_ECOG_YN"` = `"0"`, **wenn kein ECOG im Text erkannt wurde**

- `"FU6M_ECOG_DATE"` = Datum im Format `"DD-MM-YYYY"` oder `"01-01-1901"` oder `null`

- `"FU6M_ECOG"` = ECOG-Wert zwischen `"0"` und `"4"` als String oder `null`

---

### Denke Schritt für Schritt:

1. **Kommt im Text eine der zulässigen ECOG-Formulierungen vor?**

→ Wenn **Nein**, antworte sofort mit:

```json

{

"BASE_ECOG_YN": "0",

"BASE_ECOG_DATE": null,

"BASE_ECOG": null

}

```

→ Wenn **Ja**, fahre fort mit der Extraktion von Wert und Datum.

2. **Ist der ECOG-Wert gültig?** (Nur 0–4, ggf. römisch)

→ Wenn **ungültig**, ebenfalls wie oben antworten.

→ Wenn **gültig**, extrahiere Datum gemäß Regel 4.

3. **Welches Datum soll verwendet werden?**

- Zuerst: Gibt es ein **Datum direkt bei der ECOG-Angabe**? → Verwende dieses.

- Falls nicht: Suche nach einem **klaren Vorstellungstermin**, z. B.:

> „Die Patientin stellte sich am 14.03.2023 vor.“

- **Achtung:** Wenn im Text ein **Behandlungszeitraum** genannt wird, z. B.:

> „…der sich vom 04.08. bis zum 15.08.2023 in Behandlung befand“

→ **Dieser Zeitraum darf nicht als Datum verwendet werden.**

- Falls kein Vorstellungstermin vorhanden ist: Verwende das **Dokumentdatum**.

- Wenn kein gültiges Datum erkennbar ist → `"01-01-1901"` verwenden.

4. **Gib am Ende ausschließlich das JSON-Objekt im korrekten Format zurück.**

---

### Beispiele:

1. Text: "Der ECOG-Status beträgt 0"

Denken wir Schritt für Schritt:

- Der Text enthält die Formulierung „ECOG-Status beträgt 0“, was gültig ist.

- Kein Datum vorhanden → Standardwert verwenden

Antwort:

{

"FU6M_ECOG_YN": "1",

"FU6M_ECOG_DATE": "01-01-1901",

"FU6M_ECOG": "0"

}

---

2. Text: "ECOG III laut Verlaufsnotiz vom 12.01.2022."

Denken wir Schritt für Schritt:

- „ECOG III“ ist eine gültige Formulierung → Wert = 3

- Datum ist vorhanden → 12-01-2022

Antwort:

{

"FU6M_ECOG_YN": "1",

"FU6M_ECOG_DATE": "12-01-2022",

"FU6M_ECOG": "3"

}

---

3. Text: "Die Patientin ist bettlägerig, benötigt durchgehend Hilfe."

Denken wir Schritt für Schritt:

- Es wird kein „ECOG“, „ECOG-Status“ etc. erwähnt. Nur eine Beschreibung.

- Kein ECOG-Wert darf extrahiert werden.

Antwort:

{

"FU6M_ECOG_YN": "0",

"FU6M_ECOG_DATE": null,

"FU6M_ECOG": null

}

---

4. Text: "Der Patient war vollständig abhängig von fremder Hilfe (ECOG IV)."

Denken wir Schritt für Schritt:

- Die Formulierung „ECOG IV“ wird erkannt → Wert = 4

- Kein explizites Datum → Standardwert verwenden

Antwort:

{

"FU6M_ECOG_YN": "1",

"FU6M_ECOG_DATE": "01-01-1901",

"FU6M_ECOG": "4"

}

---

5. Text: "Karnofsky 60%, Allgemeinzustand eingeschränkt."

Denken wir Schritt für Schritt:

- Keine zulässige Formulierung wie „ECOG“ enthalten.

- Kein ECOG-Wert extrahierbar.

Antwort:

{

"FU6M_ECOG_YN": "0",

"FU6M_ECOG_DATE": null,

"FU6M_ECOG": null

}

---

### Antwortformat:

{

"FU6M_ECOG_YN": "<'1' oder '0'>",

"FU6M_ECOG_DATE": "<Datum im Format DD-MM-YYYY oder '01-01-1901' oder null>",

"FU6M_ECOG": "<Zahl zwischen '0' und '4' oder null>"

}

"""

}

]

## Supplementary Text S1 d) user_prompts_FU12M

user_prompts_FU12M = [

{

"name": "FU12M_KPSS",

"prompt": """

### Query:

Bitte extrahiere die KPSS-Informationen aus dem Text und gib ausschließlich ein einziges JSON-Objekt zurück. Zusätzliche Texte, Erklärungen oder mehrere JSON-Objekte sind nicht erlaubt.

Die KPSS-Informationen bestehen aus dem Karnofsky-Index (FU12M_KPSSValue), der den allgemeinen Gesundheitszustand eines Patienten in Prozent angibt, und dem zugehörigen Datum (FU12M_KPSSDate), das angibt, wann dieser Wert dokumentiert wurde. Falls keine KPSS-Informationen im Text vorhanden sind, wird dies entsprechend als "Unknown" gekennzeichnet.

### Schritte und Regeln:

1. **Suche nach dem Begriff "Karnofsky-Index" oder einer Variante wie "Karnofsky".**

- Falls **kein solcher Begriff im Text vorkommt**:

- Setze `"FU12M_IsKPSSDetermined"` auf `"0"`.

- Setze `"FU12M_KPSSValue"` auf `null`.

- Setze `"FU12M_KPSSDate"` auf `null`.

- **Beende die Extraktion an dieser Stelle.**

- **Es dürfen keine Werte geraten, geschätzt oder auf andere Weise abgeleitet werden.**

2. **Wenn "Karnofsky" gefunden wurde:**

- **Extrahiere den FU12M_KPSSValue:**

- Der Wert steht direkt hinter dem gefundenen Begriff.

- Falls ein Bereich wie "80 – 90 %" angegeben ist, wähle den **niedrigeren Wert** (hier: `"80"`).

- Falls **kein klarer Wert angegeben ist**, obwohl "Karnofsky" vorkommt:

- Setze `"FU12M_KPSSValue"` auf `"Unknown"`.

- Setze `"FU12M_IsKPSSDetermined"` auf `"1"`.

- Setze `"FU123M_KPSSDate"` auf `null`.

- **Beende die Extraktion an dieser Stelle.**

- **Extrahiere das Datum FU12M_KPSSDate:**

- Suche im Abschnitt, der mit „Sehr geehrter“ beginnt, nach dem Ausdruck **„der sich am <Datum>“**.

- Falls ein solches Datum vorhanden ist, gib es im Format `"DD-MM-YYYY"` zurück.

- Falls kein Datum gefunden wird, verwende `"01-01-1901"`.

3. **Zusammenfassend gilt:**

- Nur wenn `"FU12M_IsKPSSDetermined"` den Wert `"1"` hat, dürfen `"FU12M_KPSSValue"` und `"FU12M_KPSSDate"` **einen gültigen Wert enthalten**.

- Wenn `"FU12M_IsKPSSDetermined"` den Wert `"0"` hat, müssen **beide anderen Felder zwingend `null` sein** – selbst wenn im Text ein Datum genannt wird.

---

### Formatierungsregeln für das Datum:

- Wenn das Datum Tag, Monat und Jahr enthält, gib es im Format `"DD-MM-YYYY"` zurück.

- Falls kein Datum gefunden wird, gib `"01-01-1901"` zurück.

- Falls kein KPSS-Wert extrahiert wurde, muss das Datum `null` sein.

---

### Beispiele:

1. Text:

Sehr geehrter Herr Kollege,

wir berichten über Herrn [ANONYMIZED], der sich am 17.10.2024 in unserer Ambulanz 3 nach Abschluss der o. g. Strahlentherapie zur strahlentherapeutischen Nachschau wieder vorstellte.

Herr [ANONYMIZED] berichtete mäßiges Wohlbefinden und eingeschränkte Belastbarkeit, dies sei aber auf Grund seiner kardialen Erkrankungen schon länger bekannt und konstant. Stuhlgang und Wasserlassen bereiten keine Probleme. Bisher sei keine ambulante Vorstellung beim Urologen erfolgt.

Aktueller PSA: 0,91 µg/l, IPSS 3

Untersuchungsbefund:

Karnofsky-Index 80 -90 %, Körpergewicht 90 kg.

Denken wir Schritt für Schritt:

- Der Begriff "Karnofsky-Index" ist vorhanden.

- Die Werte sind "80 - 90 %". Der niedrigere Wert ist "80".

- Das Datum steht hinter "der sich am" und lautet "17.10.2024".

- Das Datum wird ins Format "DD-MM-YYYY" konvertiert: "17-10-2024".

- Setze "FU12M_IsKPSSDetermined" auf 1.

Antwort:

{

"FU12M_IsKPSSDetermined": "1",

"FU12M_KPSSDate": "17-10-2024",

"FU12M_KPSSValue": "90"

}

2. Text:

Sehr geehrter Herr Kollege,

wir berichten über Herrn [ANONYMIZED], der sich am 16.06.2023 in unserer Ambulanz 3 nach Abschluss der o. g. Strahlentherapie zur strahlentherapeutischen Nachschau wieder vorstellte.

Herr [ANONYMIZED] berichtete Wohlbefinden und gute Belastbarkeit. Stuhlgang und Wasserlassen bereiten keine Probleme. Die letzte Nachsorge erfolgte am 04.2023 Urologe und war unauffällig.

Aktueller PSA: 7,75 ng/ml

Die antiandrogene Therapie mit einem GnRH-Analogon wird nicht durchgeführt.

Untersuchungsbefund:

Karnofsky-Index 100 %, Körpergewicht 74 kg.

Denken wir Schritt für Schritt:

- Der Begriff "Karnofsky-Index" wird im Abschnitt "Untersuchungsbefund" gefunden.

- Direkt hinter dem Begriff steht der Wert "100 %". Da es sich um einen eindeutigen Wert handelt und kein Zahlenbereich vorliegt, wird "100" als FU12M_KPSSValue übernommen.

- Anschließend wird im Abschnitt, nach dem Ausdruck "der sich am" gesucht, um das FU12M_KPSSDate zu bestimmen. Es wird der Text "der sich am 16.06.2023" identifiziert.

- Das Datum "16.06.2023" wird gemäß den Formatierungsregeln ins Format "DD-MM-YYYY" umgewandelt und ergibt "16-06-2023".

- Setze "FU12M_IsKPSSDetermined" auf 1.

Antwort:

{

"FU12M_IsKPSSDetermined": "1",

"FU12M_KPSSDate": "16-06-2023",

"FU12M_KPSSValue": "100"

}

3. Text:

Sehr geehrter Herr Kollege,

wir berichten über Herrn [ANONYMIZED] 3 Monate nach Abschluss der o.g. Strahlentherapie zur strahlentherapeutischen Nachschau wieder vorstellte.

Herr [ANONYMIZED] welche nach der OP bereits deutlich gebessert war und nach Bestrahlung nun wieder verschlechtert vorliegt. Vorlagen werden bei Terminen außerhalb genutzt. Der Beckenboden wird seit der Reha regelmäßig beübt. Die letzte uroonkologische Nachsorge war unauffällig. Ein nächster Termin ist für den 26.04.21 eingeplant.

Aktueller PSA: <0,0 (1/2021) unter ADT (seit 10/2020)

Untersuchungsbefund:

Karnofsky-Index 90 %, Körpergewicht 85,5 kg, konstant.

Denken wir Schritt für Schritt:

- Der Begriff "Karnofsky-Index" wird im Abschnitt "Untersuchungsbefund" gefunden.

- Direkt dahinter steht der Wert "90 %". Da es sich um einen eindeutigen Wert handelt, wird "90" als FU12M_KPSSValue extrahiert.

- Anschließend wird im Abschnitt, der mit "Sehr geehrter" beginnt, nach dem Ausdruck "der sich am" gesucht, um das Datum für FU12M_KPSSDate zu bestimmen.

- Da kein "der sich am"-Datum vorhanden ist und die Angabe "3 Monate nach Abschluss der o.g. Strahlentherapie" kein konkretes Datum liefert, wird gemäß den Regeln das Standarddatum "01-01-1901" verwendet.

- Da der Wert erfolgreich extrahiert wurde, wird FU12M_IsKPSSDetermined auf 1 gesetzt.

Antwort:

{

"FU12M_IsKPSSDetermined": "1",

"FU12M_KPSSDate": "01-01-1901",

"FU12M_KPSSValue": "90"

}

4. Text:

Wir berichten über Herrn [ANONYMIZED], der sich am 08.09.2023 in unserer Ambulanz 4 nach Abschluss der o. g. Strahlentherapie zur strahlentherapeutischen Nachschau wieder vorstellte.

Herr [ANONYMIZED] berichtete Wohlbefinden und gute Belastbarkeit. Stuhlgang und Wasserlassen bereiten keine Probleme. Die letzte Nachsorge erfolgte Ihnen und war unauffällig.

Aktueller PSA: <0,0006

Untersuchungsbefund:

Karnofsky-Index 100 %.

Denken wir Schritt für Schritt:

- Der Begriff "Karnofsky-Index" wird im Abschnitt "Untersuchungsbefund" gefunden.

- Der Wert direkt dahinter lautet "100 %". Da es sich um eine einzelne Zahl handelt, wird dieser Wert direkt als FU12M_KPSSValue übernommen.

- Anschließend wird im Abschnitt, der mit "Sehr geehrter" beginnt, nach dem Ausdruck "der sich am" gesucht, um das Datum für FU12M_KPSSDate zu bestimmen. In der Einleitung findet sich die Datumsangabe "08.09.2023".

- Es enthält Tag, Monat und Jahr und wird im Format "DD-MM-YYYY" übernommen: "08-09-2023".

- Setze "FU12M_IsKPSSDetermined" auf 1.

Antwort:

{

"FU12M_IsKPSSDetermined": "1",

"FU12M_KPSSDate": "08-09-2023",

"FU12M_KPSSValue": "100"

}

5. Text:

Sehr geehrter Herr Kollege,

wir berichten über Herrn [ANONYMIZED], der sich am 04.03.2022 in unserer Ambulanz 3 nach Abschluss der o. g. Strahlentherapie zur strahlentherapeutischen Nachschau wieder vorstellte.

Wir danken für die Mitgabe des aktuellen PSA-Verlaufs.

Aktueller PSA-Wert: 1,43 ng/ml. Eine antihormonelle Therapie erfolgt nicht.

Wir haben folgende Befunde erhoben:

Parameter: Wert (abzüglich Baseline bei CTC) Falls G3+Nebenwirkung:

Denken wir Schritt für Schritt:

- Im gesamten Text wird der Begriff "Karnofsky-Index" nicht gefunden.

- Da kein Wert für den Karnofsky-Index vorhanden ist, wird FU12M_KPSSValue gemäß den Regeln auf "Unknown" gesetzt.

- Da kein Wert gefunden wurde, erübrigt sich die Suche nach dem Datum.

- FU12M_KPSSDate wird daher auf das Standarddatum "01-01-1901" gesetzt.

- Da weder ein Wert noch ein Datum bestimmt werden konnte, wird FU12M_IsKPSSDetermined auf 0 gesetzt.

Antwort:

{

"FU12M_IsKPSSDetermined": "0",

"FU12M_KPSSDate": null,

"FU12M_KPSSValue": null

}

### Antwort Format:

{

"FU12M_IsKPSSDetermined": "<1 oder 0>",

"FU12M_KPSSDate": "<Datum im Format DD-MM-YYYY , '01-01-1901' oder null>",

"FU12M_KPSSValue": "<KPSS-Wert, 'Unknown' oder null>"

}

"""

},

{

"name": "FU12M_PSAList",

"prompt": """

### Query:

Bitte extrahiere den gesamten Abschnitt, der mit "Tumormarker" oder "PSA-Verlauf" beginnt, aus dem bereitgestellten Text und gib ausschließlich ein einziges JSON-Objekt zurück. Zusätzliche Texte, Erklärungen oder mehrere JSON-Objekte sind nicht erlaubt.

### Schritte und Regeln:

1. Suche im Text nach einem Abschnitt, der mit "Tumormarker" oder "PSA-Verlauf" beginnt.

2. Extrahiere alle Informationen, die direkt nach "Tumormarker" oder "PSA-Verlauf" folgen, einschließlich Werte, Daten oder Notizen.

3. Wenn kein Abschnitt mit "Tumormarker" oder "PSA-Verlauf" gefunden wird, gib "Unknown" zurück.

### Beispiele:

1. Text: "Tumormarker PSA [ng/ml]: 6,86 (06/22); 5,89 (09/22)"

Denken wir Schritt für Schritt:

- Der Abschnitt beginnt mit "Tumormarker PSA [ng/ml]".

- Extrahiere alle nachfolgenden Inhalte: "6,86 (06/22); 5,89 (09/22)".

Antwort:

{

"FU12M_PSAList": "Tumormarker PSA [ng/ml]: 6,86 (06/22); 5,89 (09/22)"

}

2. Text: "Tumormarker PSA (ng/ml): 06/16: 4,2, 11/16: 4,6; 06/17: 4,4; 11/19: 7,15; 12/19: 7,3 Aktuell: 05/20: 6,7"

Denken wir Schritt für Schritt:

- Der Abschnitt beginnt mit "Tumormarker PSA (ng/ml)".

- Extrahiere alle nachfolgenden Inhalte: "06/16: 4,2, 11/16: 4,6; 06/17: 4,4; 11/19: 7,15; 12/19: 7,3 Aktuell: 05/20: 6,7".

Antwort:

{

"FU12M_PSAList": "Tumormarker PSA (ng/ml): 06/16: 4,2, 11/16: 4,6; 06/17: 4,4; 11/19: 7,15; 12/19: 7,3 Aktuell: 05/20: 6,7"

}

3. Text: "Keine Erwähnung von Tumormarkern im Bericht."

Denken wir Schritt für Schritt:

- Es gibt keinen Abschnitt, der mit "Tumormarker" beginnt.

- Gib "Unknown" zurück.

Antwort:

{

"FU12M_PSAList": "Unknown"

}

4. Text: "PSA-Verlauf (ng/ml): 5,29 (03/23); 5,02 (05/23); 5,85 (10/23); 7,47 (01/24); 7,99 (04/24); 9,9 (06/24)"

Denken wir Schritt für Schritt:

- Der Abschnitt beginnt mit "PSA-Verlauf".

- Extrahiere alle nachfolgenden Inhalte: "5,29 (03/23); 5,02 (05/23); 5,85 (10/23); 7,47 (01/24); 7,99 (04/24); 9,9 (06/24)".

Antwort:

{

"FU12M_PSAList": "PSA-Verlauf (ng/ml): 5,29 (03/23); 5,02 (05/23); 5,85 (10/23); 7,47 (01/24); 7,99 (04/24); 9,9 (06/24)"

}

5. Text: "Tumormarker iPSA: 14,1 ng/ml"

Denken wir Schritt für Schritt:

- Der Abschnitt beginnt mit "Tumormarker".

- Extrahiere alle nachfolgenden Inhalte: "14,1 ng/ml".

Antwort:

{

"FU12M_PSAList": "Tumormarker iPSA: 14,1 ng/ml"

}

### Antwort Format:

{

"FU12M_PSAList": "<Extrahierter Abschnitt oder 'Unknown'>"

}

"""

},

{

"name": "FU12M_PSA",

"prompt": """

### Query:

Extrahiere den PSA-Wert (Prostata-spezifisches Antigen) und das zugehörige Datum aus dem bereitgestellten Text. Die Werte befinden sich entweder in der PSA-Liste am Anfang des Dokuments oder innerhalb der "Sehr geehrter"-Einleitung und am Ende des Textes. Gib ausschließlich ein einziges JSON-Objekt zurück. Zusätzliche Texte, Erklärungen oder mehrere Objekte sind nicht erlaubt.

Der extrahierte Wert FU12M_PSALevel bezieht sich auf den PSA-Wert der Nachsorgeuntersuchung und spiegelt den aktuellen PSA-Status des Patienten wider.

### Schritte und Regeln:

1. Suche in der "Sehr geehrter"-Einleitung oder am Ende des Textes nach einem PSA-Wert.

- Relevante Begriffe zur Identifikation des richtigen Wertes sind: "PSA-Wertes", "Aktueller PSA", "Aktueller PSA-Wert", "PSA", "PSA Wert".

- Der PSA-Wert ist die Zahl, die in unmittelbarer Nähe dieser Begriffe steht.

2. Bestimme den PSA-Wert:

- Extrahiere die Zahl, die direkt neben den oben genannten Begriffen steht.

- Falls mehrere PSA-Werte im selben Abschnitt vorkommen, wähle den aktuellen Nachsorge-PSA-Wert anhand der Position und Formulierung.

- Falls kein PSA-Wert in der "Sehr geehrter"-Einleitung oder am Textende gefunden wird, überprüfe die PSA-Liste am Dokumentanfang.

- Wähle den letzten PSA-Wert aus der Liste am Dokumentanfang.

3. Bestimme das zugehörige Datum:

- Suche nach einem Datum direkt vor oder nach dem gefundenen PSA-Wert.

- Falls kein solches Datum vorhanden ist, überprüfe die PSA-Liste am Dokumentanfang:

- Die PSA-Liste enthält PSA-Werte aus verschiedenen Zeitpunkten, getrennt durch Kommas oder Semikolons.

- Jede Einheit in der PSA-Liste besteht aus einem PSA-Wert und möglicherweise einem zugehörigen Testdatum.

- Falls der letzte PSA-Wert aus der Liste mit dem extrahierten PSA-Wert übereinstimmt, übernehme dessen Datum.

- Falls kein übereinstimmender PSA-Wert in der Liste gefunden wird, überprüfe, ob nach "der sich am" ein Datum steht. Falls ja, verwende dieses Datum.

- Falls kein Datum nach "der sich am" gefunden wird, setze das Datum auf "01-01-1901".

4. Falls in der "Sehr geehrter"-Einleitung oder am Ende des Textes kein PSA-Wert gefunden wird:

- Überprüfe den letzten PSA-Wert in der PSA-Liste am Dokumentanfang.

- Wähle den letzten PSA-Wert aus der Liste.

- Falls ein Datum vorhanden ist, verwende dieses Datum.

- Falls kein Datum verfügbar ist, überprüfe, ob nach "der sich am" ein Datum steht. Falls ja, verwende dieses.

- Falls kein Datum nach "der sich am" gefunden wird, gib "01-01-1901" zurück.

5. Bestimme FU12M_IsPSADetermined:

- Wenn ein PSA-Wert gefunden wird, setze "FU12M_IsPSADetermined": "1".

- Wenn kein PSA-Wert gefunden wird, setze "FU12M_IsPSADetermined": "0", "FU12M_PSADate": "01-01-1901", "FU12M_PSALevel": "Unknown".

### Formatierungsregeln für das Datum:

- Falls ein Datum mit Tag, Monat und Jahr vorhanden ist, gib es im Format "DD-MM-YYYY" zurück.

- Falls nur Monat und Jahr angegeben sind (z. B. "MM/YY"), interpretiere es als den ersten Tag des Monats und gib das Datum im Format "01-MM-YYYY" zurück.

- Falls nur das Jahr vorhanden ist, interpretiere es als "01-01-YYYY".

- Falls kein Datum gefunden wird, gib "01-01-1901" zurück.

### Beispiele:

1. Text:

Tumormarker PSA [ng/ml];: 5,8 (07/21)

Sehr geehrter Herr Kollege,

ich berichte über unseren Patienten, der sich 3 Monate nach Abschluss der ultrahypofraktionierten Strahlentherapie in unserer Sprechstunde vorstellte.

Der zuletzt im November 2021 bestimmte PSA-Wert betrug 4,18. Patient ist in altersentsprechend gutem Allgemeinzustand. Es besteht seit der Bestrahlung weiterhin eine Blasenentleerungsstörung mit einem IPSS-Score von 12. Patient nimmt Tamsulosin. Sonst keine Hinweise für radiogene Toxizität.

Digital-rektale Untersuchung: perianal reizlos. Prostatavergrößert, nicht konsistenzvermehrt. Kein Tastbefund. Kein Tumor tastbar. Kein Blut.

Denken wir Schritt für Schritt:

- Der PSA-Wert "4,18" befindet sich im Satz "Der zuletzt im November 2021 bestimmte PSA-Wert betrug 4,18".

- In unmittelbarer Nähe des PSA-Werts wurde mit dem Hinweis "November 2021" ein Datum gefunden, das gemäß den Regeln verwendet wird, ohne die PSA-Liste zu überprüfen.

- Es wird überprüft, ob ein vollständiges Datum (Tag, Monat, Jahr) vorhanden ist. Da nur Monat und Jahr angegeben sind, wird gemäß den Regeln der erste Tag des Monats angenommen.

- Das Datum "November 2021" wird als "01-11-2021" formatiert.

- Da das Datum bereits eindeutig identifiziert wurde, wird die Liste nicht weiter überprüft.

Antwort:

{

"FU12M_IsPSADetermined": "1",

"FU12M_PSADate": "01-11-2021",

"FU12M_PSALevel": "4.18"

}

2. Text:

Tumormarker PSA [ng/ml]: 4,738 (11/20); 4,498 (02/21); 6,921 (08/21); 1,43 (01/2022)

Sehr geehrter Herr Kollege,

wir berichten über Herrn [ANONYMIZED], der sich am 04.03.2022 in unserer Ambulanz 3 nach Abschluss der o. g. Strahlentherapie zur strahlentherapeutischen Nachschau wieder vorstellte.

Wir danken für die Mitgabe des aktuellen PSA-Verlaufs.

Aktueller PSA-Wert: 1,43 ng/ml. Eine antihormonelle Therapie erfolgt nicht.

Denken wir Schritt für Schritt:

- Der PSA-Wert "1,43 ng/ml" befindet sich in der "Sehr geehrter"-Einleitung.

- Es gibt in unmittelbarer Nähe des PSA-Wertes kein explizites Datum.

- Überprüfung der PSA-Liste: Der letzte Wert in der Liste ist "1,43 (01/2022)".

- Der Wert "1,43" aus der Liste stimmt mit "1,43 ng/ml" überein.

- Es wird überprüft, ob ein vollständiges Datum (Tag, Monat, Jahr) vorhanden ist. Da nur Monat und Jahr angegeben sind, wird gemäß den Regeln der erste Tag des Monats angenommen.

- Das Datum "01/2022" wird als "01-01-2022" formatiert.

Antwort:

{

"FU12M_IsPSADetermined": "1",

"FU12M_PSADate": "01-01-2022",

"FU12M_PSALevel": "1.43"

}

3. Text:

Tumormarker PSA [ng/ml]: 10,5 (02/20); 15,23 (02/23, UKT); 7,75 (04/23)

Sehr geehrter Herr Kollege,

wir berichten über Herrn [ANONYMIZED], der sich am 16.06.2023 in unserer Ambulanz 3 nach Abschluss der o. g. Strahlentherapie zur strahlentherapeutischen Nachschau wieder vorstellte.

Herr [ANONYMIZED] berichtete Wohlbefinden und gute Belastbarkeit. Stuhlgang und Wasserlassen bereiten keine Probleme. Die letzte Nachsorge erfolgte am 04.2023 Urologe und war unauffällig.

Aktueller PSA: 7,75 ng/ml

Denken wir Schritt für Schritt:

- Der PSA-Wert "7,75 ng/ml" befindet sich in der "Sehr geehrter"-Einleitung.

- Es gibt in unmittelbarer Nähe des PSA-Wertes kein explizites Datum.

- Überprüfung der PSA-Liste: Der letzte Wert in der Liste ist "7,75 (04/23)".

- Der Wert "7,75" aus der Liste stimmt mit "7,75 ng/ml" überein.

- Es wird überprüft, ob ein vollständiges Datum (Tag, Monat, Jahr) vorhanden ist. Da nur Monat und Jahr angegeben sind, wird gemäß den Regeln der erste Tag des Monats angenommen.

- Das Datum "04/2023" wird als "01-04-2023" formatiert.

Antwort:

{

"FU12M_IsPSADetermined": "1",

"FU12M_PSADate": "01-04-2023",

"FU12M_PSALevel": "7.75"

}

4. Text:

Tumormarker iPSA: 5,27µg/l, 21.04.2020 7,28µg/l PSA: 07/2020 0,13; 10/2020 0,17; 10.02.2021 0,22µg/l 23.04.2021: 0,27

Sehr geehrter Herr Kollege

ich berichte über unseren gemeinsamen Patienten, Herr [ANONYMIZED]3 Monate nach Abschluss der Strahlentherapie in unserer Sprechstunde vorstellte.

Patient in sehr gutem Allgemeinzustand. Schmerzhafte Brustwarzen ohne Gynäkomastie unter Bicalutamid. Aktuelle PSA-Wert beträgt 0,05. Keine Nebenwirkungen hinsichtlich gastrointestinaler oder uro-genitaler Toxizität nach Strahlentherapie. Erektionsfähigkeit bereits vor Strahlentherapie erloschen.

Digital-rektale Untersuchung: Prostataloge leer, Schleimhaut glatt. Kein Blut. Perianale Haut reizlos.

Denken wir Schritt für Schritt:

- Der PSA-Wert "0,05" befindet sich in der "Sehr geehrter"-Einleitung.

- In unmittelbarer Nähe des PSA-Werts gibt es keine explizite Datumsangabe.

- Überprüfung der PSA-Liste: Der letzte Eintrag in der PSA-Liste ist "23.04.2021: 0,27", aber der Wert stimmt nicht mit dem aktuellen PSA-Wert "0,05" überein.

- Da kein passendes Datum in der Nähe oder in der Liste gefunden wird, wird das Standarddatum gemäß den Regeln zurückgegeben.

- Das Standarddatum "01-01-1901" wird gemäß den Formatierungsregeln verwendet.

Antwort:

{

"FU12M_IsPSADetermined": "1",

"FU12M_PSADate": "01-01-1901",

"FU12M_PSALevel": "0.05"

}

5. Text:

Tumormarker PSA [ng/ml]: 12,13 (06/17); 16,09 (07/17); 0,01 (02/18); 0,01 (05/18); 0,02 (09/18); 0,03 (05/19); 0,04 (08/19); 0,07 (02/20); 0,11 (08/20); 0,22 (02/21); 0,30 (06/21); 0,36 (09/21); 0,11 (03/22); 0,18 (06/22); 0,29 (09/22); 0,8 (01/23), <0,02 (07/23)

Sehr geehrter Herr Kollege,

wir berichten über Herrn [ANONYMIZED], der sich am 19.01.2024 in unserer Ambulanz nach Abschluss der o. g. Strahlentherapie zur strahlentherapeutischen Nachschau wieder vorstellte.

Herr [ANONYMIZED] berichtete Wohlbefinden und gute Belastbarkeit. Stuhlgang und Wasserlassen bereiten keine Probleme. Die letzte Nachsorge erfolgte am 12/2023 Ihnen und war unauffällig. Aktuell keine Gynäkomastie Zeichen. Bei bildmorphologischem Nachweis ossärer und lymphonodaler Metastasen eines vorbekannten Prostata-CA im Februar 2023 wurde im März 2023 eine Langzeit-ADT mit Trenantone und Apalutamid eingeleitet.

Aktueller PSA: <0,02 ng/ml

Denken wir Schritt für Schritt:

- Der PSA-Wert "<0,02 ng/ml" befindet sich in der "Sehr geehrter"-Einleitung.

- Es gibt in unmittelbarer Nähe des PSA-Wertes kein explizites Datum.

- Überprüfung der PSA-Liste nach "Tumormarker": Der letzte Wert in der Liste ist "<0,02 (07/23)".

- Der Wert "<0,02" aus der Liste stimmt mit "<0,02 ng/ml" überein.

- Es wird überprüft, ob ein vollständiges Datum (Tag, Monat, Jahr) vorhanden ist. Da nur Monat und Jahr angegeben sind, wird gemäß den Regeln der erste Tag des Monats angenommen.

- Das Datum "07/23" wird als "01-07-2023" formatiert.

- Der extrahierte PSA-Wert enthält das Zeichen "<", das entfernt wird, da der Wert numerisch gespeichert werden muss.

- Da ein passendes Datum aus der PSA-Liste gefunden wurde, ist keine weitere Überprüfung erforderlich.

Antwort:

{

"FU12M_IsPSADetermined": "1",

"FU12M_PSADate": "01-07-2023",

"FU12M_PSALevel": "0.02"

}

### Antwort Format:

{

"FU12M_IsPSADetermined": "<1 oder 0>",

"FU12M_PSADate": "<Datum im Format 'DD-MM-YYYY', '01-MM-YYYY' oder '01-01-1901'>",

"FU12M_PSALevel": "<PSA-Wert oder 'Unknown'>"

}

"""

},

{

"name": "FU12M_ECOG",

"prompt": """

### Query

Bitte extrahiere den ECOG-Status des Patienten aus dem Text und gib ausschließlich ein einziges JSON-Objekt auf Deutsch zurück. Zusätzliche Texte, Erklärungen oder mehrere JSON-Objekte sind nicht erlaubt.

**Vorprüfung – Wichtig:**

Bevor du beginnst: Prüfe, ob im Text **eine der folgenden Formulierungen** eindeutig vorkommt:

- „ECOG“

- „ECOG-Status“

- „ECOG I–IV“

**Wenn keine dieser Formulierungen vorkommt**, beende die Aufgabe sofort und gib exakt dieses JSON zurück:

```json

{

"FU12M_ECOG_YN": "0",

"FU12M_ECOG_DATE": null,

"FU12M_ECOG": null

}

```

**Wenn eine dieser Formulierungen vorhanden ist**, fahre mit den folgenden Extraktionsregeln fort.

*Wichtig:** Alle Datumsangaben müssen im Format `DD-MM-YYYY` angegeben werden – z. B. `13-09-2023`.

**Andere Formate wie `13.09.2023`, `2023-09-13` oder ausgeschriebene Daten sind nicht erlaubt.**

### Schritte und Regeln:

1. **ECOG nur bei expliziter Nennung**

Der ECOG-Status darf **nur dann extrahiert werden**, wenn im Text **eindeutig** einer der folgenden Begriffe enthalten ist (Groß-/Kleinschreibung egal):

- „ECOG“

- „ECOG-Status“

- „ECOG I-IV“

**Wenn keiner dieser Begriffe vorkommt**, darf **kein ECOG-Wert extrahiert werden**, auch nicht basierend auf einer Beschreibung des Allgemeinzustands.

2. **Ungültige Begriffe (nie als ECOG interpretieren):**

- „CTCAE“, „CTC“, „RTOG“

- „WHO-Performance“, „Karnofsky“

- „Allgemeinzustand“, „Grad“, „pflegebedürftig“, „bettlägerig“, „60%“, „100“, „ECOG-like“

3. **Gültige ECOG-Werte (nur wenn direkt bei ECOG-Nennung genannt):**

- Nur die Werte „0“, „1“, „2“, „3“ oder „4“ (auch römisch I–IV)

- Keine Prozentsätze, Grade, freien Beschreibungen etc.

4. **Datumsregel (nur wenn ECOG erkannt wurde):**

- Wenn ein Datum **direkt beim ECOG** genannt ist → verwende dieses.

- Falls nicht vorhanden: Verwende das **Datum der Patientenvorstellung** – also ein **explizites, einzelnes Vorstellungsdatum** wie z. B.:

> „Die Patientin stellte sich am 13.09.2023 in unserer Ambulanz vor.“

- **Achtung:** Datumsangaben aus einem **Behandlungszeitraum** dürfen **nicht** verwendet werden. Beispiel (ungültig):

> „…der sich vom 04.08. bis zum 15.08.2023 in unserer ambulanten radioonkologischen Behandlung befand“

- Falls kein Vorstellungdatum vorhanden ist: Verwende das **Dokumentdatum**.

- Wenn **kein zulässiges Datum gefunden werden kann**, setze das Datum auf `"01-01-1901"`.

- Wenn **kein ECOG erkannt wurde**, setze das Datum auf `null`.

5. **Datumsformat:**

- Immer exakt **DD-MM-YYYY** (z. B. „04-09-2021“)

- Keine Punkte, keine ISO-Formate, kein Textdatum

- Unsichere Daten → `"01-01-1901"` (falls ECOG vorhanden) oder `null` (falls nicht)

6. **Antwortformatierung:**

- `"FU12M_ECOG_YN"` = `"1"`, wenn ein gültiger ECOG-Status extrahiert wurde

- `"FU12M_ECOG_YN"` = `"0"`, **wenn kein ECOG im Text erkannt wurde**

- `"FU12M_ECOG_DATE"` = Datum im Format `"DD-MM-YYYY"` oder `"01-01-1901"` oder `null`

- `"FU12M_ECOG"` = ECOG-Wert zwischen `"0"` und `"4"` als String oder `null`

---

### Denke Schritt für Schritt:

1. **Kommt im Text eine der zulässigen ECOG-Formulierungen vor?**

→ Wenn **Nein**, antworte sofort mit:

```json

{

"BASE_ECOG_YN": "0",

"BASE_ECOG_DATE": null,

"BASE_ECOG": null

}

```

→ Wenn **Ja**, fahre fort mit der Extraktion von Wert und Datum.

2. **Ist der ECOG-Wert gültig?** (Nur 0–4, ggf. römisch)

→ Wenn **ungültig**, ebenfalls wie oben antworten.

→ Wenn **gültig**, extrahiere Datum gemäß Regel 4.

3. **Welches Datum soll verwendet werden?**

- Zuerst: Gibt es ein **Datum direkt bei der ECOG-Angabe**? → Verwende dieses.

- Falls nicht: Suche nach einem **klaren Vorstellungstermin**, z. B.:

> „Die Patientin stellte sich am 14.03.2023 vor.“

- **Achtung:** Wenn im Text ein **Behandlungszeitraum** genannt wird, z. B.:

> „…der sich vom 04.08. bis zum 15.08.2023 in Behandlung befand“

→ **Dieser Zeitraum darf nicht als Datum verwendet werden.**

- Falls kein Vorstellungstermin vorhanden ist: Verwende das **Dokumentdatum**.

- Wenn kein gültiges Datum erkennbar ist → `"01-01-1901"` verwenden.

4. **Gib am Ende ausschließlich das JSON-Objekt im korrekten Format zurück.**

---

### Beispiele:

1. Text: "Der ECOG-Status beträgt 0"

Denken wir Schritt für Schritt:

- Der Text enthält die Formulierung „ECOG-Status beträgt 0“, was gültig ist.

- Kein Datum vorhanden → Standardwert verwenden

Antwort:

{

"FU12M_ECOG_YN": "1",

"FU12M_ECOG_DATE": "01-01-1901",

"FU12M_ECOG": "0"

}

---

2. Text: "ECOG III laut Verlaufsnotiz vom 12.01.2022."

Denken wir Schritt für Schritt:

- „ECOG III“ ist eine gültige Formulierung → Wert = 3

- Datum ist vorhanden → 12-01-2022

Antwort:

{

"FU12M_ECOG_YN": "1",

"FU12M_ECOG_DATE": "12-01-2022",

"FU12M_ECOG": "3"

}

---

3. Text: "Die Patientin ist bettlägerig, benötigt durchgehend Hilfe."

Denken wir Schritt für Schritt:

- Es wird kein „ECOG“, „ECOG-Status“ etc. erwähnt. Nur eine Beschreibung.

- Kein ECOG-Wert darf extrahiert werden.

Antwort:

{

"FU12M_ECOG_YN": "0",

"FU12M_ECOG_DATE": null,

"FU12M_ECOG": null

}

---

4. Text: "Der Patient war vollständig abhängig von fremder Hilfe (ECOG IV)."

Denken wir Schritt für Schritt:

- Die Formulierung „ECOG IV“ wird erkannt → Wert = 4

- Kein explizites Datum → Standardwert verwenden

Antwort:

{

"FU12M_ECOG_YN": "1",

"FU12M_ECOG_DATE": "01-01-1901",

"FU12M_ECOG": "4"

}

---

5. Text: "Karnofsky 60%, Allgemeinzustand eingeschränkt."

Denken wir Schritt für Schritt:

- Keine zulässige Formulierung wie „ECOG“ enthalten.

- Kein ECOG-Wert extrahierbar.

Antwort:

{

"FU12M_ECOG_YN": "0",

"FU12M_ECOG_DATE": null,

"FU12M_ECOG": null

}

---

### Antwortformat:

{

"FU12M_ECOG_YN": "<'1' oder '0'>",

"FU12M_ECOG_DATE": "<Datum im Format DD-MM-YYYY oder '01-01-1901' oder null>",

"FU12M_ECOG": "<Zahl zwischen '0' und '4' oder null>"

}

"""

}

]

## Supplementary Text S1 d) user_prompts_FU24M

user_prompts_FU24M = [

{

"name": "FU24M_KPSS",

"prompt": """

### Query:

Bitte extrahiere die KPSS-Informationen aus dem Text und gib ausschließlich ein einziges JSON-Objekt zurück. Zusätzliche Texte, Erklärungen oder mehrere JSON-Objekte sind nicht erlaubt.

Die KPSS-Informationen bestehen aus dem Karnofsky-Index (FU24M_KPSSValue), der den allgemeinen Gesundheitszustand eines Patienten in Prozent angibt, und dem zugehörigen Datum (FU24M_KPSSDate), das angibt, wann dieser Wert dokumentiert wurde. Falls keine KPSS-Informationen im Text vorhanden sind, wird dies entsprechend als "Unknown" gekennzeichnet.

### Schritte und Regeln:

1. **Suche nach dem Begriff "Karnofsky-Index" oder einer Variante wie "Karnofsky".**

- Falls **kein solcher Begriff im Text vorkommt**:

- Setze `"FU24M_IsKPSSDetermined"` auf `"0"`.

- Setze `"FU24M_KPSSValue"` auf `null`.

- Setze `"FU24M_KPSSDate"` auf `null`.

- **Beende die Extraktion an dieser Stelle.**

- **Es dürfen keine Werte geraten, geschätzt oder auf andere Weise abgeleitet werden.**

2. **Wenn "Karnofsky" gefunden wurde:**

- **Extrahiere den FU24M_KPSSValue:**

- Der Wert steht direkt hinter dem gefundenen Begriff.

- Falls ein Bereich wie "80 – 90 %" angegeben ist, wähle den **niedrigeren Wert** (hier: `"80"`).

- Falls **kein klarer Wert angegeben ist**, obwohl "Karnofsky" vorkommt:

- Setze `"FU24M_KPSSValue"` auf `"Unknown"`.

- Setze `"FU24M_IsKPSSDetermined"` auf `"1"`.

- Setze `"FU24M_KPSSDate"` auf `null`.

- **Beende die Extraktion an dieser Stelle.**

- **Extrahiere das Datum FU24M_KPSSDate:**

- Suche im Abschnitt, der mit „Sehr geehrter“ beginnt, nach dem Ausdruck **„der sich am <Datum>“**.

- Falls ein solches Datum vorhanden ist, gib es im Format `"DD-MM-YYYY"` zurück.

- Falls kein Datum gefunden wird, verwende `"01-01-1901"`.

3. **Zusammenfassend gilt:**

- Nur wenn `"FU24M_IsKPSSDetermined"` den Wert `"1"` hat, dürfen `"FU24M_KPSSValue"` und `"FU24M_KPSSDate"` **einen gültigen Wert enthalten**.

- Wenn `"FU24M_IsKPSSDetermined"` den Wert `"0"` hat, müssen **beide anderen Felder zwingend `null` sein** – selbst wenn im Text ein Datum genannt wird.

---

### Formatierungsregeln für das Datum:

- Wenn das Datum Tag, Monat und Jahr enthält, gib es im Format `"DD-MM-YYYY"` zurück.

- Falls kein Datum gefunden wird, gib `"01-01-1901"` zurück.

- Falls kein KPSS-Wert extrahiert wurde, muss das Datum `null` sein.

---

### Beispiele:

1. Text:

Sehr geehrter Herr Kollege,

wir berichten über Herrn [ANONYMIZED], der sich am 17.10.2024 in unserer Ambulanz 3 nach Abschluss der o. g. Strahlentherapie zur strahlentherapeutischen Nachschau wieder vorstellte.

Herr [ANONYMIZED] berichtete mäßiges Wohlbefinden und eingeschränkte Belastbarkeit, dies sei aber auf Grund seiner kardialen Erkrankungen schon länger bekannt und konstant. Stuhlgang und Wasserlassen bereiten keine Probleme. Bisher sei keine ambulante Vorstellung beim Urologen erfolgt.

Aktueller PSA: 0,91 µg/l, IPSS 3

Untersuchungsbefund:

Karnofsky-Index 80 -90 %, Körpergewicht 90 kg.

Denken wir Schritt für Schritt:

- Der Begriff "Karnofsky-Index" ist vorhanden.

- Die Werte sind "80 - 90 %". Der niedrigeren Wert ist "80".

- Das Datum steht hinter "der sich am" und lautet "17.10.2024".

- Das Datum wird ins Format "DD-MM-YYYY" konvertiert: "17-10-2024".

- Setze "FU24M_IsKPSSDetermined" auf 1.

Antwort:

{

"FU24M_IsKPSSDetermined": "1",

"FU24M_KPSSDate": "17-10-2024",

"FU24M_KPSSValue": "90"

}

2. Text:

Sehr geehrter Herr Kollege,

wir berichten über Herrn [ANONYMIZED], der sich am 16.06.2023 in unserer Ambulanz 3 nach Abschluss der o. g. Strahlentherapie zur strahlentherapeutischen Nachschau wieder vorstellte.

Herr [ANONYMIZED] berichtete Wohlbefinden und gute Belastbarkeit. Stuhlgang und Wasserlassen bereiten keine Probleme. Die letzte Nachsorge erfolgte am 04.2023 Urologe und war unauffällig.

Aktueller PSA: 7,75 ng/ml

Die antiandrogene Therapie mit einem GnRH-Analogon wird nicht durchgeführt.

Untersuchungsbefund:

Karnofsky-Index 100 %, Körpergewicht 74 kg.

Denken wir Schritt für Schritt:

- Der Begriff "Karnofsky-Index" wird im Abschnitt "Untersuchungsbefund" gefunden.

- Direkt hinter dem Begriff steht der Wert "100 %". Da es sich um einen eindeutigen Wert handelt und kein Zahlenbereich vorliegt, wird "100" als FU24M_KPSSValue übernommen.

- Anschließend wird im Abschnitt, nach dem Ausdruck "der sich am" gesucht, um das FU24M_KPSSDate zu bestimmen. Es wird der Text "der sich am 16.06.2023" identifiziert.

- Das Datum "16.06.2023" wird gemäß den Formatierungsregeln ins Format "DD-MM-YYYY" umgewandelt und ergibt "16-06-2023".

- Setze "FU24M_IsKPSSDetermined" auf 1.

Antwort:

{

"FU24M_IsKPSSDetermined": "1",

"FU24M_KPSSDate": "16-06-2023",

"FU24M_KPSSValue": "100"

}

3. Text:

Sehr geehrter Herr Kollege,

wir berichten über Herrn [ANONYMIZED] 3 Monate nach Abschluss der o.g. Strahlentherapie zur strahlentherapeutischen Nachschau wieder vorstellte.

Herr [ANONYMIZED] welche nach der OP bereits deutlich gebessert war und nach Bestrahlung nun wieder verschlechtert vorliegt. Vorlagen werden bei Terminen außerhalb genutzt. Der Beckenboden wird seit der Reha regelmäßig beübt. Die letzte uroonkologische Nachsorge war unauffällig. Ein nächster Termin ist für den 26.04.21 eingeplant.

Aktueller PSA: <0,0 (1/2021) unter ADT (seit 10/2020)

Untersuchungsbefund:

Karnofsky-Index 90 %, Körpergewicht 85,5 kg, konstant.

Denken wir Schritt für Schritt:

- Der Begriff "Karnofsky-Index" wird im Abschnitt "Untersuchungsbefund" gefunden.

- Direkt dahinter steht der Wert "90 %". Da es sich um einen eindeutigen Wert handelt, wird "90" als FU24M_KPSSValue extrahiert.

- Anschließend wird im Abschnitt, der mit "Sehr geehrter" beginnt, nach dem Ausdruck "der sich am" gesucht, um das Datum für FU24M_KPSSDate zu bestimmen.

- Da kein "der sich am"-Datum vorhanden ist und die Angabe "3 Monate nach Abschluss der o.g. Strahlentherapie" kein konkretes Datum liefert, wird gemäß den Regeln das Standarddatum "01-01-1901" verwendet.

- Da der Wert erfolgreich extrahiert wurde, wird FU24M_IsKPSSDetermined auf 1 gesetzt.

Antwort:

{

"FU24M_IsKPSSDetermined": "1",

"FU24M_KPSSDate": "01-01-1901",

"FU24M_KPSSValue": "90"

}

4. Text:

Wir berichten über Herrn [ANONYMIZED], der sich am 08.09.2023 in unserer Ambulanz 4 nach Abschluss der o. g. Strahlentherapie zur strahlentherapeutischen Nachschau wieder vorstellte.

Herr [ANONYMIZED] berichtete Wohlbefinden und gute Belastbarkeit. Stuhlgang und Wasserlassen bereiten keine Probleme. Die letzte Nachsorge erfolgte Ihnen und war unauffällig.

Aktueller PSA: <0,0006

Untersuchungsbefund:

Karnofsky-Index 100 %.

Denken wir Schritt für Schritt:

- Der Begriff "Karnofsky-Index" wird im Abschnitt "Untersuchungsbefund" gefunden.

- Der Wert direkt dahinter lautet "100 %". Da es sich um eine einzelne Zahl handelt, wird dieser Wert direkt als FU24M_KPSSValue übernommen.

- Anschließend wird im Abschnitt, der mit "Sehr geehrter" beginnt, nach dem Ausdruck "der sich am" gesucht, um das Datum für FU24M_KPSSDate zu bestimmen. In der Einleitung findet sich die Datumsangabe "08.09.2023".

- Es enthält Tag, Monat und Jahr und wird im Format "DD-MM-YYYY" übernommen: "08-09-2023".

- Setze "FU24M_IsKPSSDetermined" auf 1.

Antwort:

{

"FU24M_IsKPSSDetermined": "1",

"FU24M_KPSSDate": "08-09-2023",

"FU24M_KPSSValue": "100"

}

5. Text:

Sehr geehrter Herr Kollege,

wir berichten über Herrn [ANONYMIZED], der sich am 04.03.2022 in unserer Ambulanz 3 nach Abschluss der o. g. Strahlentherapie zur strahlentherapeutischen Nachschau wieder vorstellte.

Wir danken für die Mitgabe des aktuellen PSA-Verlaufs.

Aktueller PSA-Wert: 1,43 ng/ml. Eine antihormonelle Therapie erfolgt nicht.

Wir haben folgende Befunde erhoben:

Parameter: Wert (abzüglich Baseline bei CTC) Falls G3+Nebenwirkung:

Denken wir Schritt für Schritt:

- Im gesamten Text wird der Begriff "Karnofsky-Index" nicht gefunden.

- Da kein Wert für den Karnofsky-Index vorhanden ist, wird FU24M_KPSSValue gemäß den Regeln auf "Unknown" gesetzt.

- Da kein Wert gefunden wurde, erübrigt sich die Suche nach dem Datum.

- FU24M_KPSSDate wird daher auf das Standarddatum "01-01-1901" gesetzt.

- Da weder ein Wert noch ein Datum bestimmt werden konnte, wird FU24M_IsKPSSDetermined auf 0 gesetzt.

Antwort:

{

"FU24M_IsKPSSDetermined": "0",

"FU24M_KPSSDate": null,

"FU24M_KPSSValue": null

}

### Antwort Format:

{

"FU24M_IsKPSSDetermined": "<1 oder 0>",

"FU24M_KPSSDate": "<Datum im Format DD-MM-YYYY , '01-01-1901' oder null>",

"FU24M_KPSSValue": "<KPSS-Wert, 'Unknown' oder null>"

}

"""

},

{

"name": "FU24M_PSAList",

"prompt": """

### Query:

Bitte extrahiere den gesamten Abschnitt, der mit "Tumormarker" oder "PSA-Verlauf" beginnt, aus dem bereitgestellten Text und gib ausschließlich ein einziges JSON-Objekt zurück. Zusätzliche Texte, Erklärungen oder mehrere JSON-Objekte sind nicht erlaubt.

### Schritte und Regeln:

1. Suche im Text nach einem Abschnitt, der mit "Tumormarker" oder "PSA-Verlauf" beginnt.

2. Extrahiere alle Informationen, die direkt nach "Tumormarker" oder "PSA-Verlauf" folgen, einschließlich Werte, Daten oder Notizen.

3. Wenn kein Abschnitt mit "Tumormarker" oder "PSA-Verlauf" gefunden wird, gib "Unknown" zurück.

### Beispiele:

1. Text: "Tumormarker PSA [ng/ml]: 6,86 (06/22); 5,89 (09/22)"

Denken wir Schritt für Schritt:

- Der Abschnitt beginnt mit "Tumormarker PSA [ng/ml]".

- Extrahiere alle nachfolgenden Inhalte: "6,86 (06/22); 5,89 (09/22)".

Antwort:

{

"FU24M_PSAList": "Tumormarker PSA [ng/ml]: 6,86 (06/22); 5,89 (09/22)"

}

2. Text: "Tumormarker PSA (ng/ml): 06/16: 4,2, 11/16: 4,6; 06/17: 4,4; 11/19: 7,15; 12/19: 7,3 Aktuell: 05/20: 6,7"

Denken wir Schritt für Schritt:

- Der Abschnitt beginnt mit "Tumormarker PSA (ng/ml)".

- Extrahiere alle nachfolgenden Inhalte: "06/16: 4,2, 11/16: 4,6; 06/17: 4,4; 11/19: 7,15; 12/19: 7,3 Aktuell: 05/20: 6,7".

Antwort:

{

"FU24M_PSAList": "Tumormarker PSA (ng/ml): 06/16: 4,2, 11/16: 4,6; 06/17: 4,4; 11/19: 7,15; 12/19: 7,3 Aktuell: 05/20: 6,7"

}

3. Text: "Keine Erwähnung von Tumormarkern im Bericht."

Denken wir Schritt für Schritt:

- Es gibt keinen Abschnitt, der mit "Tumormarker" beginnt.

- Gib "Unknown" zurück.

Antwort:

{

"FU24M_PSAList": "Unknown"

}

4. Text: "PSA-Verlauf (ng/ml): 5,29 (03/23); 5,02 (05/23); 5,85 (10/23); 7,47 (01/24); 7,99 (04/24); 9,9 (06/24)"

Denken wir Schritt für Schritt:

- Der Abschnitt beginnt mit "PSA-Verlauf".

- Extrahiere alle nachfolgenden Inhalte: "5,29 (03/23); 5,02 (05/23); 5,85 (10/23); 7,47 (01/24); 7,99 (04/24); 9,9 (06/24)".

Antwort:

{

"FU24M_PSAList": "PSA-Verlauf (ng/ml): 5,29 (03/23); 5,02 (05/23); 5,85 (10/23); 7,47 (01/24); 7,99 (04/24); 9,9 (06/24)"

}

5. Text: "Tumormarker iPSA: 14,1 ng/ml"

Denken wir Schritt für Schritt:

- Der Abschnitt beginnt mit "Tumormarker".

- Extrahiere alle nachfolgenden Inhalte: "14,1 ng/ml".

Antwort:

{

"FU24M_PSAList": "Tumormarker iPSA: 14,1 ng/ml"

}

### Antwort Format:

{

"FU24M_PSAList": "<Extrahierter Abschnitt oder 'Unknown'>"

}

"""

},

{

"name": "FU24M_PSA",

"prompt": """

### Query:

Extrahiere den PSA-Wert (Prostata-spezifisches Antigen) und das zugehörige Datum aus dem bereitgestellten Text. Die Werte befinden sich entweder in der PSA-Liste am Anfang des Dokuments oder innerhalb der "Sehr geehrter"-Einleitung und am Ende des Textes. Gib ausschließlich ein einziges JSON-Objekt zurück. Zusätzliche Texte, Erklärungen oder mehrere Objekte sind nicht erlaubt.

Der extrahierte Wert FU24M_PSALevel bezieht sich auf den PSA-Wert der Nachsorgeuntersuchung und spiegelt den aktuellen PSA-Status des Patienten wider.

### Schritte und Regeln:

1. Suche in der "Sehr geehrter"-Einleitung oder am Ende des Textes nach einem PSA-Wert.

- Relevante Begriffe zur Identifikation des richtigen Wertes sind: "PSA-Wertes", "Aktueller PSA", "Aktueller PSA-Wert", "PSA", "PSA Wert".

- Der PSA-Wert ist die Zahl, die in unmittelbarer Nähe dieser Begriffe steht.

2. Bestimme den PSA-Wert:

- Extrahiere die Zahl, die direkt neben den oben genannten Begriffen steht.

- Falls mehrere PSA-Werte im selben Abschnitt vorkommen, wähle den aktuellen Nachsorge-PSA-Wert anhand der Position und Formulierung.

- Falls kein PSA-Wert in der "Sehr geehrter"-Einleitung oder am Textende gefunden wird, überprüfe die PSA-Liste am Dokumentanfang.

- Wähle den letzten PSA-Wert aus der Liste am Dokumentanfang.

3. Bestimme das zugehörige Datum:

- Suche nach einem Datum direkt vor oder nach dem gefundenen PSA-Wert.

- Falls kein solches Datum vorhanden ist, überprüfe die PSA-Liste am Dokumentanfang:

- Die PSA-Liste enthält PSA-Werte aus verschiedenen Zeitpunkten, getrennt durch Kommas oder Semikolons.

- Jede Einheit in der PSA-Liste besteht aus einem PSA-Wert und möglicherweise einem zugehörigen Testdatum.

- Falls der letzte PSA-Wert aus der Liste mit dem extrahierten PSA-Wert übereinstimmt, übernehme dessen Datum.

- Falls kein übereinstimmender PSA-Wert in der Liste gefunden wird, überprüfe, ob nach "der sich am" ein Datum steht. Falls ja, verwende dieses Datum.

- Falls kein Datum nach "der sich am" gefunden wird, setze das Datum auf "01-01-1901".

4. Falls in der "Sehr geehrter"-Einleitung oder am Ende des Textes kein PSA-Wert gefunden wird:

- Überprüfe den letzten PSA-Wert in der PSA-Liste am Dokumentanfang.

- Wähle den letzten PSA-Wert aus der Liste.

- Falls ein Datum vorhanden ist, verwende dieses Datum.

- Falls kein Datum verfügbar ist, überprüfe, ob nach "der sich am" ein Datum steht. Falls ja, verwende dieses.

- Falls kein Datum nach "der sich am" gefunden wird, gib "01-01-1901" zurück.

5. Bestimme FU24M_IsPSADetermined:

- Wenn ein PSA-Wert gefunden wird, setze "FU24M_IsPSADetermined": "1".

- Wenn kein PSA-Wert gefunden wird, setze "FU24M_IsPSADetermined": "0", "FU24M_PSADate": "01-01-1901", "FU24M_PSALevel": "Unknown".

### Formatierungsregeln für das Datum:

- Falls ein Datum mit Tag, Monat und Jahr vorhanden ist, gib es im Format "DD-MM-YYYY" zurück.

- Falls nur Monat und Jahr angegeben sind (z. B. "MM/YY"), interpretiere es als den ersten Tag des Monats und gib das Datum im Format "01-MM-YYYY" zurück.

- Falls nur das Jahr vorhanden ist, interpretiere es als "01-01-YYYY".

- Falls kein Datum gefunden wird, gib "01-01-1901" zurück.

### Beispiele:

1. Text:

Tumormarker PSA [ng/ml];: 5,8 (07/21)

Sehr geehrter Herr Kollege,

ich berichte über unseren Patienten, der sich 3 Monate nach Abschluss der ultrahypofraktionierten Strahlentherapie in unserer Sprechstunde vorstellte.

Der zuletzt im November 2021 bestimmte PSA-Wert betrug 4,18. Patient ist in altersentsprechend gutem Allgemeinzustand. Es besteht seit der Bestrahlung weiterhin eine Blasenentleerungsstörung mit einem IPSS-Score von 12. Patient nimmt Tamsulosin. Sonst keine Hinweise für radiogene Toxizität.

Digital-rektale Untersuchung: perianal reizlos. Prostatavergrößert, nicht konsistenzvermehrt. Kein Tastbefund. Kein Tumor tastbar. Kein Blut.

Denken wir Schritt für Schritt:

- Der PSA-Wert "4,18" befindet sich im Satz "Der zuletzt im November 2021 bestimmte PSA-Wert betrug 4,18".

- In unmittelbarer Nähe des PSA-Werts wurde mit dem Hinweis "November 2021" ein Datum gefunden, das gemäß den Regeln verwendet wird, ohne die PSA-Liste zu überprüfen.

- Es wird überprüft, ob ein vollständiges Datum (Tag, Monat, Jahr) vorhanden ist. Da nur Monat und Jahr angegeben sind, wird gemäß den Regeln der erste Tag des Monats angenommen.

- Das Datum "November 2021" wird als "01-11-2021" formatiert.

- Da das Datum bereits eindeutig identifiziert wurde, wird die Liste nicht weiter überprüft.

Antwort:

{

"FU24M_IsPSADetermined": "1",

"FU24M_PSADate": "01-11-2021",

"FU24M_PSALevel": "4.18"

}

2. Text:

Tumormarker PSA [ng/ml]: 4,738 (11/20); 4,498 (02/21); 6,921 (08/21); 1,43 (01/2022)

Sehr geehrter Herr Kollege,

wir berichten über Herrn [ANONYMIZED], der sich am 04.03.2022 in unserer Ambulanz 3 nach Abschluss der o. g. Strahlentherapie zur strahlentherapeutischen Nachschau wieder vorstellte.

Wir danken für die Mitgabe des aktuellen PSA-Verlaufs.

Aktueller PSA-Wert: 1,43 ng/ml. Eine antihormonelle Therapie erfolgt nicht.

Denken wir Schritt für Schritt:

- Der PSA-Wert "1,43 ng/ml" befindet sich in der "Sehr geehrter"-Einleitung.

- Es gibt in unmittelbarer Nähe des PSA-Wertes kein explizites Datum.

- Überprüfung der PSA-Liste: Der letzte Wert in der Liste ist "1,43 (01/2022)".

- Der Wert "1,43" aus der Liste stimmt mit "1,43 ng/ml" überein.

- Es wird überprüft, ob ein vollständiges Datum (Tag, Monat, Jahr) vorhanden ist. Da nur Monat und Jahr angegeben sind, wird gemäß den Regeln der erste Tag des Monats angenommen.

- Das Datum "01/2022" wird als "01-01-2022" formatiert.

Antwort:

{

"FU24M_IsPSADetermined": "1",

"FU24M_PSADate": "01-01-2022",

"FU24M_PSALevel": "1.43"

}

3. Text:

Tumormarker PSA [ng/ml]: 10,5 (02/20); 15,23 (02/23, UKT); 7,75 (04/23)

Sehr geehrter Herr Kollege,

wir berichten über Herrn [ANONYMIZED], der sich am 16.06.2023 in unserer Ambulanz 3 nach Abschluss der o. g. Strahlentherapie zur strahlentherapeutischen Nachschau wieder vorstellte.

Herr [ANONYMIZED] berichtete Wohlbefinden und gute Belastbarkeit. Stuhlgang und Wasserlassen bereiten keine Probleme. Die letzte Nachsorge erfolgte am 04.2023 Urologe und war unauffällig.

Aktueller PSA: 7,75 ng/ml

Denken wir Schritt für Schritt:

- Der PSA-Wert "7,75 ng/ml" befindet sich in der "Sehr geehrter"-Einleitung.

- Es gibt in unmittelbarer Nähe des PSA-Wertes kein explizites Datum.

- Überprüfung der PSA-Liste: Der letzte Wert in der Liste ist "7,75 (04/23)".

- Der Wert "7,75" aus der Liste stimmt mit "7,75 ng/ml" überein.

- Es wird überprüft, ob ein vollständiges Datum (Tag, Monat, Jahr) vorhanden ist. Da nur Monat und Jahr angegeben sind, wird gemäß den Regeln der erste Tag des Monats angenommen.

- Das Datum "04/2023" wird als "01-04-2023" formatiert.

Antwort:

{

"FU24M_IsPSADetermined": "1",

"FU24M_PSADate": "01-04-2023",

"FU24M_PSALevel": "7.75"

}

4. Text:

Tumormarker iPSA: 5,27µg/l, 21.04.2020 7,28µg/l PSA: 07/2020 0,13; 10/2020 0,17; 10.02.2021 0,22µg/l 23.04.2021: 0,27

Sehr geehrter Herr Kollege,

ich berichte über unseren gemeinsamen Patienten, Herr [ANONYMIZED]3 Monate nach Abschluss der Strahlentherapie in unserer Sprechstunde vorstellte.

Patient in sehr gutem Allgemeinzustand. Schmerzhafte Brustwarzen ohne Gynäkomastie unter Bicalutamid. Aktuelle PSA-Wert beträgt 0,05. Keine Nebenwirkungen hinsichtlich gastrointestinaler oder uro-genitaler Toxizität nach Strahlentherapie. Erektionsfähigkeit bereits vor Strahlentherapie erloschen.

Digital-rektale Untersuchung: Prostataloge leer, Schleimhaut glatt. Kein Blut. Perianale Haut reizlos.

Denken wir Schritt für Schritt:

- Der PSA-Wert "0,05" befindet sich in der "Sehr geehrter"-Einleitung.

- In unmittelbarer Nähe des PSA-Werts gibt es keine explizite Datumsangabe.

- Überprüfung der PSA-Liste: Der letzte Eintrag in der PSA-Liste ist "23.04.2021: 0,27", aber der Wert stimmt nicht mit dem aktuellen PSA-Wert "0,05" überein.

- Da kein passendes Datum in der Nähe oder in der Liste gefunden wird, wird das Standarddatum gemäß den Regeln zurückgegeben.

- Das Standarddatum "01-01-1901" wird gemäß den Formatierungsregeln verwendet.

Antwort:

{

"FU24M_IsPSADetermined": "1",

"FU24M_PSADate": "01-01-1901",

"FU24M_PSALevel": "0.05"

}

5. Text:

Tumormarker PSA [ng/ml]: 12,13 (06/17); 16,09 (07/17); 0,01 (02/18); 0,01 (05/18); 0,02 (09/18); 0,03 (05/19); 0,04 (08/19); 0,07 (02/20); 0,11 (08/20); 0,22 (02/21); 0,30 (06/21); 0,36 (09/21); 0,11 (03/22); 0,18 (06/22); 0,29 (09/22); 0,8 (01/23), <0,02 (07/23)

Sehr geehrter Herr Kollege,

wir berichten über Herrn [ANONYMIZED], der sich am 19.01.2024 in unserer Ambulanz nach Abschluss der o. g. Strahlentherapie zur strahlentherapeutischen Nachschau wieder vorstellte.

Herr [ANONYMIZED] berichtete Wohlbefinden und gute Belastbarkeit. Stuhlgang und Wasserlassen bereiten keine Probleme. Die letzte Nachsorge erfolgte am 12/2023 Ihnen und war unauffällig. Aktuell keine Gynäkomastie Zeichen. Bei bildmorphologischem Nachweis ossärer und lymphonodaler Metastasen eines vorbekannten Prostata-CA im Februar 2023 wurde im März 2023 eine Langzeit-ADT mit Trenantone und Apalutamid eingeleitet.

Aktueller PSA: <0,02 ng/ml

Denken wir Schritt für Schritt:

- Der PSA-Wert "<0,02 ng/ml" befindet sich in der "Sehr geehrter"-Einleitung.

- Es gibt in unmittelbarer Nähe des PSA-Wertes kein explizites Datum.

- Überprüfung der PSA-Liste nach "Tumormarker": Der letzte Wert in der Liste ist "<0,02 (07/23)".

- Der Wert "<0,02" aus der Liste stimmt mit "<0,02 ng/ml" überein.

- Es wird überprüft, ob ein vollständiges Datum (Tag, Monat, Jahr) vorhanden ist. Da nur Monat und Jahr angegeben sind, wird gemäß den Regeln der erste Tag des Monats angenommen.

- Das Datum "07/23" wird als "01-07-2023" formatiert.

- Der extrahierte PSA-Wert enthält das Zeichen "<", das entfernt wird, da der Wert numerisch gespeichert werden muss.

- Da ein passendes Datum aus der PSA-Liste gefunden wurde, ist keine weitere Überprüfung erforderlich.

Antwort:

{

"FU24M_IsPSADetermined": "1",

"FU24M_PSADate": "01-07-2023",

"FU24M_PSALevel": "0.02"

}

### Antwort Format:

{

"FU24M_IsPSADetermined": "<1 oder 0>",

"FU24M_PSADate": "<Datum im Format 'DD-MM-YYYY', '01-MM-YYYY' oder '01-01-1901'>",

"FU24M_PSALevel": "<PSA-Wert oder 'Unknown'>"

}

"""

},

{

"name": "FU24M_ECOG",

"prompt": """

### Query

Bitte extrahiere den ECOG-Status des Patienten aus dem Text und gib ausschließlich ein einziges JSON-Objekt auf Deutsch zurück. Zusätzliche Texte, Erklärungen oder mehrere JSON-Objekte sind nicht erlaubt.

**Vorprüfung – Wichtig:**

Bevor du beginnst: Prüfe, ob im Text **eine der folgenden Formulierungen** eindeutig vorkommt:

- „ECOG“

- „ECOG-Status“

- „ECOG I–IV“

**Wenn keine dieser Formulierungen vorkommt**, beende die Aufgabe sofort und gib exakt dieses JSON zurück:

```json

{

"FU24M_ECOG_YN": "0",

"FU24M_ECOG_DATE": null,

"FU24M_ECOG": null

}

```

**Wenn eine dieser Formulierungen vorhanden ist**, fahre mit den folgenden Extraktionsregeln fort.

*Wichtig:** Alle Datumsangaben müssen im Format `DD-MM-YYYY` angegeben werden – z. B. `13-09-2023`.

**Andere Formate wie `13.09.2023`, `2023-09-13` oder ausgeschriebene Daten sind nicht erlaubt.**

### Schritte und Regeln:

1. **ECOG nur bei expliziter Nennung**

Der ECOG-Status darf **nur dann extrahiert werden**, wenn im Text **eindeutig** einer der folgenden Begriffe enthalten ist (Groß-/Kleinschreibung egal):

- „ECOG“

- „ECOG-Status“

- „ECOG I-IV“

**Wenn keiner dieser Begriffe vorkommt**, darf **kein ECOG-Wert extrahiert werden**, auch nicht basierend auf einer Beschreibung des Allgemeinzustands.

2. **Ungültige Begriffe (nie als ECOG interpretieren):**

- „CTCAE“, „CTC“, „RTOG“

- „WHO-Performance“, „Karnofsky“

- „Allgemeinzustand“, „Grad“, „pflegebedürftig“, „bettlägerig“, „60%“, „100“, „ECOG-like“

3. **Gültige ECOG-Werte (nur wenn direkt bei ECOG-Nennung genannt):**

- Nur die Werte „0“, „1“, „2“, „3“ oder „4“ (auch römisch I–IV)

- Keine Prozentsätze, Grade, freien Beschreibungen etc.

4. **Datumsregel (nur wenn ECOG erkannt wurde):**

- Wenn ein Datum **direkt beim ECOG** genannt ist → verwende dieses.

- Falls nicht vorhanden: Verwende das **Datum der Patientenvorstellung** – also ein **explizites, einzelnes Vorstellungsdatum** wie z. B.:

> „Die Patientin stellte sich am 13.09.2023 in unserer Ambulanz vor.“

- **Achtung:** Datumsangaben aus einem **Behandlungszeitraum** dürfen **nicht** verwendet werden. Beispiel (ungültig):

> „…der sich vom 04.08. bis zum 15.08.2023 in unserer ambulanten radioonkologischen Behandlung befand“

- Falls kein Vorstellungdatum vorhanden ist: Verwende das **Dokumentdatum**.

- Wenn **kein zulässiges Datum gefunden werden kann**, setze das Datum auf `"01-01-1901"`.

- Wenn **kein ECOG erkannt wurde**, setze das Datum auf `null`.

5. **Datumsformat:**

- Immer exakt **DD-MM-YYYY** (z. B. „04-09-2021“)

- Keine Punkte, keine ISO-Formate, kein Textdatum

- Unsichere Daten → `"01-01-1901"` (falls ECOG vorhanden) oder `null` (falls nicht)

6. **Antwortformatierung:**

- `"FU24M_ECOG_YN"` = `"1"`, wenn ein gültiger ECOG-Status extrahiert wurde

- `"FU24M_ECOG_YN"` = `"0"`, **wenn kein ECOG im Text erkannt wurde**

- `"FU24M_ECOG_DATE"` = Datum im Format `"DD-MM-YYYY"` oder `"01-01-1901"` oder `null`

- `"FU24M_ECOG"` = ECOG-Wert zwischen `"0"` und `"4"` als String oder `null`

---

### Denke Schritt für Schritt:

1. **Kommt im Text eine der zulässigen ECOG-Formulierungen vor?**

→ Wenn **Nein**, antworte sofort mit:

```json

{

"BASE_ECOG_YN": "0",

"BASE_ECOG_DATE": null,

"BASE_ECOG": null

}

```

→ Wenn **Ja**, fahre fort mit der Extraktion von Wert und Datum.

2. **Ist der ECOG-Wert gültig?** (Nur 0–4, ggf. römisch)

→ Wenn **ungültig**, ebenfalls wie oben antworten.

→ Wenn **gültig**, extrahiere Datum gemäß Regel 4.

3. **Welches Datum soll verwendet werden?**

- Zuerst: Gibt es ein **Datum direkt bei der ECOG-Angabe**? → Verwende dieses.

- Falls nicht: Suche nach einem **klaren Vorstellungstermin**, z. B.:

> „Die Patientin stellte sich am 14.03.2023 vor.“

- **Achtung:** Wenn im Text ein **Behandlungszeitraum** genannt wird, z. B.:

> „…der sich vom 04.08. bis zum 15.08.2023 in Behandlung befand“

→ **Dieser Zeitraum darf nicht als Datum verwendet werden.**

- Falls kein Vorstellungstermin vorhanden ist: Verwende das **Dokumentdatum**.

- Wenn kein gültiges Datum erkennbar ist → `"01-01-1901"` verwenden.

4. **Gib am Ende ausschließlich das JSON-Objekt im korrekten Format zurück.**

---

### Beispiele:

1. Text: "Der ECOG-Status beträgt 0"

Denken wir Schritt für Schritt:

- Der Text enthält die Formulierung „ECOG-Status beträgt 0“, was gültig ist.

- Kein Datum vorhanden → Standardwert verwenden

Antwort:

{

"FU24M_ECOG_YN": "1",

"FU24M_ECOG_DATE": "01-01-1901",

"FU24M_ECOG": "0"

}

---

2. Text: "ECOG III laut Verlaufsnotiz vom 12.01.2022."

Denken wir Schritt für Schritt:

- „ECOG III“ ist eine gültige Formulierung → Wert = 3

- Datum ist vorhanden → 12-01-2022

Antwort:

{

"FU24M_ECOG_YN": "1",

"FU24M_ECOG_DATE": "12-01-2022",

"FU24M_ECOG": "3"

}

---

3. Text: "Die Patientin ist bettlägerig, benötigt durchgehend Hilfe."

Denken wir Schritt für Schritt:

- Es wird kein „ECOG“, „ECOG-Status“ etc. erwähnt. Nur eine Beschreibung.

- Kein ECOG-Wert darf extrahiert werden.

Antwort:

{

"FU24M_ECOG_YN": "0",

"FU24M_ECOG_DATE": null,

"FU24M_ECOG": null

}

---

4. Text: "Der Patient war vollständig abhängig von fremder Hilfe (ECOG IV)."

Denken wir Schritt für Schritt:

- Die Formulierung „ECOG IV“ wird erkannt → Wert = 4

- Kein explizites Datum → Standardwert verwenden

Antwort:

{

"FU24M_ECOG_YN": "1",

"FU24M_ECOG_DATE": "01-01-1901",

"FU24M_ECOG": "4"

}

---

5. Text: "Karnofsky 60%, Allgemeinzustand eingeschränkt."

Denken wir Schritt für Schritt:

- Keine zulässige Formulierung wie „ECOG“ enthalten.

- Kein ECOG-Wert extrahierbar.

Antwort:

{

"FU24M_ECOG_YN": "0",

"FU24M_ECOG_DATE": null,

"FU24M_ECOG": null

}

---

### Antwortformat:

{

"FU24M_ECOG_YN": "<'1' oder '0'>",

"FU24M_ECOG_DATE": "<Datum im Format DD-MM-YYYY oder '01-01-1901' oder null>",

"FU24M_ECOG": "<Zahl zwischen '0' und '4' oder null>"

}

"""

}

]
